# Bacterial regulon modeling and prediction based on systematic *cis* regulatory motif analyses

**DOI:** 10.1038/srep23030

**Published:** 2016-03-15

**Authors:** Bingqiang Liu, Chuan Zhou, Guojun Li, Hanyuan Zhang, Erliang Zeng, Qi Liu, Qin Ma

**Affiliations:** 1 School of Mathematics, Shandong University, Jinan, Shandong, China; 2 Systems Biology and Biomedical Informatics (SBBI) Laboratory University of Nebraska-Lincoln, Lincoln, NE 68588-0115, USA; 3 Department of Biology, University of South Dakota, Vermillion, SD 57069, USA; 4 Department of Computer Science, University of South Dakota, Vermillion, SD 57069, USA; 5 Department of Plant Science, South Dakota State University, Brookings, SD, 57006, USA; 6 Department of Bioinformatics, School of Life Sciences and Technology, Tongji University, Shanghai, China; 7 BioSNTR, Brookings, SD, USA

## Abstract

Regulons are the basic units of the response system in a bacterial cell, and each consists of a set of transcriptionally co-regulated operons. Regulon elucidation is the basis for studying the bacterial global transcriptional regulation network. In this study, we designed a novel co-regulation score between a pair of operons based on accurate operon identification and *cis* regulatory motif analyses, which can capture their co-regulation relationship much better than other scores. Taking full advantage of this discovery, we developed a new computational framework and built a novel graph model for regulon prediction. This model integrates the motif comparison and clustering and makes the regulon prediction problem substantially more solvable and accurate. To evaluate our prediction, a regulon coverage score was designed based on the documented regulons and their overlap with our prediction; and a modified Fisher Exact test was implemented to measure how well our predictions match the co-expressed modules derived from *E. coli* microarray gene-expression datasets collected under 466 conditions. The results indicate that our program consistently performed better than others in terms of the prediction accuracy. This suggests that our algorithms substantially improve the state-of-the-art, leading to a computational capability to reliably predict regulons for any bacteria.

Reconstruction of global transcriptional regulatory networks is a key to understand gene function and evolution, thus it is one of the critical aspects of microbial genomics, especially in the era of high throughput genome sequencing^1^,^2^,^3^,^4^. In bacterial genome, an *operon*, as a basic transcriptional unit, is a group of genes (may only one gene) arranged in tandem on the same strand of a genome, which share a common promoter and terminator^5^. To study the mechanism of transcriptional regulation, we need to know all the operons co-regulated by each of transcription factors (TFs). The term *regulon* was first introduced by Maas *et al.* in 1964, intended to name a maximal group of co-regulated operons^6^, which may be scattered in a genome without apparent patterns in terms of their genomic locations. A successful elucidation of regulons will substantially improve the identification of transcriptionally co-regulated genes encoded in a bacteria genome, realistically allowing reliable prediction of global transcription regulation networks.

Experimentally, numerous regulons have been recognized in the widely–studied model organism *E. coli*, and were stored in the RegulonDB database^7^. Nevertheless, elucidating all the regulons at a genome scale using experimental approach is highly desired. Except the high cost and time-consuming^8^,^9^, one key issue is that it is difficult to know what conditions may trigger which regulons; hence unless we can exhaustively go through all possible conditions that each triggers at least one regulon, we will not be able to observe all of the regulons experimentally^10^. Therefore computational algorithms are urgently needed, and will play an essential role in investigating all regulons encoded in a bacterial genome.

A basis for computational regulon prediction using genomic data lies in that the operons regulated by one TF should have conserved *cis* regulatory motifs (*motifs* for short) around their promoter regions. So the problem can be defined as to *find all maximal sets of operons in a genome, with each set sharing conserved motifs*. Typically, computational regulon prediction has two categories: (i) prediction of new operon members of a known regulon^11^, and (ii) *ab initio* inference of novel regulons using *de novo* motif finding strategy^3^. In early stage, most of the studies on regulon identification focused on some specific TFs, falling in the first category. For example, Tan *et al.* predicted the *CRP* and *FNR* regulons in *E. coli*, through searching for new binding sites based on the known motif profiles in RegulonDB, and then in *H. influenza* by the comparative genomics strategy^12^. The TyrR and RpoS regulons were analyzed in similar ways in *E. coli* and *Pseudomonas aeruginosa*^13^. Su *et al.* developed a program for prediction of specific regulons such as the *ntcA*^14^, *phoB*^15^, *cbbR*^16^ and σ^38^ regulons in *cyanobacteria*. Latterly, computational algorithms for general regulon prediction were developed to facilitate the systematic studies in gene regulation^17^,^18^. In the second category, for regulon prediction without known motifs, phylogenetic footprinting plays an important role through identifying conserved motifs from other relevant species (so-called *reference genomes*)^19^,^20^. An *ab initio* regulon inference should at least contain three steps: operon identification, motif prediction and clustering^21^,^22^, where a cluster corresponds to a regulon. Although the problem seems to be simple conceptually and there are numerous computational tools published^3^,^23^,^24^,^25^, it is still in its early stage with a variety of computational challenges, especially at a genome scale: (i) *de novo* motif prediction still performances poorly in terms of its accuracy, e.g. the high false positive rate. And the phylogenetic footprinting technology is still not well defined in the selection of reference genomes and measuring the evolutionary distance between any pair of genomes, limiting the usage efficacy on motif finding^23^,^24^; (ii) there is a lack of a reliable measurement for motif similarity, and current motif comparison using aligned motif profiles usually produces too many false positives^26^; (iii) better operon prediction algorithms are missing, especially utilizing the high-throughput RNA-sequencing data^24^,^27^; and (iv) current regulon prediction methods usually cluster the motif signals directly, thus leads to unreliable predictions due to randomly matching between motifs^23^. Apparently, a more ingenious design is required in the step of clustering.

In this paper, we developed an *ab initio* computational framework for elucidation of all regulons encoded in a bacterial genome (see Fig. 1). There are several unique features in our framework: (i) we derived high-quality operon predictions from the DOOR2.0 database that contains complete and reliable operons of 2,072 bacteria genomes, and we also fully considered operon structures in both motif finding and their clustering; (ii) for an operon in a target genome, we designed a new strategy to define and select its orthologous operons from the reference genomes in the same phylum but different genus with the target genome, and then refine the promoter set by eliminating redundancies. This strategy followed by our motif finding tool, BOBRO, can increase the quality of predicted regulatory motifs; and (iii) instead of calculating pair-wise similarity score for predicted motifs and then identifying regulons through motif clusters as the previous methods did, we designed a new method to build similarity relationship on operon level and then perform clustering through a heuristic graph model. Specifically, we defined a novel *co-regulation score* (CRS) among operons to evaluate the co-regulation property of them, which is much better than other similar scores that are defined through various functional and evolutionary relationships^28^,^29^,^30^. Based on the CRS, we then proposed a novel graph model for regulon identification, making the operon-clustering problem easier to solve. We evaluated and refined our methods based on the 177 documented *E. coli* regulons from RegulonDB and its genome-scale microarray gene expression data collected under 466 conditions^7^. The results suggest that, with this new implementation, the regulon prediction problem becomes substantially more solvable and our program has consistently better performance than others, especially for the 12 largest regulons each of which contains at least 20 known component operons. All the programs used in this paper, are implemented by the C computer language and are publicly released under the GPL agreement license and can be freely downloaded at http://csbl.bmb.uga.edu/DMINDA/download.php and are also implemented on a web server: http://csbl.bmb.uga.edu/DMINDA/.

## Results

### Framework Overview

**Figure 1:**
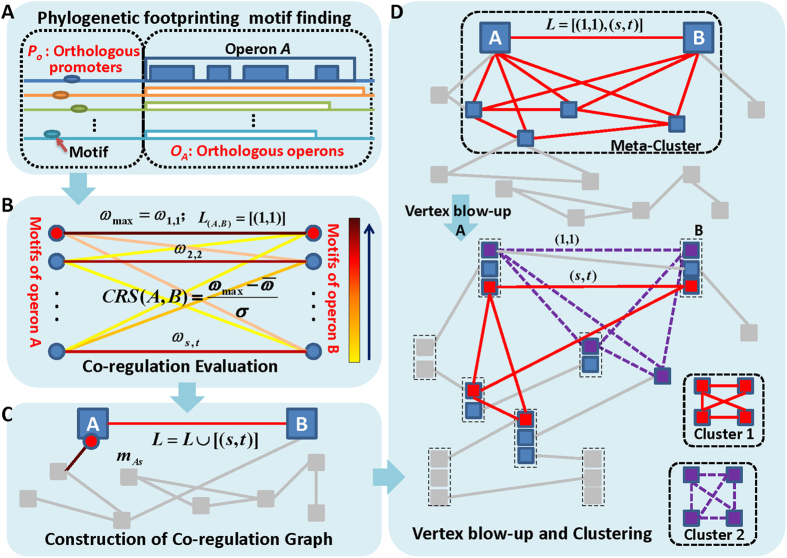
An outline of the regulon prediction framework. The blue arrows indicate data flows within this framework; and the blue rectangles represent different steps across the whole analysis. (**A**) Identification of motifs by a phylogenetic footprinting framework; (**B**) calculation of co-regulation score between a pair of operons by a highly sensitive motif similarity score. The higher the similarity score is, the darker of the corresponding edge’s color will be; (**C**) construction of graph *G* reflecting the co-regulation relationship among operons based on the similarity of their predicted motifs; and (**D**) vertex blow-up in graph *G* by re-involving motif information and clustering operons to construct accurate regulons.

The flowchart of our regulon prediction can be found in Fig. 1, and more details of the relevant algorithms are showcased in the MATERIALS AND METHODS section. All the programs mentioned in the following were implemented using the default parameters, unless otherwise indicated. We applied the proposed regulon prediction framework on *E. coli* K12, during which the co-regulation scores between any operon pairs were generated and computational significant regulons were identified. The prediction performance was assessed comparing with documented regulons from the RegulonDB database. It is noteworthy that we have implemented the framework on our in-house motif analysis web server, DMINDA^31^. Hence, the users can easily apply this proposed framework on any of 2,072 sequenced bacterial genomes included in this server.

### The orthologous operons can benefit motif finding in a phylogenetic footprinting framework

**Figure 2:**
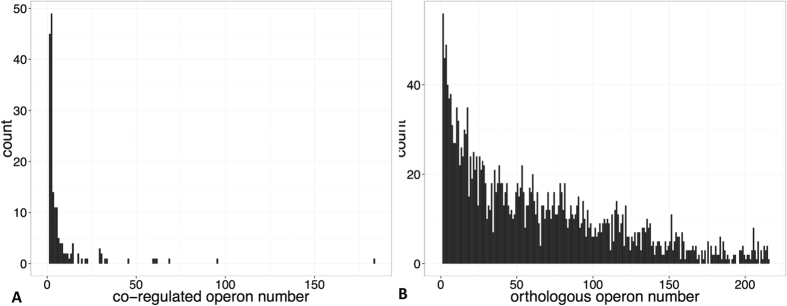
(**A**) the distribution of co-regulated operons for each operon in *E. coli* according to RegulonDB database; and (**B**) the distribution of orthologous operons for each operon in *E. coli* using 216 reference genomes.

The effective regulon identification relies on a collection of promoters containing corresponding motifs. However, such promoters are not easy to be accessed for a regulon containing only a few operons according to current regulon databases in the public domain. Specifically, only 40.4% of the 2,462 operons in *E. coli* have more than 10 co-regulated operons according to the RegulonDB database^7^ and the average number of co-regulated operons is only eight, meaning an overall non-sufficient up-streaming regulatory promoters for motif identification using the host genome solely. Intuitively, we can fix this bottleneck, expanding to 216 selected non-redundant reference genomes, through a well-designed phylogenetic footprinting framework. And the identified orthologous operons for any query operon can provide more informative promoters (see details in the MATERIALS AND METHODS section). Specifically, the average number of orthologous operons for all the 2,462 operons in *E. coli* is 84, and the percentage having over 10 orthologous operons increases to 84.3% (see details in Supplementary file 1 and corresponding orthologous promoters can be found in Supplementary file 2). The distribution comparison between co-regulated operon and orthologous operon is shown in Fig. 2. Therefore, we can claim that such a phylogenetic footprinting strategy can provide more informative promoters in motif finding for those operons involved in local regulons, without or with low number of co-regulated operons in their host genome. As described in MATERIALS AND METHODS, we applied BOBRO on these promoter sets to predict conserved motifs, which will be used to generate co-regulation relationship between operons in *E. coli*.

### CRS can represent co-regulation relationship between a pair of operons

For each pair of operons, we calculated the CRS based on a well-designed similarity comparison of their predicted motifs (Fig. 1). It elucidates the co-regulation relationship, which is the foundation of regulon identification. Here we firstly verified that the performance of CRS, in terms of representing known co-regulation relationship, is much better than the following two widely-used scores: partial correlation score (PCS) and gene functional relatedness score (GFR). The PCS and GFR are defined based on co-evolution, co-expression, and co-functional analysis among operons. Specifically, the GFR between a pair of genes is calculated from three different perspectives: phylogenetic profile analysis, gene ontology analysis, and gene neighborhood property along the genome^28^. Here we applied the concept in GFR to operon level by averaging all the involved GFRs in a pair of operons. And the PCS between a pair of operons was calculated as follows. For an operon *A* in the genome of *E. coli* K12, we constructed an evolutional 0–1 vector across the 216 reference genomes, where 1 means there is an orthologous operon of *A* in a specific genome and 0 means the opposite. The PCS between a pair of operons in *E. coli* K12 is measured based on the two corresponding 0–1 vectors using the similar strategy in literatures^29^,^30^, which can elucidate their evolution similarity by removing indirect relationship in a large network.

**Figure 3:**
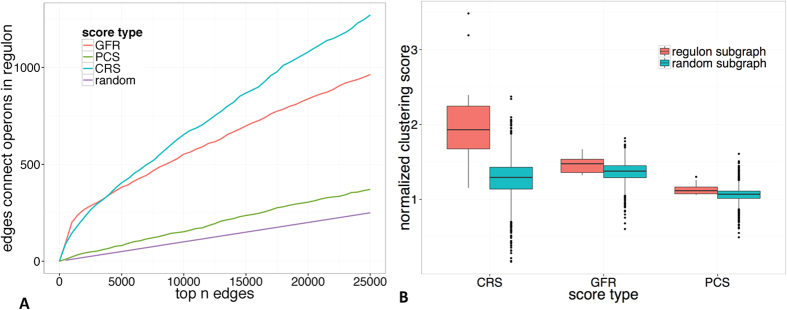
Performance comparison of CRS, PCS, and GFR. (**A**) The *efficiencies* of these scores along with a randomly selection, where edges correspond to operons pairs as defined in graph *G*. (**B**) Comparison of the normalized *clustering coefficient* (*nCC*) regardning known regulons in *G* keeping the top 25,000 operons pairs. For each score out of the three, the normalized *CCs* of a group of randomly selected vertex sets with similar sizes are listed as background. For CRS, the nCCs of known regulons are much higher than randomly selected operon sets, with the the *P*-value of the Wilcoxon test as 1.7e-09. And the corresponding *P*-vaules for PCS and GFR are 0.0009 and 0.0018, respectively.

Let *G* be the graph with operons as vertices and edges connecting each pair of vertices. The weight of an edge could be one of the three kinds of scores (CRS, PCS and GFR) between the corresponding two operons. Firstly, the *efficiency* of a score is defined as the numbers of operon pairs, being in known regulons, in top *n* operon pairs (*n* is from 0 to 25,000 by 500 intervals) out of totally 3,247,426 operon pairs ranked according to this score. As shown in Fig. 3A, the *efficiency* of CRS is obviously better than PCS, GFR, and a randomly selection; and the *efficiency* of CRS has the fastest increasing rate among all the scores as *n* increases from 0 to 25,000. GFR shows better performance in limited scale (≤4,000), because it takes more information into consideration, in support of the identification of the most significant co-regulatory relationships. This phenomenon indicates that the CRS has the best signal-noise ratio in to-be-selected operon pairs and will perform better in the following regulon prediction compared to the others, especially in genome scale application. See details in Supplementary Table S1.

On the other hand, we compared above three scores in terms of the *normalized Clustering Coefficient* (*nCC*) of 17 known regulons (containing more than 20 operons). The normalized clustering coefficient can be used to infer the potential capability of a specific set of vertices to be identified as a cluster in a graph. Intuitively, a regulon corresponds to a sub-graph *H* in the graph *G*, with operons as vertices and 25,000 selected edges. *E*(*H*) and *V*(*H*) represent the edge set and vertex set in *H* and |*X*| means the number of elements in a set *X*. The *Clustering Coefficient* (*CC*) is a measure of the degree to which vertices in a graph tend to be clustered together based on triplets of vertices^32^. Specifically, a triplet consisting of three vertices is called *close* if the three vertices are connected by three edges; and *CC*(*H*) is calculated as the number of closed triplets over the total number of connected triplets in *H*. Then *CC*(*H*) is normalized by


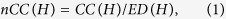


where *ED*(*H*) represents the edge density of *H*, and can be calculated as


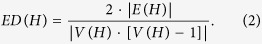


Intuitively, a set of vertices with higher *nCC* tend to be identified as a cluster much easier. From the comparison analysis in Fig. 3B, we found that (i) a regulon has the largest superiority compared to same-size and randomly selected operons using CRS (with the P-value as 1.7e-9) than using the other two scores; and (ii) the *nCC*s of 17 regulons based on CRS are significantly higher than those based on PCS and GFR (see Supplementary Table S2 for details), indicating CRS will have better performance in the following regulon predictions.

### The performance of our regulon prediction

**Figure 4:**
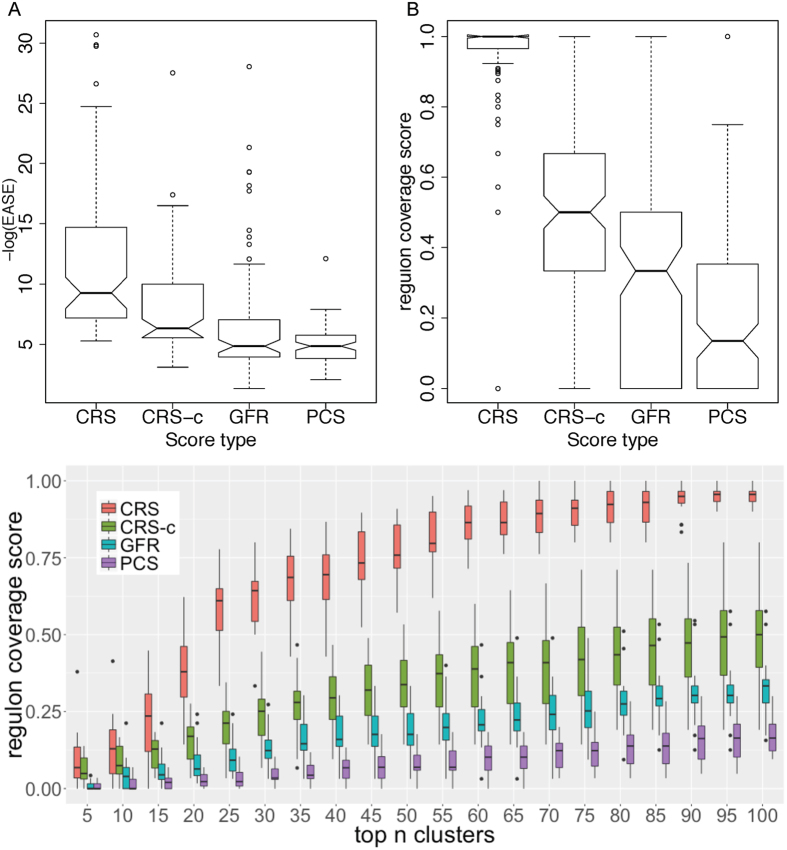
Performance of regulon prediction. (**A**) Comparison of *EASE* distribution among CRS, CRS-c, GFR and PCS clusters. For each cluster, only the smallest EASE score with a regulon is kept. Note that the EASE score in y-axis is in −log() scale. (**B**) Comparison of the regulon coverage scores (RCSs) among CRS, CRS-c, GFR and PCS clusters. Each box represents the RCSs of the 177 documented regulons. (**C**) Comparison of the regulon coverage scores of CRS, CRS-c, GFR and PCS regarding the largest 12 known regulons. Each box represents the regulon coverage scores of these 12 regulons covered by the union of top *n* clusters (*n* = 10, 20, …, 100).

Based on the CRS, our framework built a co-regulated graph and generated operon clusters for regulon prediction. In this step, instead of directly clustering on operons, we involved the predicted motifs to construct a new vertex-blowup graph model (Fig. 1C), and then took the compact cliques to generate primary operon clusters, represented by CRS-c in Fig. 4. It is noteworthy that the cliques are too strict for regulon prediction as the operons in one regulon may share weakly conserved motif patterns. Hence, we took the corresponding motifs to scan the whole genome, and optimize the corresponding operon clusters by making the operons with significant motif hits involved, otherwise excluded. The final optimized operon clusters were recognized as predicted regulons in our study (represented by CRS in Fig. 4).

To further access the regulon identification power of our new framework, we compared its predicted regulons with the operon clusters identified based on GFR and PCS (represented by GFR, and PCS in Fig. 4). These clusters are generated by widely-used clustering tool MCL^33^,^34^. Here, we used 177 documented regulons in the RegulonDB database as benchmark data to evaluate the performance of predictions. We took the top 100 clusters for each of the three scores into consideration (see Supplementary Table S3). For an individual cluster, we assess the statistical significance of the coincidence level between it and a specific regulon using a modified Fisher Exact test, *EASE*^35^. Specifically, for the whole genome *M* and a known regulon *R*, if a predicted regulon *P* is random, then the probability of *P* and *R* sharing exactly *n* (>1) operons is:


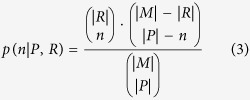


where |*X*| means number of operons in set *X*. The *EASE* score is computed as:


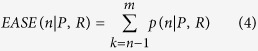


where *m* = min{|*P*|, |*R*|}. Intuitively, the smaller an *EASE* score is, the more significant matching between a predicted operon cluster and a known regulon has. Since a predicted regulon could overlap with many known regulons, only the smallest *EASE* score was kept. As shown in Fig. 4A, the primary operon clusters, CRS-c, using the score designed by us have much lower *EASE* scores than those of the other two scores, where the *EASE* scores are transferred to the −log() scale. And the optimized clusters can reach even better performance (More details can be found in Supplementary Table S4).

On the other hand, we defined the *regulon coverage score* for a known regulon as Ma *et al.* did^26^ to measure the coverage of individual operons of a regulon by certain predicted operon clusters, which provides another way to evaluate the consistency between predicted clusters and known regulons. Note that the larger the score is, the more component operons of the corresponding regulon this predicted clusters could correctly cover. Here, we used the top *n* (*n* = 10, 20, …, 100) clusters to calculate these scores for all the known 177 regulons, and the comparison among these four sets of clusters (CRS, CRS-c, GFR and PCS) are shown in Fig. 4B. It is very clear that the regulon coverage scores corresponding to CRS are remarkably higher than those for the other two scores, especially for optimized ones. More details can be found in Supplementary Table S5. Furthermore, Fig. 4C showcases a comparison among the clusters generated by the three functional relatedness scores in terms of the 12 largest regulons each of which contains at least 20 component operons: CRP, Fur, FNR, IHF, Fis, Lrp, CpxR, LexA, NsrR, NarL, Cra and ArcA. We can see that CRS based clusters still keep the significant performance advantages. Further analysis showed the coverage scores of the small regulons tend to have large variance between 0 and 1, especially for those having less than five operons. See Figure S2 in Supplementary materials for more details.

### Validation of developed methods based on available microarray gene expression data

**Figure 5:**
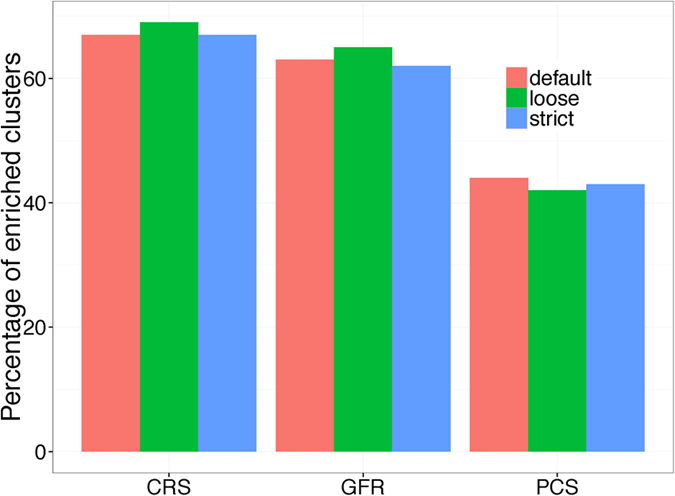
Percentage of predicted regulons supported by the co-expression gene sets. Each of CRS, GFR and PCS identified regulons combined with a clustering method. Three kinds of co-expression gene sets correspond to different parameters of QUBIC (Table 1).

Since the 177 regulons documented in the RegulonDB probably only represent a portion of all the regulons encode in *E. coli* K12, we also validated our predicted regulons against the co-expression gene sets identified by applying our in-house bi-clustering program, QUBIC^36^,^37^ on the genome-scale microarray gene expression data. Because co-expression indicates co-regulation, these co-expressed gene sets could be used for validation. For each of our predicted regulons, we compute an *EASE* score in the same way as did in above section, using the co-expressed gene sets inferred from microarray data instead of documented ones. If the corresponding *EASE* score of a predicted regulon is under a certain cutoff (1e-3), its member genes tend to be co-expressed under specific conditions and it is determined as a supported one by the transcriptomic data. It is also worth noting that, three different parameters of QUBIC were used to generate co-expressed gene sets with various scales for our regulon validation, as the scale of co-expressed gene sets does have impacts on the *EASE* score calculation and different scales may lead to different conclusion based on the same predicted regulons. The details of QUBIC’s parameters and matching identified bi-clusters can be found in Table 1. As shown in Fig. 5, over 65% of our predicted regulons are supported by all three types of bi-clusters, which are consistently higher than those results using the other two scores. Please see details in Supplementary Table S6. The performance of the GFR is slightly worse than our CRS score and much better than the PCS. It is not surprised because GFR score already integrated the co-expression information when it is calculated. This also demonstrates that our CRS score, based on genomic sequences alone, and has a very competitive prediction power.

### Implementation on DIMINDA web server

To facilitate the usage and application of our new framework, we have implemented and integrated it into DMINDA^31^. It is an integrated user-friendly web server for DNA motif prediction and analyses based on motif finding programs BOBRO^38^, motif analysis package BOBRO2.0^39^, and the DOOR2.0 database containing operons for more than 2,000 prokaryotic genomes^40^. We listed all 2,072 prokaryotic genomes on the web server, and the users can perform our new computational framework on any of them to calculate CRS and predict regulons easily. An illustration of how to use the new framework in DMINDA can be found in the Supplementary Appendix 1.

## Discussion

While numerous regulons have been experimentally identified in a few model organisms including *E. coli* K12, the full elucidation of all the regulons encoded in this or any bacterial genome may have to rely heavily on computational approaches. The reason is that experimental detection of a regulon depends on the ability to design the conditions that can activate the regulon to be experimentally observable. However, there is no information that can guide the experimental design to have every regulon in a bacterium be activated. In this paper, we have designed a computational tool for regulon identification in bacterial genomes, by integrating genome-scale motif analyses and accurate operon identification into a phylogenetic footprinting framework. The key intellectual contributions of this framework include (i) the discovery of a new co-regulation score (CRS) between any pair of operons, which complements the known linkages among operons in the same regulon, and has made it possible, in conjunction with the conserved motif information, to solve the regulon problem at a genome scale; (ii) a suite of programs for solving a number of important and currently not well solved bacterial genome analysis problems, namely mapping of orthologous genes between two genomes, prediction of motifs with high efficacy and prediction of regulons; and (iii) a collection of new regulons in *E. coli* K12. With the new framework, people can identify all encoded regulons for any newly sequenced complete genome systematically, which could be applied to elucidate global transcriptional regulatory network and associated metabolic pathways. In addition, people can identify regulons responsive to particular stimuli, whose operon members have their expression values changed, i.e. up- or down-regulated, in the corresponding high-throughput transcriptomic data. Upon getting genome-scale regulons identified in substantial organisms, it is a good opportunity for researchers to understand how the regulatory systems have evolved in bacteria through comparative genomics studies. It is noteworthy that our whole framework can be easily extended to regulon identification in eukaryotic genomes by withdrawing the operon concept in the orthology mapping step. As there are more and more Chip-seq data accumulated in human and mouse, we expect that it should have similar performance as in bacterial genomes.

However, there is still a big room to improve in computational prediction of regulons. The key issue is that our understanding about regulons is not much beyond its basic definition, i.e., operons sharing conserved motifs; and this information by itself is too weak to allow the current motif-finding methods to reliably identify all the co-regulated operons encoded in a genome. It is not hard to imagine that by chance, some sequence segments across some promoters may look alike among over 2,000 *E. coli* promoters, while at the same time, some regulons have only a few component operons, i.e., having conserved motifs among just a few promoters. Hence it is very challenging to distinguish the true motifs of local regulons from the accidental ones. Some global regulators’ binding sites show several branch of conservation thus may be divided into several local clusters^25^. Also for some regulons, their motifs tend to be less conserved^41^ and are hard to be identified, which makes the motif prediction suffers the high false positive rate in their prediction. We believe that a key to overcome above issues is through identification of new and more specific characteristics of regulons, based on which more reliable predictions could be made. For example, documented motifs tend to cluster together with other motifs in their genomic neighborhoods^42^. In future, we plan to explore various scoring schemes to include such information, among other relevant information that we will explore. Meanwhile, with the advent of large-scale ChIP-seq data in the prediction of TF binding sites^43^,^44^, people can reliably assess the possibility for each nucleotide in a given promoter to be occupied by specific TF. Such information can help to decrease the false positive rate in motif prediction and make the computational prediction of regulons more reliable.

## Materials and Methods

### Data Preparation

All the data included in this paper is publicly available. We used *E. coli* K12 as the target genome for assessing our algorithms and other selected 216 bacterial genomes as its reference genomes, which were downloaded from the NCBI (released as of November 2011). These reference genomes belong to the same phylum and different genus of *E. coli*, which were used to identify orthologous genes as in literatures^45^,^46^. To evaluate predicted regulons, we downloaded gene expression data of *E. coli* collected under 466 conditions from the M3D database^47^. In addition, we retrieved 2,462 operons of the target genome *E. coli* K12 and 454,181 operons for those 216 reference genomes from the operon database DOOR2.0^40^,^48^. This database contains predicted operons for 2,072 organisms with 2,205 chromosomes and 1,645 plasmids, consisting of a total of 3,902,851 operons.

### Identification of *cis* regulatory motifs by a phylogenetic footprinting framework

The basic assumption of the phylogenetic footprinting technique is that orthologous genes across related organisms tend to use orthologous transcription regulators, and their binding sites are more conserved at the DNA sequence level than surrounding non-functional sequences^24^,^49^. It usually has three steps: orthology identification, promoter collection, and motif finding (Fig. 1A).

#### Orthology identification for genes in target genome

Identification of orthologous genes between two genomes is a foundation for phylogenetic footprinting. Here we used our in-house program GOST^50^, which takes the operon information into consideration as a functional guidance to perform orthology identification. Specifically, GOST first assigns the genes as *working partners* of each other if they belong to the same uber-operon^51^, and then performs orthology mapping between two bacterial genomes using a constrained maximum matching algorithm on a bipartite graph. Finally, the genes are considered as *orthologous* genes if they have *working partners* that are homologous across the two genomes.

#### Collection of orthologous promoters based on orthology and the operon structure

For an operon *A* in *E. coli*, the new framework first constructed an orthologous graph *G*_*A*_. The vertices in *G*_*A*_ represent the operon *A* and the other operons across all the reference genomes that share at least one orthologous gene with *A*. For any pair of vertices in *G*_*A*_, we added an non-weighted edge if the two corresponding operons share at least one orthologous gene; and then identified the largest operon group containing *A* using MCL^34^, denoted as *O*_*A*_. All the other operons in *O*_*A*_ are called *orthologous operons* of *A*. Finally, we extracted the upstream sequence set *P*_*A*_, according to the operon group *O*_*A*_, as the *orthologous promoters*. Specifically, we used upstream 300 bps or the whole intergenic region (if less than 300 bps) as promoters starting from translation start site of each of these operons (the transcription start sites are typically not easy to be collected). In addition, we eliminated redundant promoters by CD-HIT^52^, as the reference genomes cannot be too close to target genome, otherwise the motifs in promoters are not sufficiently better conserved than surrounding nonfunctional sequence.

#### Application of motif finding on orthologous promoter set

We ran our in-house motif-finding tool, BOBRO^26^,^31^, on the orthologous promoter set to find motifs. BOBRO had been tested on phylogenetic footprinting data and showed consistently better performance than other existing tools^38^. The identified motifs are used in calculating co-regulation scores between any pair of operons in the next sub-section.

### Calculation of the Co-Regulation Score (CRS) between a pair of operons

Our regulon prediction algorithm is based on the observation that any two conserved motifs (a motif presents as a set of gapless aligned motif instances in algorithm design) without functional relationship are statistically hard to be similar to each other. First of all, we used BOBRO to identify all statistically significant motifs in regulatory promoters *P*_*A*_ for each operon *A* in target genome as described in previous section. Then the motif similarity score can be calculated based on their corresponding position weight matrix (PWM)^26^. For any pair of operons, *A* and *B*, in the target genome, the two sets of identified motifs in their regulatory promoters are denoted as *M*_*A*_ = {*m*_*A*1_, *m*_*A*2_, …, *m*_*As*_} and *M*_*B*_ = {*m*_*B*1_, *m*_*B*2_, …, *m*_*Bt*_}, respectively (Fig. 1B). We calculated the similarity scores for each pair *m*_*Ai*_ and *m*_*Bj*_ (*i* = 1, …, *s* and *j* = 1, …, *t*), which are denoted as *ω*_*i*,*j*_. Now we can define a *Co-Regulation Score* (CRS) between *A* and *B* along as following,


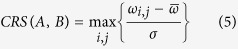


where 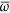 and *σ* represent the average and variance of similarity scores between any pair of motifs from *M*_*A*_ and *M*_*B*_ respectively (Fig. 1B). Intuitively, the larger the CRS is, the higher probability of co-regulatory relationship these two operons have. In order to eliminate the effect of insignificant motif pairs, we set CRS to zero if the maximum *ω* between two operons is not higher than a given threshold (we use 0.5 in this analysis according to our previous study^39^). Besides, we assigned a label set *L* for each operon pairs to record the labels of motif pairs, which represent the potential co-regulation relationship of two operons. Initially, the *L* for each two operons contains the indexes of motif pairs with maximum CRS (>0). For example, *L* = [(1, 1)] for operon A and B because *ω*_*max*_ = *ω*_1,1_ (Fig. 1B). It is worth noting that, two operons can be co-regulated by different TFs, thus some other significant motif pairs, besides the one reaching CRS, should also be included. The label set *L* will be expanded in the following graph construction section, aiming to retrieve these significant motif pairs and get rid of randomly generated noises.

### Construct of co-regulated graph *G*

Based on the CRSs in the previous sub-section, we can construct a completed graph *G* in which each vertex represents an operon and the weight of each edge is the CRS (along with its label set) between the two corresponding operons. This graph represents the co-regulated relationship between any two operons and the normalized CRS scores will facilitate the following regulon construction. It is well known that one operon can be regulated by various TFs; hence, for an operon *A*, different motifs in *M*_*A*_may reach CRS with different operons. This point will be used to improve the graph *G* by extending the label set *L* between any two operons. First, the motifs showing in at least one label set of *G* are denoted as *effective* motifs. Next, the labels of a motif pair (*m*_*Ai*_, *m*_*Bj*_) will be added to *L*, if (i) 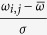 is higher than a specific threshold (we used 2 in this paper), and (ii) at least one of the two motifs is the *effective* motif. Taking operons A and B as an example (Fig. 1C), suppose that the motif pairs (*m*_*A*2_, *m*_*B*2_) and (*m*_*As*_, *m*_*Bt*_) have the second and third largest similarity scores with 
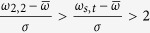
; and only *m*_*As*_ is annotated as an *effective* motif in above graph *G*. In this situation, the labels (*s*, *t*) will be added to *L*, while (2, 2) is excluded (Fig. 1B,C). The above strategy is standing on following view that the real co-regulated motif pairs could only be defeated by another co-regulated motif pairs or noise with higher scores by chance, between a pair of its regulating operons. And the updated label sets in *G* will benefit the regulon identification showcased in the next sub-section.

### Regulon identification through clustering operons on a novel graph model

Intuitively, the operons belonging to the same regulon will compose a heavier cluster in *G* (that is, a sub-graph with higher weight edges). However, a heavy cluster has not necessary to be a regulon, since one operon may belong to several regulons thus the heavy edges in the heavy cluster may be derived from different motifs, which are called meta-clusters in this study (Fig. 1D). In fact, the regulons encoded in a genome could be overlapped or even inclusive. Therefore, the clustering for regulon prediction should first allow overlapped clusters, and also be capable of decomposing meta-clusters. It brings great challenges in algorithm design. Besides, identifying all heavy sub-graphs in a weighted graph itself is NP-hard. Hence, in this paper, we designed a heuristic clustering algorithm to identify regulons.

We attempted to address the above issues by building a new graph from *G*. Firstly, we blew up each vertex in *G* into several new vertices corresponding to a set of motifs of this particular operon and their related label set *L*; then set the edge weight and modified it based on the neighborhood similarity of the motif pairs, with the biological insight that two vertices belonging to a same regulon should more likely to have similar neighbors. Finally, we identified heavy sub-graphs in the new graph to infer regulons.

#### Step 1: Construction of new graph G′

We reconstructed a new graph *G′,* by splitting each vertex in *G* into a set of new vertices, which correspond to the motifs included in all its related label sets. And the motif pairs in the label sets of *G* are connected as the edges of *G′* (Fig. 1D). The weight of the edge between vertices *A*_*i*_ and *B*_*j*_ corresponding to motifs *m*_*Ai*_ and *m*_*Bj*_ will be assigned as


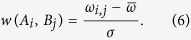


Obviously, the construction of *G* eliminated most of edges derived from randomly matching between motifs, thus will greatly reduce false positives in regulon prediction. More importantly, in the new graph *G′*, the meta-clusters have been decomposed and each heavy cluster in *G′* will correspond to a regulon, without any overlap with other regulons. Hence, we can solve a much easier clustering problem for identifying heavy sub-graphs in *G′* than in *G*, without introducing too much false positives.

#### Step 2: Modification of graph G′

For any edge (*A*_*i*_, *B*_*j*_) in *G′*, its weight is modified as following:


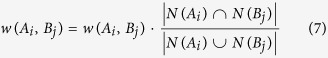


where *N*(*x*) represents all the vertices adjacent with vertex *x* and |*X*| represents the number of elements in *X*. Then we only kept the top *α*% edges in *G′* according to their weights. For each vertex, we retrieved top *β* adjacent edges if less than *β* adjacent edges left after deletion. The default values for *α* and *β* are 20 and 10 in this study, respectively. The modification should make the graph model represent the biological reality more accurately. Actually the strategy for defining similarity in networks used here has been proved to be effective in previous study^26^. In the next two steps, we will identify cliques in the modified graph and then expand them to heavy sub-graphs.

Note that the idea of finding regulons through finding cliques or near cliques has been widely used in the existing regulon-finding programs (22–23). However, there are two technical issues: (i) the constructed graphs are generally quite noisy, leading to high false positive predictions, and (ii) the cliques alone are not adequate to capture the majority of the regulons, hence leading high false negative predictions. We have addressed the issue (i) by the well-designed CRS and newly constructed graph *G′* with a high signal-to-noise ratio; and we have addressed (ii) by decomposing the regulon identification problem into two steps: finding cliques in the representing graph *G′* and using them as the seeds of regulons to find the other regulon members through refining and expanding the cliques.

#### Step 3: Clique finding in G′

We identified all disjoint maximal cliques in *G′* using the following greedy approach:

1. set *C* to be empty;
2. choose an edge (*u, v*) in *G′* with the largest *N*(*u*) ∩ *N*(*v*);
3. add *u* and *v* to the current clique *C*;
4. repeat the above procedure on the sub-graph induced by *N*(*u*) ∩ *N*(*v*) until the sub-graph is empty;
5. remove the current clique from *G′*, and repeat this step on the remaining graph for *M* times (the default is *M* = 300 according to the approximate number of known TFs in bacterial genomes).

#### Step 4: Refinement and optimization of identified cliques

For each clique *C* identified in *Step* 3, its corresponding operons are denoted as *R* and its most statistical significant motif as *m*_*c*_ according to the P-value derived by BOBRO. Then motif *m*_*c*_ is scanned against the orthologous promoter sets of all the operons using BBS, which also provides P-value for the scanning results. For operon *A* belongs to *R*, if corresponding P-value of scanning results is larger than 0.05, then *R* = *R*\*A*. For any operon *A* not in *R*, if its corresponding P-value of scanning results is less than 0.05, then add *A* to *R.* Finally, the cliques will be expanded to heavy sub-graphs, from which the candidate regulons will be generated.

## Additional Information

**How to cite this article**: Liu, B. *et al.* Bacterial regulon modeling and prediction based on systematic *cis* regulatory motif analyses. *Sci. Rep.* **6**, 23030; doi: 10.1038/srep23030 (2016).

## Supplementary Material

Supplementary Information

Supplementary Dataset 1

Supplementary Dataset 2

## Figures and Tables

**Table 1:** Three types of bi-clusters (default, loose and strict) correspond to the different parameters of c (conservedness of the bi-clusters), 0.95, 0.90 and 1.00, respectively.

Bi-cluster type	#bi-cluster	Largest size	Smallest size	Average size
default	500	526	32	105.9
loose	500	588	32	122.7
strict	500	250	41	66.8
