# Supplementary material for: Bacterial regulon modeling and prediction based on systematic cis regulatory motif analyses
Source: Sci Rep. 2016 Mar 15;6:23030. doi: 10.1038/srep23030 (PMC4792141; doi:10.1038/srep23030)

Supplementary Materials

Bacterial regulon modeling and prediction based on systematic *cis* regulatory motif analyses

Bingqiang Liu[1], Chuan Zhou[1], Guojun Li[1], Hanyuan Zhang[2], Erliang Zeng[3, 4, 7], Qi Liu[6], Qin Ma[5, 7, *]

[1]School of Mathematics, Shandong University, Jinan, Shandong, China,

[2]Systems Biology and Biomedical Informatics (SBBI) Laboratory University of Nebraska-Lincoln. Lincoln, NE 68588-0115, USA,

[3]Department of Biology, University of South Dakota, Vermillion, SD 57069, USA,

[4]Department of Computer Science, University of South Dakota, Vermillion, SD 57069, USA,

[5]Department of Plant Science, South Dakota State University, Brookings, SD, 57006, USA,

[6]Department of Bioinformatics, School of Life Sciences and Technology, Tongji University, Shanghai, China, and

[7]South Dakota Biochemical Spatiotemporal NeTwork Resource (BioSNTR), SD, USA. §Corresponding author.

Bingqiang Liu: bingqiangsdu@gmail.com

Chuan Zhou: zhouchuan121@gmail.com

Guojun Li: guojunsdu@gmail.com

Hanyuan Zhang: unlzhhy@huskers.unl.edu

Erliang Zeng: Erliang.Zeng@usd.edu

Qi Liu: qiliu@tongji.edu.cn

Qin Ma: qin.ma@sdstate.edu

To whom correspondence should be addressed:

Tel: +1 605-688-6315,

Email: qin.ma@sdstate.edu.

Table S1. Comparison analysis of CRS, GFR and PCS according to top X edges

Step	Type	In_Reg_Edge	Regulon coverage
500	CRS	91	25
1000	CRS	145	32
1500	CRS	187	34
2000	CRS	227	35
2500	CRS	265	38
3000	CRS	295	38
3500	CRS	318	39
4000	CRS	345	43
4500	CRS	378	44
5000	CRS	405	45
5500	CRS	426	45
6000	CRS	453	46
6500	CRS	475	48
7000	CRS	499	49
7500	CRS	518	50
8000	CRS	546	50
8500	CRS	574	53
9000	CRS	602	53
9500	CRS	629	54
10000	CRS	653	55
10500	CRS	674	55
11000	CRS	687	55
11500	CRS	704	55
12000	CRS	727	56
12500	CRS	751	58
13000	CRS	773	58
13500	CRS	799	58
14000	CRS	820	60
14500	CRS	851	61
15000	CRS	868	61
15500	CRS	883	61
16000	CRS	899	61
16500	CRS	927	61
17000	CRS	958	61
17500	CRS	978	62
18000	CRS	1011	62
18500	CRS	1026	63
19000	CRS	1044	63
19500	CRS	1062	64
20000	CRS	1080	64
20500	CRS	1100	64
21000	CRS	1119	64
21500	CRS	1139	64
22000	CRS	1149	64
22500	CRS	1165	65

23000	CRS	1181	65
23500	CRS	1201	65
24000	CRS	1230	65
24500	CRS	1247	65
25000	CRS	1271	66
500	GFR	97	18
1000	GFR	201	21
1500	GFR	241	26
2000	GFR	268	30
2500	GFR	286	31
3000	GFR	302	33
3500	GFR	320	33
4000	GFR	343	41
4500	GFR	363	41
5000	GFR	382	43
5500	GFR	394	43
6000	GFR	414	47
6500	GFR	430	50
7000	GFR	444	51
7500	GFR	466	51
8000	GFR	485	53
8500	GFR	499	53
9000	GFR	514	54
9500	GFR	530	54
10000	GFR	552	55
10500	GFR	562	56
11000	GFR	578	58
11500	GFR	590	58
12000	GFR	608	58
12500	GFR	616	58
13000	GFR	631	60
13500	GFR	653	60
14000	GFR	667	60
14500	GFR	682	60
15000	GFR	698	61
15500	GFR	713	61
16000	GFR	725	62
16500	GFR	740	65
17000	GFR	760	65
17500	GFR	774	65
18000	GFR	784	65
18500	GFR	799	65
19000	GFR	809	65
19500	GFR	824	65
20000	GFR	838	65
20500	GFR	851	65
21000	GFR	863	65
21500	GFR	872	65
22000	GFR	886	66

22500	GFR	905	66
23000	GFR	920	66
23500	GFR	929	66
24000	GFR	938	66
24500	GFR	950	66
25000	GFR	963	67
500	PCS	10	6
1000	PCS	22	13
1500	PCS	34	16
2000	PCS	42	16
2500	PCS	48	19
3000	PCS	52	20
3500	PCS	60	20
4000	PCS	67	22
4500	PCS	77	26
5000	PCS	81	26
5500	PCS	92	28
6000	PCS	101	29
6500	PCS	106	29
7000	PCS	115	30
7500	PCS	121	31
8000	PCS	127	31
8500	PCS	135	32
9000	PCS	144	33
9500	PCS	148	33
10000	PCS	152	33
10500	PCS	160	33
11000	PCS	169	34
11500	PCS	172	35
12000	PCS	184	35
12500	PCS	194	38
13000	PCS	207	38
13500	PCS	214	38
14000	PCS	220	39
14500	PCS	230	40
15000	PCS	236	40
15500	PCS	241	41
16000	PCS	247	42
16500	PCS	255	42
17000	PCS	265	44
17500	PCS	276	45
18000	PCS	281	45
18500	PCS	289	45
19000	PCS	295	45
19500	PCS	300	45
20000	PCS	305	45
20500	PCS	312	45
21000	PCS	321	45
21500	PCS	328	45

22000	PCS	332	45
22500	PCS	335	45
23000	PCS	343	46
23500	PCS	355	47
24000	PCS	357	48
24500	PCS	365	49
25000	PCS	371	49

Table S2. The transitivity scores of 17 regulons (containing >20 operons) regarding three standards.

score	regulon	nodes	edges	edge_density	average_score	transitivity
**CRS**	FNR	94	1137	0.26	17.782	0.389
**CRS**	Fis	57	417	0.261	17.22	0.349
**CRS**	H-NS	56	466	0.303	17.439	0.459
**CRS**	FruR	32	152	0.306	19.247	0.429
**CRS**	Lrp	28	114	0.302	17.007	0.42
**CRS**	LexA	25	112	0.373	18.8	0.612
**CRS**	NsrR	31	121	0.26	17.732	0.34
**CRS**	CRP	176	3674	0.239	16.856	0.309
**CRS**	IHF	67	613	0.277	17.335	0.365
**CRS**	MarA	20	26	0.137	17.397	0.221
**CRS**	PhoP	28	73	0.193	17.492	0.315
**CRS**	ArcA	58	434	0.263	16.809	0.323
**CRS**	NarL	29	132	0.325	18.276	0.435
**CRS**	Fur	45	430	0.434	20.066	0.668
**CRS**	CpxR	29	87	0.214	16.968	0.296
**CRS**	SoxS	20	24	0.126	15.442	0.375
**PCS**	FNR	81	1230	0.38	0.006	0.409
**PCS**	Fis	47	381	0.352	0.006	0.383
**PCS**	H-NS	36	242	0.384	0.005	0.408
**PCS**	FruR	30	157	0.361	0.005	0.418
**PCS**	Lrp	22	98	0.424	0.005	0.413
**PCS**	LexA	18	58	0.379	0.007	0.398
**PCS**	NsrR	26	110	0.338	0.005	0.384
**PCS**	CRP	136	3403	0.371	0.005	0.396
**PCS**	IHF	54	517	0.361	0.005	0.382
**PCS**	MarA	16	48	0.4	0.006	0.388
**PCS**	PhoP	18	65	0.425	0.005	0.454
**PCS**	ArcA	48	412	0.365	0.005	0.388
**PCS**	NarL	26	139	0.428	0.006	0.442
**PCS**	Fur	39	315	0.425	0.005	0.47
**PCS**	CpxR	21	81	0.386	0.006	0.396
**PCS**	SoxS	16	53	0.442	0.005	0.429
**GFR**	FNR	73	1774	0.675	0.277	0.819
**GFR**	Fis	49	683	0.581	0.298	0.763
**GFR**	H-NS	44	453	0.479	0.205	0.737
**GFR**	FruR	31	365	0.785	0.904	0.904
**GFR**	Lrp	22	156	0.675	0.548	0.855
**GFR**	LexA	14	71	0.78	0.935	0.818
**GFR**	NsrR	18	82	0.536	0.248	0.716
**GFR**	CRP	154	7385	0.627	0.24	0.771
**GFR**	IHF	56	747	0.485	0.193	0.732

**GFR**	MarA	12	54	0.818	0.386	0.891
**GFR**	PhoP	17	79	0.581	0.316	0.783
**GFR**	ArcA	52	1016	0.766	0.305	0.877
**GFR**	NarL	26	185	0.569	0.181	0.727
**GFR**	Fur	34	415	0.74	1.159	0.824
**GFR**	CpxR	21	116	0.552	0.312	0.716
**GFR**	SoxS	14	65	0.714	0.438	0.815

Table S3. The identified top 100 operon clusters based on CRS, GFR and PCS.

CRS1 16130967-90111214-16129075-16130089-16129216-16130504-16130505-16129217-16130504-16130505-16128633-90111562-16131133-90111562-16131133-90111444-16129202-16129203-16131469-145698229-94541112-16131028-16129217-16129166-16129216-16131242-16131243-16129185-16129186-16131244-90111444-16130703-145698279-145698297-16130321-16130320-16128604-16128633-16131243-16131242-16129771-16132089-16132090-16131680-49176425-16129202-16129203-16130119-90111214-16129075-16129487-16128757-16128607-145698298-345452716-16129732-16130518-16129724-16129733-16129972-16129072-16130081-16131469-49176351-90111079-16130987-16130988

CRS2 16129804-90111591-90111591-90111591-90111591-16131238-16132089-16132090-226524724-49176350

CRS3 16128194-94541120-16131810-16129858-49176415-16130540-90111716-90111364-16130281-16128266-16130720-226524751-145698249-49176415-16129623-16129623-226524727

CRS4 16129801-16129324-16129802-145698329-16128095-226524720-16128564-49176035-16128747-162135920-16129146-16129950-49176247

CRS5 16128564-49176035-145698329-16129024-16131665-16129146-16129950-16129139-16129140-49176247-145698274-90111626-90111626

CRS6 16128188-16131548-49176459-16128938-16131919-90111508-90111152-16129266-16128406-16128471-16129444-16128188-16129990-90111448-16130424-16131597-16132144-16129035-16129036-16129695-49176301-49176415-16128101-16129730-16130134-16130377-145698300-16131530-49176448-16130021-16130274-16130444-90111507-16131961-16131921-16130196-90111563-16129298-16131919-16128045-16131656-16128269-16128829-90111430-16128275-16128095-16128556-90111167-16129643-16129644-49176295

CRS7 16129802-16130248-16128912-16129801-16129644-16130476-16129643-16130477-16128396-16130401-90111448-16131524-16130424-16131525-16131890-16131342-16131343-16128321-16131890-16128507-49176239-171701683

CRS8 90111246-16129265-90111481-16130617-16128435-16129702-90111680-90111385-16130036-16131295-16131296

CRS9 16128428-16129755-16129719-16129720-16130879-16130880-16130275-16130879-16130880-145698292-16131364-90111421-16131365

CRS10 94541137-16128281-94541137-16128281-308209620-16129141-16129141-308209620-16129927-94541137-94541137-16128281-16128281

CRS11 90111481-90111481-16130617-16130617-90111481-16130617-16130633-16130632

CRS12 16131200-90111675-16131845-16128451-16128452-16128575-16128576-49176497-226524765-16129861-16129862-90111138-16130965-16130966-16132188-94541134-345452718-345452719-16132112-16132153-16129904-16129905-90111675-16131845-16131284-90111112-16129631-16128290-145698318-16130631-16130010-16130011-16129184-226524726-16128576-16129854-16128765-16132049-162135896-16132048

CRS13 16128192-16128193-16131779-16131677-16131678-16129149-16129150-16128955-16128956-16129516-16129517-16130904-16130906-16131838-16131839

CRS14 90111665-16131021-90111603-90111743-16132021-296044035-90111655-16131202-16131203-16128966-16128967-16129969-16128265-16130084-16132120-16130667-16131958-16131958-16130667-16130844-16129691-16130581-16130580-16128584-16129388-16131970-16132186-16129387-16131200-90111154-16131104-16128602-90111575-16131104-16128515-16129419-16130331-16132082-16132083-16129633-16132048-16132049-90111643-16129139-16128832-16130002-90111154-16131306-16128602-16132055-16132054-16131490-90111622-16131624-49176402-90111225-16128602-16130507-16132166-90111154-16129733-16130506-16131306-16128203-90111361-16129122-16129539-145698243-16128258-16128832-16130357-90111444-16130667-145698244-16130049-16130504-16130505-16131570-16130617-90111481-16128266-16131389-16128266-16131389-16130176-16130175-16129659-16129658-16129978-16130687-16128278-16129736-16130918-94541107-16128040-16128063-16130719-226524697-16128685-16128684-16128914-16128915-16128501-16128502-49176177-16129981-16129630-16129460-16128684-16128807-16128685-90111175-90111444-308209621-16128057-16128058-16130745-16130747-16132041-16132042-16129100-90111458-49176011-16128299-16130531-145698303-90111206-90111332-16128828-16129210-145698250-49176107-16129379-16129380-16130835-16129743-16129851-16128417-16131611-16131612-16128255-90111494-16130749-16128681-16129419-16131356-16128203-90111372-16129959

CRS15 145698229-145698229-16128543-16128543-16128773-94541112-94541112-16129640-16129640-16130093-16132188-94541134-16129661-16128773-16132188-94541134-16128723-16130324-16130325-162135920-16131296-16131295-16132115-16132114-16130668

CRS16 90111444-16131242-16131243-90111562-16131133-16129075-90111214-145698279-16131469-16128443-90111494-16130749-90111206-16128502-16129732-16132089-16128501-16129733-16132090-16132176-90111739-16128770-49176425-

16131680-16128042-16131470-90111561-90111421-145698292-16129854-49176425-226524726-16131680-16131470-90111631-16131535-16129749-16128117-90111088-90111287-90111489-16130682-16129660-16128593-90111681-16131887-16128705-16128706-16131937-90111286-90111690-90111691-16128034-16130703-16130814-16129901-16131279-16131280-90111440-226524741-16130643-16130916-16128156

CRS17 16129460-16129771-16128194-16129244-49176071-16128979-16130473-16129301-16128193-16128192-16130391-16128034-16128567-16128568-90111383-16129771-16130875-16128212-16128213-49176308-16131577-16130304-16129378-16130887-16130997-345452715-16128252-16129324-16128723-16129975-16131104-90111155-90111458-90111144-16130897-16131427-49176373-16131442-16128984-16129256-16129428-16131747-49176442-16129875-16129876-16131420-90111612-16131389-16128386-16132213-16132214-16132133-49176480-49176479-16129557-90111440-16131736-145698342-16129123-90111684-90111081-90111211-16129051-16130744-16130962-16128034-16130026-90111383-16128573-16128574-226524724-16130668-16129017-16129018-16130668-16129017-16129018-16129517-16129516-16128669-145698231-16130801-16131172-16128161-16128162-16128367-16129973-16128570-90111235-16129237-16128723-16132202-90111462-16130503-16129851-16130022-16131049-16131050-226524724-16131740-90111562-16130791-16131133-16128520-16129122-16130043-16130044-16128981-16132115-16132114-90111307-49176127-49176390-16128366-16128367-90111295-16129494-16130684-226524742-16130639-16130640-16128921-16131126-90111705-162135916-16129559-145698340-16128864-16128865-16130462-16131912-16130463-90111079-16129100-16129179-16129180-16131300-16131955-16131299-226524729-16129612-16129613-90111368-16131318-16129301-16128208-90111100-90111685-16128828-16131916-16129253-16130875-16128606-16131034-16128481-16128568-16128567-16129661-16128626-16128625-16131122-90111560-16132049-16132048-145698342-16128586-16130667-16129202-145698318-16129075-16130476-94541126-16131432-90111214-16130477-16132220

CRS18 16129086-16131325-16130103-16128022-16128032-16128209-16128214-16128308-16128308-90111117-345452717-94541100-16128650-16128665-16128701-90111173-16128828-16128984-16129013-16129041-16129695-49176170-16129985-16129985-16129999-16130069-16130082-16130170-90111452-16130719-16130825-16130859-90111523-16130965-16130966-16130978-16131134-16131134-49176343-16131325-16131415-16131471-145698327-90111642-16131593-16131595-16131742-16131755-16131775-16131842-16131842-16131859-16131863-16131880-16131894-16131922-16131933-16131933-16132002-49176475-16132059-16132081-16132145-16128607-308209619-16129799-16130377-16130377-16130976-16129221-90111657-90111131

CRS19 16132062-16128269-49176239-171701683-16131321-16130724-16131570-16130196-16128465-16130262-16130135-16130930-90111528-16130727-16130213-16129715-16129714-16130131-16131034-90111633-16131706-90111257-16128357-16128990-16130927-16132089-16131627-16132090-90111609-16130745-16128662-16128663-16128614-16130519-16128596-16131461-145698339-16131856-16128382-16128740-49176448-49176107-49176330-16131415-16129093-49176311-90111609

CRS20 16129123-16130920-16130921-90111685-16131916-16128134-16128896-162135899-16129658-16129659-16130095-16130096-16130335-16130336-16128364-90111125-16129630-16130272-90111155-16131019-16131020-16129341-16129342-16128547-16130049-16128871-145698325-49176370-16129723-16131059-16128063-226524697-90111180-16131387-16131740-16131740-16131294-345452718-345452719-90111685-16131916-16128809-16128810-16128790-16127995-16129338-16129018-16129017-16129927-16130814-90111685-16131916-16132115-16132114-16128705-16128718-16128706-90111507-90111685-16131916-16130939-16131389-16129308-16129309-16131740-16131316-16130756-16131034-90111144-49176442-16131747-90111238-16129650-16129338-16130107-16130108-16129388-16129387-16129011-16129876-16129010-16129875-16129876-16129390-16129875-90111081-90111144-16131019-16131294-90111155-16131020-16130887-16128844-16128843-16128605-16129390-226524725-90111347-16128258-16128614

CRS21 16128723-16132114-16132115-16130324-16130325-16131216-16131312-16131313-16131312-16131313-16130904-16130906-16132188-94541134-16132115-16132114-145698229-16128543-16131852-16128780-145698229-16128543-16129410-16129411-16129204-90111539-16128723-94541112

CRS22 16129919-16130199-16131653-90111175-16128807-16129846-16128737-145698233-16128924-16131639-16128843-16128844-16129631-16129919-16130703-16131152-16131153-16129863-16131152-16131153-16128694-16128871-16129103-90111238-16129390-226524725-16130096-16130801-16131030-16131740-16128816-16129814-90111347-16130049-16130095-16130964-16131030-90111561-90111603-16131852-90111679-16131873-16131970-16128815-90111158-16130430-16130708-90111158-16128628-16128628-16128815-16128816-16128988-16129100-16129123-16129226-16129227-16129452-16129559-16129561-16130205-90111413-145698292-16130331-16130584-16130671-16130939-16131226-16131227-16131295-16131296-16131314-16132134-16130672-49176377

CRS23 16128576-16129335-16129504-145698229-16128543-16130148-16131312-16131313-16130936-226524747-16128452-16128451-16128723-16129335-16129504-16128575-16128576-16128543-145698229-94541112-94541112-16129640-16129661-16132133-16131318-16130965-16130966-90111383-16130011-16130010-16128590-16128591-16129411-16129410-16128723-16128904-90111190-94541134-16132188-16130608-49176260

CRS24 16129123-90111440-90111144-221800782-16128026-16128604-16130187-145698288-16131455-16131456-16129123-16130780-16131736-90111685-16131916-145698346-16129919-16128481-90111655-16128133-16129676-90111295-49176138-90111728-16131127-16128528

CRS25 16128635-16128871-16131529-90111086-16128156-16131097-90111282-16129441-16129852-16130187-145698288-16130755-16128255-16128311-16129013-16130146-16130887-16130807-16131958-16129121-16129123-16130543-49176368-16131411-16131436-16131437-288551665-16129675-16131524-16131525-16128465-145698233-16128737-16129245-16128481-16128904-90111190-162135899-16129429-16130279-16130280-16128519-16130301-226524735-16128105-

16128106-16128554-16128555-16129165-16129452-90111440-90111495-16131389-16131935-16128778-16129511-16129855-16130341-16130757-16131318-16131736-16131836-226524721-90111429-16130392-345452722-49176263-16131049-16131050-16131050-16129242-16131049-90111726-16128271-16128272-90111214-16129075-90111476-90111372-16129959-16131202-16131203-16129463-16128611-145698252-145698339-16131856-49176170-145698334-16131382-16131383-16128988-16130158-16131238-94541127-16131473-16130768-16130499-16128012-49175991-90111154-16130186-16130429-16128602-16128988-90111407-16130428-16128156-16129411-16129410-16128611-16130430-16130751-16131935-16131458-16131294-16128258-90111238-16130835-16131653-90111690-90111691-16130952-90111184-16130368-16131529-90111121-16129102-16129102-16129102-16129102-16129102-16129102-16129102-16131337-16129101-16129101-16129101-16129101-16129101-16129101-16129101-16130801-16131336-16129295-16130818-16128961-16128962-16130672-16130671-16128740-90111355-16130867-16130168-16130225-16131331-90111193-90111631-16131535-16128604-90111214-16129075-16131524-16131525-90111645-16129452-16129901-16131295-49176479-16131296-49176480-16131627-16129072-16129204-16130684-226524742-16130396-145698299-16128604-16130620-16128022-16128209-16129852-16130580-16130581-16128213-16128675-16129335-16129460-16129504-16130119-90111452-16130900-16131366-16131689-16128212-16128265-94541101-16129459-90111316-16130306-16130901-16131367-16131471-16131688-49176489-16128595-16128302-16128303-16128534-16129020-16129622-16129819-16129855-16130153-16130168-90111476-16128025-16128266-16128527-90111321-90111348-16130512-90111692-49176480-49176479-16128582-16128605-16128843-16129100-16129102-16130176-16130504-16130867-90111084-145698220-16128417-16128469-90111144-16128583-16128844-16129101-16129429-16129447-94541112-16129658-16129755-16129927-16130022-16130175-145698293-90111440-16130505-16130608-49176260-16130747-16131101-16131258-16131331-16131382-16131383-16131498-16131736-90111695-90111702-16132117-49176017-16128468-16128779-16128952-16129093-16129100-16129633-16129659-16130780-16130814-16130939-16131314-16131849-90111703-16128657-90111187-16129079-16129633-16129808-90111389-16130110-16131364-16131736-16132089-16128896-16129078-16129151-16129152-16129500-90111305-16129930-226524732-16130597-16130807-16130978-90111575-16131365-16131488-90111663-16132090-16131339-16131340-90111163-16130659-16130660-90111137-16129020-16129349-16129745-16132130-16129348-16129744-16129855-90111363-16128300-16130187-145698288-16131316-16128026-16128205-16128206-16130745-90111183-16130844-16129041-16129478-90111361-16130780-16130829-49176489-16130272-16131387-90111568-145698229-16128543-94541112-16131767-16128315-16128316-16129017-16129018-90111291-16129493-16131074-16131819-16128373-16131418-16130696-90111286-226524721-90111083-16132220-90111084-16128986-16131852-226524727-16130439-16130738-16129632-16128670-16129003-16129631-16128904-49176133-145698342-90111190-16129620-16130492-90111084-16130825-49176156-16129954-16128602-90111154-16128635-16129444-16129003-90111379-16130007-16130240-16131104-16131202-16131873-226524703-16131203-90111679-16129076-16128920-16129167-16129168-16132060-16132134-16129973-90111641-16129304-145698281-16130152-90111476-16131104-16131495-16131736-226524713-16132177-90111155-16128659-157783150-90111611-16131903-16131912-16130129-16130230-16130130-16130231-16128639-16128675-90111214-16129217-16129419-16129495-16129501-16129624-90111383-16130272-16130296-16130965-162135908-16131597-16128106-16128650-16128674-94541101-16128871-16129075-16129216-16129496-16129804-16129946-16130297-16130966-16130974-16131011-16131155-16128134-16129020-49176377-16128073-16128154-16128203-16128657-16129179-16129339-94541111-16129612-16129613-16129759-345452722-16130780-16131436-16131437-16131825-16131934-16131935-16132062-16129180-16130275-49176263-226524759-49176450-16128543-16129075-16129488-90111361-16131152-16131308-49176377-16132186-16132202-16128164-16128540-145698229-16128662-16128663-16128843-16128966-16128967-90111214-16129123-16129165-16129294-90111286-90111289-90111310-16129601-49176170-16129969-16130204-145698297-16130430-16130751-16130988-16131104-16131202-16131203-90111581-16131274-16131275-16131307-16131498-90111645-16131609-90111651-16131718-16131755-16131775-90111680-16128694-16132040-16128755-16130859-16128264-16128468-16128481-16128602-16129123-16129123-16129622-226524721-16130275-16130275-16130504-16130608-49176260-16131411-16131794-16128469-90111154-16130049-16130505-16128448-16128682-90111492-16131626-16128425-16128447-16130084-16130620-16130722-145698331-145698220-90111122-16131710-16128148-16128715-16129348-16129349-16130707-16130708-16128147-226524711-16128007-16128069-16128203-16128462-16128796-16129345-90111264-90111286-16129562-16129595-16130144-16130158-16130703-145698336-90111663-49176474-16128008-16128795-16129100-16130230-16129342-16131238-16129341-221800779-90111085-16128203-16129100-16129683-16130049-16130956-16131049-16131050-16131217-16131380-16131415-16130957-16131504-49176415-145698340-16129341-16129342-226524713-16131411-145698312-16130801-90111364-308209621-90111410-16131959-49176343-49176361-90111244-90111245-16130093-16131217-90111186-49176377-16131970-90111147-16128527-145698231-16129146-226524731-16130591-16130594-16130602-16130967-145698310-90111643-49176479-49176480-16128513-90111147-16128515-16128638-16128669-162135899-16129211-16129293-16129676-49176138-16130078-16130079-16131843-90111701-16132012-16128239-16128604-16129630-16129736-16130551-16130696-16131536-16132134-16129737-226524715-90111388-16130052-16131104-16130187-145698288-16129675-16128904-90111190-16131019-16131020-16129702-16131340-16131339-16128630-90111277-16129672-16128631-16129413-16130887-16128591-16128636-16129076-16129472-16130292-16130306-94541127-16131473-16131892-49176479-16128417-16128481-16128590-16128822-16129471-16131063-90111603-90111684-49176480-16129570-49176125-16128721-16129017-16129018-90111363-16128722-90111730-16132186-16128007-16128012-49175991-16128502-16128906-16129020-90111209-16129101-16129102-16129123-90111238-16129294-16129338-16129572-16129616-16129676-16129806-16130272-16130608-49176260-16131076-90111557-49176329-90111609-16131892-16128008-16128501-90111155-16129029-145698270-16129650-49176138-16129805-16129814-90111363-16129975-16130113-16130745-16130887-49176302-16131034-16131077-16131117-16131387-49176426-16128239-16128602-16128952-16129159-

16129428-16129804-226524726-90111573-16131279-16131399-90111655-16128373-16128527-16128537-16128604-16128755-16128870-16129065-49176077-16129660-90111370-16131069-16131070-145698340-90111084-90111154-16128665-16128773-16129854-90111440-16131780-90111741-16129845-16129846-16130963-145698342
CRS26 16131970-16131295-16131296-90111214-16129075-226524727-90111083-145698299-16130396-90111655-16129216-16129217-16128502-16128501-16131314-16130745-49176479-49176480-16130324-16130325-90111183-16131148-16130428-16131377-16130429-16129691-16130272-16129826-16131852-16128502-16128501-16129429-16130608-49176260-16129294-16130885-16130886-16128904-16128904-90111190-90111190-16131019-16131020-16128778-90111631-16131535-16129672-16130147-16131747-49176442-16132186-16129075-90111214-16128375-16128970-16128694-49176343-16132133-16129294-162135907-90111457-90111476-90111154-16128602-16130331-16131377-16128604-16131502-16128871-90111123-16128843-16128844-16129338-16130504-16130505-16128156-49176356-16129416-16129460-16130814-16132214-16132213-16130368-16129341-16129342-16131152-16131153-16128272-16130755-16128271-16131653-16128519-16128491-16131570-16128695-16128430-90111136-16129447-162135899-16129495-16129496-16130887-90111440-16131736-16130187-145698288-16129245-16129846-16129847-16131843-226524735-16128069-16128012-49175991-16128013-16128255-16130689-90111617-16129101-16128106-16128311-90111372-16131202-145698342-16129959-16131203-16130844-16128605-16128109-16128681-16129419-16131356-16129612-16129613-16129020-145698256-16129511-90111282-16129441-90111295-145698256-90111597-90111596-90111384-16130939-145698293-16128417-49176293-16131331-16128244-90111105-16128777-16128765-162135896-16129339-94541111-16130887-16131247-16131258-49176138-16129676-16129901-16131200-16130168-16128604-16129342-16129341-16132220-16128025-16131779-16129495-16129496-16132177-16129778-49176463-145698243-145698244-16131119-16130745-145698249-16131247-16131259-49176133-16130146-90111459-16129620-90111460-16128279-16132003-16129847-16130292-16129783-16131794-16131034-16131202-16130272-16131203-16129601-90111310-16128273-16128548-16131611-16131612-49176350-16128311-90111417-16131360-90111718-16128034-16129511-16128586-16128386-90111383-16131034-90111355-16130867-16131097-16130301-16131364-16131365-16128396-16130580-16130168-16130581-16129416-16128303-16128302-90111083-90111103-49176249-90111083-162135899-16131529-16129165-16130430-16130751-16131247-16131529-16131104-16131034-16132063-308209623-90111144-145698243-145698244-16129650-16129927-226524735-90111645-90111282-16129441-16130306-16130286-16128134-16129927-16128676-16129676-49176138-16131456-16131455-49176011-16128299-16128676-90111186-16130887-16128528-16131852-16128611-16128547-16130430-16130974-16129078-16129079-16130990-16132021-296044035-16129123-16131377-16127995-16130272-90111193-16128789-90111238-16128952-16130175-16130176-16129010-16131388-16129011-90111286-16131330-16131331-16129562-16129236-16131318-16131873-90111679-16128904-90111190-16131020-16128966-16128967-16129697-16128481-16131949-16130887-16129211-16129714-16129715-16131247-16131382-16131383-16128311-16130715-16128462-90111123-16129244-16131935-16129675-16131508-16132013-16129139-16129140-90111741-145698340-16129495-16129496-16130461-16128212-16128213-90111665-16131798-16130939-16129013-16128623-16129127-16128654-16128655-16129126-16129284
CRS27 16130320-16130321-16130089-145698297
CRS28 16131779-16131776-16131777-16128244-90111105-16131776-16131777-16128582-16128583-16129186
CRS29 16131152-16131153-16131152-16131153-16131318-90111603-16131740-16129308-16129309-90111603-90111603-16129612-16129613-90111416-90111389-16131318-16131247-16130445-90111451-226524734-90111622-16130208-16131490
CRS30 90111084-145698223-16128332-16128437-49176025-16128481-16128801-16128955-16128956-16128980-16128981-16129004-16130205-90111413-226524762-16128548-90111155-16129557-90111431-90111440-90111458-16131038-16131039-16131539-16131736-145698342-16131935-16127995-16128378-90111128-16128694-308209619-145698274-226524729-16128855-90111274-16129557-16131843-16131903-145698258-16129623-16129831-145698229-16128543-90111154-16128602-16129101-16130306-16130543-16131104-16131152-16131153-16131202-16131203-16131604-90111679-16131873
CRS31 49176017-16128747-16131596-16128554-16128555-16131942-16130172-16129565-145698268-226524762-16132153-16131596-16128855-49176416-16128101-16128383-16128384-94541127-16131473-16128552-16130807-16131391-345452718-345452719-16131306-16130807-16128443-90111387-145698243-145698244-16129089-16129090-49176474-16128675-16129463-16129930-16130251-16128674-94541101-16130738-226524729-16131019-16131020-16131098-16129238-16129239-16131846-90111474-145698223-16128332-16128462-16131399-16128931-90111196-90111384-90111476-226524747-16130936-16131367-16128843-16128844-16129247-16131766-16128723-16130187-145698288-16130755-16131934-16131935-16130022-90111100-16130630-16128208-16129971-16131882-16128654-16128655-16129295-16129732-16129733-16130828-145698322-16128986-94541110-16131895-90111149-16128542-16130987-16128239-16129521-16128138-16130871-16130428-16130429-16131263-16131890-16130492-16129466-16128588-16131950-90111151-16128057-16128058-16130546-16132062-16129879-16130987-16130620-16129770-16129771-90111362-16129916-16129522-16128864-16128865-16128459-90111384-16131486-90111106-16128581-16130103-16132133-16128109-90111362-16129926-16131469-16128034-16129916-16131470-90111135-90111134-16128828-90111519-16131325-16127999-16128554-16128555-90111171-162135897-90111175-16128807-90111250-49176392-49176393-16131722-90111659-16131220-16129301-90111330-16130626-49176074-16128676-16132098-16128828-16129723-16131653-16129416-16131377-16129308-16130492-16131620-16130924-16130923-16130544-16128920-16130871-226524747-16130936-90111730-16128329-16129613-16129612-90111316-90111552-94541127-16129184-49176207-16131473-16130257-16128008-16128189-16129398-16128007-16129295-16130244-16130882-226524702-16131895-16131896-16128790-16129494-16128547-16128761-16130645-16128556-16129104-90111583-162135911-16130898-16129073-16132135-16130231-16130428-16130429-16131294-

16127995-16128212-16128213-16128694-16129452-90111492-16130722-16132030-90111519-16131416-16130504-16130505-49176441-16130219-16131495-16129072-16129557-162135896-16128563-16128765-16129124-16130723-16128233-16128584-16130696-162135914-90111624-16131860-16131861
CRS32 16128980-16131765-16131766-145698229-16128543-16129338-16129630-16130587-16129140-16131274-16131275-90111153-16128915-16128914-16129615-16131216-16131217-16129852-90111631-16131535-90111497-16132003-16128747-16129458-90111316-16131794-16131238-16128822-90111407-16130186-16130771-49176302-16132188-94541134-90111106-90111316-16129180-16130275-226524759-16129179-16130176-16130175-16128311-16128871-16129020-16130522-90111465-16129335-16129504-49176329-16131117-16131217-16129858-90111454-16131427-16130962-226524724-16130894-145698281-145698288-90111083-16130187-90111497-16131217-16128241-16131356-16129863-162135908-16131019-16131011-16128547-90111103-16128604-16129929-90111655-145698346-90111555-16129759-16131023-16131024-16129927-16129781-90111339-16131596-345452722-49176263-16130780-16128096-16128097-16129861-16129160-16131942-16131259-16128543-145698229-94541112-16130914-16130887-16131279-16129558-226524758-16131010-16131549-49176463-16130175-16130176-16129447-16128731-16128474-49176140-16130945-16128459-16128473-16129294-145698276-16130946
CRS33 16131884-16131885-16129139-16129140
CRS34 16131470-16128655-16128654-16128443-16131469-226524726-16129854-90111739-16132176-90111494-16130749-16132089-16132090-16131279-16131280-145698279-16130518-90111206-16128034-16129854-90111739-226524726-16131470-16132176-16130814-90111489-16130682-226524699-49176425-16131680-90111544-90111088-16128117-16131937-226524713-16130780-16131469
CRS35 16129988-16130633-16131852-16130632-90111276-16130967-145698310-16129885-16129884-16131105-16130225-16131155-16131653-16130168-16128203-90111286-16128845-90111182-90111331-145698243-145698244-16131970-16132195
CRS36 49176308-16129854-226524726-16129863-16131470-16131740-16129365-145698224-145698254-145698282-145698306-145698309-145698344-145698342-16128896-16129310-90111642-16131202-16131203-16130708-90111137-16129360-226524741-16130643-16130428-16132219-16130429-16132218-16129146-162135899-90111326-16129633-90111568-16131030-16130591-16131379-16132200-16128501-16128502-16129933-16129284-16129919-16129452-16129852-16128610-90111557-16130988-16129301-16130987-94541130-16130698-49176425-16131680-16132002-16131104-16129166-16131837-90111743-16129123-16131101-16131680-49176425-16131126-16130042-16130069-90111743-16128747-16129253-16128264-16130341-16128659-157783150-16128366-226524726-16129854-16130587-16130939-16128706-16128749-16128705-16128748-16128657-16130492-16128489-16129971-16128488-16128520-16131049-16131050-16129854-226524726-90111679-16131873
CRS37 16131028-16129575-16128633-16131023-16131024-49176401-226524732-16128809-16128810-16131023-16131024-16131028-16129723-16129363-16129937-16130763-16128417-16131024-49176398-16131023-16131806-16128543-16128543-16128623-16129805-16129806-16130273-16130273
CRS38 16129238-16129239-90111167-90111643-16130094-16128639-145698299-16129198-16129482-16131320-16131319-16131488-145698262-90111495-16128843-16129774-16129294-16130396-16131279-16131280-16130552-16130620-145698346-16130319-16128203-162135894-16131508-90111145-16127999-16128295-16130604-16132054-16132055-16128721-16128722-16130042-16128107-16129226-16129227-16128502-16128501-16130591-16130977-145698321-16131653-90111718-90111131-16130022-16128560-16131392-226524713-90111484-16131337-16131336-16129087-16129520-16131700-16128143-16128871-16128481-16129572-90111718-16128053-16128270-16130739-90111695-90111391-226524746-16130088-16128639
CRS39 16131325-16128555-16128554-16128984-16128849-16128848
CRS40 16128022-16128032-16128209-16128214-94541100-16128650-16128665-90111173-16129013-16129041-49176170-16129999-90111384-16130069-16130825-16130859-90111523-16131134-16131134-16131325-16131415-90111642-16131595-16131755-16131775-16131782-16131842-16131842-16131863-16131933-16131933-16132145-16128308-16128308-90111117-345452717-16128701-16128828-16129985-16129985-16130082-16130170-90111452-16130719-16130978-49176343-16131593-16131742-16131742-16131742-16131859-16131880-16131894-16131922-16132002-16132059-16132081-49176045-16130257-16129642-16129252-16128878-49176107-49176330-16131415-16129093-90111609-16128055-16128055-16128055-16131324-145698339-16131856-49176368-16131411-16130376-162135895-16128668-90111384-16130170-16131059-16131970
CRS41 16129345-90111264-16128706-16128705-16128705-16128706-16128488-16128489-16129847-16128367-16129846-16130368-16129652-16128109-16129653-16131104-16131203-16131202
CRS42 16128034-90111316-90111081-16128264-16128582-16128583-16130608-49176260-16129444-16130743-16130742-16128134-16128904-90111190-90111209-16129029-90111603-16130807-16128865-16128864-49176302-16131653-16128586-16129676-49176138-16129826-16130119-16130430-16130751-90111713-226524724-16128547-90111158-16128628-16130095-16130096-16130974-16132133-16129721-16131316-16128984-345452721-16129076-16132134-16128570-16129378-16128116-90111087-16128375-16128528-16129455-16130939-16130885-16130886-16129675-16131994-16128540-145698256-90111310-16129601-49176329-16131117-16128106-90111435-16130360-16129663-16131815-90111684-16130771-16131115-16128832-16130152-90111167-16131588-16128502-16129511-145698342-49176017-16128501-16131314-16128212-16128213-16129165-16130430-16130751-16131740-16129845-16129846-16131736-16128109-16131498-16128212-16128213-16129348-16129349-90111378-16130756-16128291-16128650-90111603-16128989-16131949-16131531-90111286-94541128-16129702-16129715-16131247-16131529-16129714-16130272-16129185-16131794-16129186-16129294-

16129545-16128989-49176414-16130734-16129123-16131437-16130814-16131436-16129517-16129516-16129847-16131757-16128659-157783150-16128154-16129092-90111410-16131152-16131153-16131913-16130887-16129891-16130898-16131387-16129851-16129165-90111655-90111287-16129338-16130152-90111186-16130887-16128676-16130755-90111552-90111726-90111081-16130617-90111481-16130898-16128703-16130396-145698346-16131262-90111155-16128386-16129545-16131152-16131846-145698340-16132049-16131153-16132048-16132177-90111631-16131535-16130306-226524749-16130147-90111112-16128290-226524743-90111666-145698224-16129242-16129676-49176138-226524721-16130010-16130011-16130272-145698309-345452721-226524753-90111196-16128960-16128931-90111200-90111732-90111731-16130208-226524734-16131247-16129631-90111372-16129959-16131755-16131903-16130251-16131503-16131504-16128165-16128527-16130002-226524734-16131034-16131034-16131389-16131958-145698263-16130208-16129691-16130299-16131247-16130236-16130237-90111122-16128310-49176329-16131117-16130844-16131034-16129572-16132189-16132190-16130885-16130886-90111631-16131535

CRS43 16128723-145698229-16128543-16128723-16128543-145698229-16128573-16128574-16131216-16129204-16131318-16129640-16129020-16130967-145698310-94541112-16128780-94541112-16131019-16131020-345452718-345452719-171701682-16129786-16129661-90111238-16129084-16132115-16132114-16131313-16131312-16129630-16128547-16132133-16128203-16132188-94541134-16132133-16130186-90111407-16128114-16132114-16132115

CRS44 16128133-90111631-16131535-16128117-90111088-16129338-16130918-16130703-16130734-171701682-16129786-16129119-16129851-16128258-16129471-16129472-16131395-16128810-16128809-49176474-16130918-16129123-16130999-90111387-16129152-16128657-16131934-16131935-16128527-145698325-49176370-16131490-90111622-16129494-90111158-16128628-16128417-90111316-90111617-16130583-16131086-16131085-145698224-49176343-16129407-226524714-16129751-226524724-16129455-16131052-16131258-226524721-16128755-16130703-16129214-16130168-16129801-16129802-16130912-90111458-16132030-90111509-16129324-16128770-16131780-345452718-345452719-90111083-16129340-16128904-90111190-16128527-16131692-16131693-16131766-16131765-16130306-16130976-16128871-16129875-16129876-90111685-16131916-16131019-16131020-16132177-16128832-16129452-16130275-16128576-16129539-16128563-16128681-16131356-16130956-16130957-16131596-90111310-16129601-16129245-16131053-16128311-90111185-16128873-16132188-94541134-16130939-16129516-16129517-90111690-90111691-16129855-16128258-16130780-16129583-90111489-16130682-16131360-16129441-16128780-16129978-16128952-16130172-90111675-16131845-16128669-145698231-16128765-16132049-162135896-16132048-16128255-16128674-94541101-16128675-16128896-16128770-16129530-94541112-16128720-16131316-16128575-16128576-16130129-16130130-16131998-90111196-16128931-221800781-16129289-16132134-90111679-16131873-16130168-16130597-16128501-16128502-16130875-226524756-16131700-16129301-145698340-16128723-16128605-16131314-16130887-16131555-90111638-90111652-16131671-90111158-16128628-16128921-16131718-226524731-90111149-16128542-49176074-16130844-16129969-16132003-16130608-49176260-16131314-16132112-16129020-16128134-16131840-16130175-16130176-16130939-16128009-16128481-16130208-226524734-16130584-16130272-16128606-16129520-16131021-90111083-90111503-16130768-49176377-16129165-145698255-16129373-145698229-16128543-49176207-16128879-145698248-16129182-16128227-16128228-16130096-16130095-16130498-16130499-16128828-90111728-226524739-16129100-16129373-145698255-16131740-16131247-16131529-16131955-16128000-49176407-145698249-16132186-16130984-90111562-16131133-16130684-226524742-16131266-16128628-90111158-16128718-16129079-49176074-16131455-16129078-16131456-16131849-16131247-16128547-16131736-90111575-16128013-16129466-16131020-16131529-90111291-16129493-16128952-16131531-94541128-16130172-90111137-16131890-16128785-16130158-16131934-90111171-162135897-16129216-16129217-16130771-16129284-16131458-90111384-16128477-90111679-16131873-16130643-226524741-90111158-16128628-49176302-90111103-90111387-16131624-49176402-16131284-49176329-16131117-90111288-16129485-90111389-16129972-16129561-16132161-16132162-16131024-16131023-49176361-16129848-16129493-90111291-16129004-145698299-16130396-16128195-16128196-16130745-226524753-16130755-16131364-16131365-16129146-162135899-90111268-145698309-16130990-90111166-16129160-16131217-16130747-90111137-16130208-226524734-16128366-16128367-16128845-90111182-16129901-16131698-16131699-16129373-145698255-49176171-16131126-16131127-16130608-49176260-16132054-16132055-16129428-16129970-16128228-16128227-16131028-16129954-90111634-16129338-49176074-145698341-16131881-16131888-16131889-16132048-16129395-94541092-16130885-16130886-16131967-16131966-16129733-16129732-16130939-90111155-16128134-16129324-16128026-16129513-16131030-16129020-16130920-90111685-16130921-16131916-90111407-16130186-16131377-16131937-16132041-16132042-16129575-145698252-16131848-16131847-16130557-16131852-16131747-49176442-16130583-90111316-16129326-16129339-94541111-49176407-16130577-16130974-90111655-16131794-90111705-162135916-90111084-16131755-16130292-226524759-16131217-16128013-16128527-16128955-16128956-90111295-90111144-16131284-16131815-16129410-16129411-16131284-16130584-16131395-16128138-16128462-16128459-16131885-16131884-16130311-16130312-16129960-16129460-16130844-16128548-16128980-16130272-90111081-16128034-16131794-16129185-90111378-16128757-90111627-16131399-16128844-16128843-16128111-16128110-16128843-16128844-16131331-16130584-90111181-16131152-16131153-16131504-16131503-16130331-90111434-16129676-49176138-16129100-16129255-16131217-90111344-162135900-90111234-16131407-16131408-16131840-16128984-16128548-16128034-90111083-16130084-16130771-16128570-90111718-16131318-16131958-16128013-16128925-16128832-16128926-16129650-16129065-49176077-16130512-16128605-16128931-90111196-16128886-90111629-49176286-90111514-16129204-16128754-16131202-16131203-90111655-16131824-16131722-90111659-16131337-16131336-16130350-90111304-16129930-16131217-16131639-16131347-16128582-16128583-16130381-16129459-90111112-16128290-16128604-16131871-345452722-49176263-16128871-16128925-16128926-16129123-16129613-16129612-90111634-16130333-16130334-16130311-16130312-16131653-16128594-226524762-16129179-16129180-16131782-16129429-16131333-

16130368-49176329-16131117-16132134-16131023-16131024-145698220-16129308-16128424-16130441-16130823-
145698331-16131626-16131356-16130793-16129545-16130513-16130428-16130429-16127995-1612971-16129646-
16130320-16130321-16128989-90111293-16129525-90111112-16128290-16131197-16131504-16128465-16129541-16129540-
16130583-16131104-16128373-16130400-49176011-16128299-16131385-90111320-16131242-16131243-90111688-
162135908-16130999-16131932-90111379-16130007-16128366-16128367-16131852-90111180-16131387-90111440-
16131039-16131038-16131740-90111165-90111294-16128045-16129697-16128986-90111415-90111440-90111361-16128026-
16129658-16129659-16130095-16130096-16129338-16129569-16129338-90111645-49176177-16128684-16128685-90111361-
16128273-49176267-90111293-16129598-49176129-16130825-16132048-16132049-16129949-226524721-94541109-
90111114-16129335-16129504-16129556-16131331-16129119-16129852-16130835-16129804-16131217-16129059-16129619-
49176035-16128564-16128694-90111184-90111643-16128755-16130147-16129554-16128681-16129553-16130755-16131259-
16129231-16129658-16129659-16130505-16130504-16130371-16129794-16129795-90111622-16131490-90111702-90111703-
226524761-16130360-90111435-16129702-16130272-16129335-16129504-16131284-49176071-16128979-145698345-
16129100-16129160-16128611-16131049-16131050-16130882-16130924-16130923-16130368-16128026-49176156-16129578-
16129579-16128938
CRS45 16129073-90111430-16129253-16130456-16131328-16130998-16130488-16131988-16129482-16128151-90111609-
16129073-16129799-16130377-16130377-16130976-16130976-16131910-16130882-16128327-16132081-16128321-16128703-
16129013-16130248-16130456-16130577-16131134-16131134-16131859-16128724-16130056-16129184-16130377-16130377-
16129799-16130976-49176441-16128269-16128511-16131323-16131792-16129919-16131545-16129196-90111382-90111555-
288551665-16129864-94541133-16128610-16129700-16129636-49176295-16131513-16129848-16130246-16128614-
90111177-49176448-16131530-16129249-49176448-16128757-16128328-16130543-16131823-16128327-16128209-16128703-
16129832-16130248-16130401-16130456-16130456-16130577-16131134-16129221-16128636-90111657-16128879-16130103-
16128425-16128896-49176391-90111539-16129073-16131526-16130558-90111268-16128654-16128655-16128156-16129913-
16130444-16131837-16128275-16128478-16128879-16131271
CRS46 16131888-16131889-16130858-16128920-16131889-16131888-16131627-345452716-16130518-16130686-16131813-
16129658-16129659-145698236-16130915-16130978-16129954-16129146-90111679-16131873-16128074-16130340-
16128279-90111244-90111245-16130930-90111528-16129390-16130713-90111702-90111703-90111184-90111375-16130357-
49176459-90111242-16130042-16130720-145698226-90111238-16130172-16130631-16132113-16128061
CRS47 16128022-16128308-90111117-90111117-16130619-16130978-16128032-16128214-16128981-16130069-16131134-
16131134-16132080-16131806-16128635-16131919-16131920-16129525-16128208-16128734-90111100-16128735-16131542-
16128553-162135895-16129010-90111637-16130506-16130507-90111559-16128826-16131461-16128902-16131975-
16130927-16131893-90111257-16128269-16130626-16131263-16131927-49176409-16130257-145698339-16131856-
16128506-16128269-16131078-16128951-16131656-49176170-16131732-16129899-90111359-16128244-90111105-16131111-
16131483-16131482-49176390-16128519-16128414-16129252-16131833-90111674-16129700-145698230-16130651-
16130892-16129695-16130966-16131709-16131925-16128984-16130915-16130965-16130976-16131323-16131471-49176475-
16128567-16128965-16128568-16130347-16128914-16128915-16128607-16129831-16128671-16131833-90111674-16130712-
16128965-145698295-90111437-16128032-16130859-16131933-16131933-16131933-16128308-345452717-16131859-
16129425-90111159-16131978-16131438-90111665-16129073-90111180-16131609-16130927-16132105-16131066-90111097-
16131270-16129505-90111332-16129743-16128828-16130495-49176282-16131137-16130978-16130377-90111105-16128343-
16128668-16130271-16132169-16128244-16131763-16128607-16130226-16130619-16130984-16128701-16128804-16128828-
90111198-16129919-16129990-16130082-16130976-16131880-16131922-16128022-16128055-16128055-16128055-16128209-
16128308-345452717-94541100-16128650-16128665-49176045-90111173-16128803-145698236-16129013-16129041-
16129442-49176170-16129985-16129985-16129999-90111384-16130170-16130377-16130377-16130377-90111452-16130456-
16130577-16130719-16130825-16130859-90111519-90111520-90111523-16130976-16130978-16131134-49176343-16131324-
16131325-16131415-90111642-16131593-16131595-16131742-16131742-16131742-16131755-16131775-16131782-16131792-
16131842-16131842-145698339-16131856-16131859-16131863-16131894-16131933-16131933-16132002-16132059-
16132081-16132145-16132199-16131928-16130782-16129424-94541116-90111198-90111657-16129234-16129235-16131927-
16130651-90111176-16130426-16128610-90111637-16131919-16131078-90111131-16131281-90111238-49176475-16129424-
16130798-90111103-16128260-16128668-90111203-94541110-16129762-90111336-16131274-16131275-16131598-16131627-
16131863-90111683-16131058-16132113-16129224-16128663-16129665-16128662-16129665-145698309-145698267-
16130036-90111385-16132070-16130219-16131920-16128588-16130976-90111151-16130377-16130134-16129558-16129224-
16130383-16130506-16130507-145698221-16128950-145698344-90111255-16130738-16129280-90111256-226524729-
226524730-90111176-90111381-16131810-16128769-16129655-16128491-16128568-16128817-16129721-16130894-
16128567-16128818-90111493
CRS48 16128576-145698229-16128543-94541134-16132188-16128576-16129020-16128576
CRS49 16129020-16129338-90111280-90111389-16128417-90111214-16129075-16128547-16128723-16130965-16130966-
16132188-94541134-16131765-16131766-16131503-16128396-16131504-16129807-16131279-16131280-16131312-16128258-
16131313-16130742-16130743-16128553-145698229-16128543-16128723-16129700-145698279-16128143-16131020-
16131313-16131019-16131312-16128574-16128573-16130745-16128452-16129204-16130130-90111675-16128451-16130129-
16131845-16131316-16131104-16131653-90111173-90111509-16128681-16131356-145698258-90111274-16132186-
16128573-16128574-16129857-16129856-16131903-16129373-145698255-16130504-16130505-16128192-16128193-
16128848-16128849-90111277-16129413-90111684-16130324-16130325-16128904-90111190-16129219-49176249-16129452-
90111316-145698306-145698333-49176463

CRS50 90111685-16131916-16128106-16128105-16130715-16130965-16130966-16130049-16128871-16128547-16129123-
90111330-90111244-90111245-226524711-16131740-16131104-16129870-16129871-16130920-16130921-90111685-
16131916-90111137-16131389-16130147-90111603-16130939-16130443-16129862-16129861-90111147-16128513-16130882-
16128779-16131388-226524724-16131970-90111155-16129018-90111489-16129017-16130682-90111507-16129387-
16129388-16129927-16130745-16131881-145698341-16132083-16132082-16128091-16128258-16132076-16132077-
16131165-16130119-16131059-90111645-16128134-16130095-16130096-16130422-16129338-16131389-16128473-16128474-
16129216-16129217-16129461-16129230-16129631-16129311-16131316-16130324-16130325-162135899-16129146-
16128134-16132195-16130168-16128291-16130129-16130130-90111154-16128602-16130396-145698299-162135899-
16130430-16131653-90111576-90111389-90111634-16131455-16131456-16131053-90111122-16128779-90111158-16128628-
16131243-16131242-16129335-16129504-16129185-16129186-16128105-16131469-16131470-16132030-16129072-49176358-
16130100-49176193-16128828-16131916-90111685-90111421-145698292-16128822

CRS51 16129236-90111583-162135911-16131591-16131736-16130737-90111741-16131387-16132133-16129217

CRS52 16132114-16132115-16130324-16130325-16128162-16128161-16130043-16128723-16130044-94541090-16128970-
16128576-16129640-94541112-90111461-16130501-16130308-16130307-16128731-16131377-90111591-16129165-16129165-
16129904-16129905-16128925-16128926-16128548-90111267-90111454-16131952-16131951-16130093-16129151-16129152-
16129204-16131019-16131020-94541112-16129640-49176442-16128301-16131747-90111615-16131312-16128143-16131313-
145698229-16128543-16128543-145698229-16128681-16128754-16129419-90111407-16131356-16130186-16130577-
16130010-16130011-16130106-16128459-16130594-16131852-16131063-16132186-16130780-16131934-16131935-16130682-
90111489-16130957-16130956-145698312-16128602-90111123-16129630-16130640-16130639-16130703-16130272-
16128291-145698344-16131740-16132183-16129130-90111657-16130013-16131488-16131939-90111605-90111606-
16129196-16128258-16131760-16131873-90111679-49176434-16131710-16128834-16128833-16131920-16131166-49176336-
16131247-16130981-16131244-16132188-94541134-16131808-16131881-16128429-16132123-16128549-16128773-90111252-
16129300-16131101-16132194-16129375-16130977-16128773-90111627-16129636-16130430-145698316-90111603-
16128313-16128314-16128904-90111378-16129281-16129282-16130100-49176193-16132186-16128571-16129199-16129411-
16131474-16128437-49176025-16129410-16131954-16129901-16128681-16132112-16131316-16128789-49176392-49176393-
16129637-16132158-16130325-16131312-16128143-16130324-16131313-90111539-16131023-16131024-16130966-16130965-
16131760-16128003-16131718-90111214-16130742-16129075-16130743-16129202-16131941-16129338-16129575-16130002-
16132082-16131104-16131756-16132083-16129124-16131808-16130897-16132188-94541134-16129969-16130130-16129901-
16130050-90111735-16131395-90111361-90111261-16129331-90111286-90111695-16131227-16131226-16130084-16128981-
226524721-16128773-16129244-16128723-16129084-16129460-16130092-16131365-16130091-16131364-16129980-
16130604-16129410-16129411-16128007-16128008-49176408-226524726-16129854-49176489-16131949-16130148-
16132135-16128832-16129978-16130084-16129007-90111316

CRS53 90111214-16129075-16130732-16131806-16129433-145698260-16128715-16129125-90111276-16131552-16129730-
16131076-16131077-16128570-16128581-16130956-16130957-16130299-145698334-16131240-16128681-16129312-
16129419-16131356-16130966-16130965-16131609-16131266-16129562-16129831-16130923-16130924-16129211-16129387-
16129388-16129631-145698297-90111494-16130749-90111602-145698279-226524751-16130524-16130002-16128279-
16129237-16128875-16130230-16130231-226524720-16130602-16128844-16128843-90111125-16129630-16128364-
16130999-16131934-16131935-16129875-16129876-16128778-16129982-16128551-16129488-90111289-90111286-94541101-
16128675-16130388-16130389-16130010-16130011-16128167-16131852-16132158-16130056-16130473-16131382-16131383-
16128343-16128004-16131244-16128386-16128695-16131958-16131793-16128257-16128213-16128212-16128073-16128674-
94541101-16128675-16131722-90111659-16128878-90111476-16131046-16131047-16131104-16130084-16128163-16128279-
16130272-16129870-16129871-16130939-90111121-90111144-226524702-221800782-16129861-16129862-16131105-
16130583-16129054-16130445-90111451-16128548-16128828-226524737-16128445-145698339-16131856-90111627-
16129553-16129554-226524721-16130744-16128012-49175991-16128952-16131030-16131740-90111728-16130885-
16130886-16131389-16131837-90111237-16129213-90111552-49176358-16131126-16131127-16131524-16131525-16131202-
16131203-16128718-16130091-16130092-16128734-16128735-16131153-90111494-16130749-16132123-16129973-16130422-
16129494-16131795-16131796-16131011-162135908-16128124-16128125-16130771-49176276-16129004-16130187-
145698288-90111676-145698279-16128161-16128162-226524762-90111646-49176400-16129576-90111196-16128931-
90111587-16131282-90111331-16128570-90111513-16131325-16128252-345452715-90111509-90111510-90111684-
16130977-16130514-16129977-90111711-16129698-16129552-16129077-16129008-16131458-16130825-16130439-16131223-
16130756-16129583-16131706-16132041-16132042-16128212-16128213-16129022-16131486-16130843-16131094-16128654-
16132202-16131316-90111305-90111484-90111182-16128845-226524720-16130304-16129952-162135904-16129861-
16129862-16131949-90111214-16129075-90111225-16128300-16128779-16128034-90111081-16132040-16128105-16128106-
16131892-16131399-16131890-16132133-16130069-16129086-16131736-16129064-90111438-16129552-16129898-16128526-
16131486-16129804-16129914-90111575-16130584-16130747-90111643-16128547-16131852-16128013-226524713-
16129903-145698309-16131970-16129511-16128809-16128810-16128400-16130597-16128893-145698350-16129926-
16131034-16129124-16128302-16128303-90111244-90111245-90111383-16130026-16130707-16128290-16129367-90111112-
90111631-16131535-16131388-226524732-49176293-16130999-16128668-145698228-16128462-16131488-16130603-
90111205-16129732-16129733-16129078-16129079-16128034-90111081-16131879-16131860-16131861-16130745-
226524703-90111561-16128142-16129981-16130418-16130419-16129125-49176489-49176377-16130667-16130546-
90111724-16128547-16129691-49176401-221800782-16130168-90111403-16132176-90111739-16129969-16130825-
16130324-16130325-16131063-90111474-16130707-16128724-16129904-16129905-16129301-16131364-16131365-16129130-

16128328-145698336-16131718-16131780-16131793-16129057-16128194-16128069-16128937-16131503-16129467-16130667-16129944-16130651-49176368-16131411-16128203-49176025-16132063-16128437-16130299-308209623-16132196-16130755-16128138-16130584-16129187-16129980-16128012-49175991-16129166-16129378-90111307-16131891-49176127-16130875-296044035-16129977-16132021-90111492-16130722-16129100-16129227-16129226-16128300-16129165-16131122-90111560-145698312-16131958-16130183-16131333-90111282-16129441-16128694-16129065-16131023-16131024-16132133-49176077-16129301-16131148-145698290-16131365-16131364-16130388-16130389-16130967-145698310-16128815-16128816-16129459-16130751-16130632-16130633-16131545-16128537-16130495-16130272-16130168-16129172-16129173-145698285-16130156-90111628-16130208-226524734-16131519-16128801-90111491-16132186-16130717-162135908-16128109-16129341-16129342-16131063-16131279-16131280-90111149-16128542-16130428-226524741-16130429-16130643-145698292-90111421-16131504-16128154-90111180-16130208-226524734-16130368-90111234-16129925-16129011-162135900-16130990-16132220-90111291-16129493-16131912-16129765-16129766-16131703-16129482-16130311-16130312-49176358-16128570-16132135-16130369-16129204-145698299-90111678-16131868-16130396-16130686-90111253-16129303-16130720-16129010-16129011-145698306-90111187-16128724-16130632-16130633-16128711-90111603-90111255-90111256-16129533-16130309-16130708-16130393-16130394-16131078-90111383-16130026-145698318-16129007-16129168-16129167-16129558-16130600-16130095-16130096-16128548-16131895-90111205-16131467-16131069-16131070-16130311-16130312-16130700-16130701-16131846-16132220-90111262-90111476-49176193-16130100-226524739-90111489-16130682-16130008-90111380-16128375-16129123-16131892-49176479-49176480-90111602-90111286-16131706-16129482-90111262-16129341-16129342-145698224-145698254-16129365-145698282-226524741-16130643-145698306-145698309-145698344-16128681-16129419-16131356-16131639-16130632-16130633-16128106-16128481-90111378-16130639-16130640-16129925-94541110-226524706-16128993-16129973-90111235-16130977-16130339-16130843-16130338-49176119-145698300-16131817-90111646-49176400-16128811-90111166-16131852-16129650-16130543-16130904-16130906-308209621-90111415-16131549-226524758-90111675-16131845-90111303-16128548-16131274-16131275-16128869-49176414-90111199-94541103-16130652-16132138-90111255-90111256-16128415-145698341-16131881-16129659-94541130-16128107-16128815-16128816-16129658-16131659-16129244-16130522-90111465-16129624-226524713-16128367-16128366-16131849-16128628-90111158-16130268-16128134-16129619-16128279-16128311-16129640-16130049-16128968-16130580-16130581-16131458-16131238-16132089-90111503-16130768-16132090-16128718-16128779-16128099-16129826-16128203-145698224-145698254-16129365-16129447-145698282-145698306-145698309-145698344-16129975-16131823-16131101-16130360-90111435-16129460-16130999-16132220-145698292-145698260-16129433-90111407-16130186-16129211-145698223-16128417-16128822-16129570-16132194-16128332-49176125-16131020-90111103-16129870-16129871-16131881-16129805-16132161-16132162-16128984-16130158-16128809-16128810-16131327-90111307-16128012-49175991-16128605-49176127-16131387-16131718-16130667-16131470-16130584-16131427-16131927-16130168-16128540-90111688-16129367-90111615-16131282-90111587-16132113-16127999-16129697-16128025-16131817-16131268-16131390-16131391-16131815-16129652-16129653-90111129-16128398-90111554-16131072-16131481-16131436-16131437-16128034-90111556-16130672-16130671-16128478-16131836-16131384-16131884-16131885-16128780-16129736-16129737-16128266-16129119-171701682-16129786-16129545-16130279-16130280-16129870-16129871-16130583-16130613-94541127-16131473-16129801-16129802-16131200-16131640-16132130-16128644-16128645-16128935-145698239-16128893-16129633-49176414-90111728-16128519-16128748-16130394-16131331-16128749-16129341-16129342-16130393-90111492-16130722-90111628-16131519-226524741-16130643-16131320-16131319-16131639-16130296-16130297-16128984-162135896-16128765-16129123-90111295-16129676-49176138-226524724-16128740-16128165-90111630-16130296-16130297-16131314-90111187-49176415-16129160-90111083-16128822-16130745-16129424-16128527-145698229-16128543-94541112-16130620-16130682-94541130-90111489-145698278-16132201-16130424-90111448-90111309-16130114-16130603-90111680-16131224-16129089-16130052-90111718-16129090-90111388-16128107-16129198-16131104-90111168-16128832-90111281-49176408-16130950-16130951-16129847-16128828-90111685-16131916-16130604-49176333-16130723-90111339-16129781-16128468-16128469-16130331-16128676-16129701-16130430-145698248-16129182-16131636-16129574-67005950-16129125-16130146-16130513-16130998-145698309-16131740-16131226-16131227-90111611-16129680-16129681-90111129-16131852-16128398-16128639-16129341-16129342-16129373-145698255-90111552-49176118-16130296-16130297-16132080-16128782-16128781-90111250-16130755-145698309-16130887-90111702

CRS54 90111476-49176025-16128437-49176293-16129732-16129733-16131949-16128417-16129561-16128659-157783150-16131126-16131127-16128854-90111363-16128417-16130153-16128763-16128896-16130976-162135898-16129099-16130049-16129086

CRS55 16129511-16129855-16129929-16130168-16128871-16129808-16129496-16129495-16128203-16128604-16130757-94541126-16131432-90111655-90111603-16130499-90111440-16130844-16128311-16129633-90111389-16130186-16131010-90111731-94541112-90111407-49176474-16132117-90111732-16129452-90111440-16131389-16131418-16131852-345452722-49176263-16129755-90111198-16129557-16129139-16130187-145698288-226524735-16128961-16128962-16132063-308209623-90111622-16132003-16128540-16131490-16130011-16130010-94541138-16129378-16129501-16130755-90111655-16128318-16129630-16132188-94541134-16130204-16131011-90111286-16130999-162135908-16131949-16130186-90111103-90111295-90111407-16131736-145698243-226524713-16129703-90111603-16131796-16132138-145698244-16129702-16131216-16131795-145698342-90111123-90111332-16131395-16129743-16130814-90111575-16131653-226524713-16129977-16129970-16131806-16128914-16128915-16131217-90111295-16130104-16130756-16131427-16128375-90111631-16131535-16131760-49176017-90111144-16128547-16128630-16128631-16128654-16128655-16129520-16130022-16130641-90111575-90111665-16131852-90111407-16130272-16130428-16128012-49175991-

16130186-16130429-16128657-16129615-16130391-16130967-145698310-16131935-16128481-90111489-16130682-16130835-16131416-16131143-49176358-16131117-16130747-49176329-16128755-16128543-16128602-145698229-90111154-16131104-16131498-16131609-90111083-16128813-16128814-16130301-16130461-90111497-16131217-16131395-16129187-16130158-16128705-16128706-16128781-16128605-16129100-16129755-16131110-16128747-145698310-16130967-16128681-16128755-16131356-16131771-16128034-90111081-49176017-16128528-16128605-16129460-16129658-16129659-90111575-16131314-16131799-16128906-90111492-16131104-90111663-90111440-16130439-16130722-16131942-145698342-90111361-16130974

CRS56 16128506-16131257-16129266-90111106-16130717-90111491-16130557-16131919-16130348-16131371-16131370-16128025-90111404-16130473-16129505-16130804-16131217-16131306-16131281-16131328-49176391-16128636-16131928-16128757-226524756-16130134-49176343-16130479-16128801-16130577-16131574-16129574-16128275-16131665-49176459-16132060-16128671-16128877-16130041-16131919-90111433-16128553-90111379-16130007-16128553-345452725-16129699-16129830-16129996-16130067-145698345-94541116-16129721-16130894-16129831-16130383-49176390-162135918-16131820-16131766-16128790-16128550-16129253-16128757-16130686-16132193-16131415-16128055-16128055-16128214-16128828-16130170-16130170-16130248-16130257-16131059-16131327-90111455-16131665-16129698

CRS57 16132213-16132214-90111293-226524739-90111155-16129340-16131958-16128773-16132188-94541134-16129630-16128452-16128451-16128576-16128373-16130843-49176011-16128299-16128255-90111303-16129064-16128703-16130114-16131019-16131020-16129065-49176077-16131970-90111255-90111084-16128656-16129478-16128575-16128576-16128900-16129630-16128318-16128348-16129640-16129778-16130224-16130441-16129855-16129854-16130011-226524726-16130010-16131202-16131203-16132188-94541134-145698229-16128543-90111287-94541112-90111562-90111433-16131133-16129244-16130258-90111418-94541126-16131432-16128548-16128208-16130682-90111100-16130123-16131935-16131495-16128258-16128315-16128316-49176474-16130898-16131046-16131047-16131816-16130755-16131710-16128966-16128967-90111121-226524739-16129455-16131101-16131314-90111626-16130692-16130693-16129530-16128106-16128773-16130093-90111128-16128378-145698274-90111310-16129601-16128279-90111154-16128602-16129011-16129633-16129166-90111631-16131535-16129410-16129411-16129736-16129737-16128705-16128706-16128790-16129101-16129102-16130324-16130325-90111550-90111665-16131887-16131097-16132068-16128734-16128735-16130824-90111627-16131760-16129978-16131653-16131387-16130432-90111183-49176311-16129204-90111269-16129382-49176418-16131104-90111575-16130747-16130999-16129460-16132220-16131104-49176293-16129846-16128839-16130396-226524748-16130965-16130966-16129065-49176077-16131979-90111694-16130096-16130095-16128501-16129122-94541134-16128502-16130520-16132188-16128567-16128568-16130231-16130923-16131391-49176497-16128435-16130924-16131390-226524765-16129419-16130745-16131387-90111454-90111701-16128255-16132012-90111494-16130749-90111344-16129848-90111575-16130999-16128893-16130424-90111448-16130614-16128748-16128749-16131942-16129387-16129388-16131970-16132186-145698224-16128740-145698254-16129365-16129452-145698282-226524741-16130643-145698306-145698309-16131342-16131343-145698344-16130147-16128607-90111444-16131747-49176442-16132035-16132036-16132188-16129100-345452718-345452719-94541134-90111375-16130771-16131892-90111305-16128871-16128937-94541107-16130897-16128575-90111375-16129500-16129511-90111121-90111181-16130687-16128789-16129308-16131895-16131155-16131896-16129739-145698325-49176370-221800781-16129289-145698255-16132200-16128488-16128489-16128654-16128655-16128723-221800781-16129289-16129373-16128291-16131653-16129236-16129863-16129455-90111684-16130835-16129017-16129018-16130522-90111465-16131857-16130153-16130462-16130463-16128740-49176402-16131624-16128425-16130757-16131544-16129768-16131504-90111157-16131503-16128705-16128706-16128778-90111250-16130428-16130429-16130945-16131384-16129702-16130946-16131104-16129165-90111503-16128604-16129345-90111264-16129569-16130084-49176361-162135910-94541107-226524725-90111347-90111690-90111691-16131836-16128154-16129855

CRS58 90111083-16130530-90111631-16131535-16131840-16129179-16129180-90111378-90111497-16131217-16128421-16130524-16129768-162135908-16130780-90111433-16130278-16128443-90111440-16132003-16128481-90111454-16131793-145698346-145698270-16129616-16131148-16129179-16129180-16128822-16128203-16130960-16130961-16131881-145698341-90111158-16128628-16130771-16128980-16129064-16129367-16129324-16127995-90111214-16129075-49176489-16129819-90111348-16128195-16128196-90111718-90111288-16129485-16128886-16128843-16128844-16129858-16130923-16130924-90111084-16131314-49176087-49176088-49176089-16129786-171701682-16132054-16132055-90111611-16128194-16131104-16131966-16131967-16128606-90111636-16129658-16129659-16128989-145698270-16129616-90111180-16128869-90111468-16131524-16131525-16129269-16129631-145698340-90111510-90111509-90111238-90111536-90111535-90111489-16130682-16129342-16129341-90111083-16129100-16129388-16129387-16129665-226524715-16130684-226524742-16129294-16129841-16129376-16131531-94541128-16130064-16130065-16128473-145698276-16128474-49176140-16129057-90111325-16129713-16130882-90111680-16130807-49176373-16131442-16128007-16128008-16128257-49176295-90111724-16131836-16131216-16128528-16128291-49176207-16129702-16130742-16129703-16130743-16131765-16129730-16128674-94541101-16128675-16131104-16128767-16128766-16132068-226524762-16129375-16128264-16128718-16130333-16130334-16131325-16131279-16131916-90111685-16128473-16128474-16130921-145698274-16130920-90111728-16128481-16129013-345452718-345452719-162135906-16130871-16129949-16128657-49176497-226524765-90111158-16128628-90111184-16131408-16131407-16130755-16129035-16129036-16129849-16131458-16129255-90111171-162135897-16131030-90111151-16128588-16131885-16131884-16129399-16132127-90111702-90111703-90111252-16131544-16129300-16131053-16128654-16128655-16130231-16130230-16129216-16129217-145698260-16129433-16130620-16128639-145698224-145698254-145698282-16130643-145698306-

145698309-145698344-16129365-226524741-16130880-16130879-16128723-16131813-16128896-16130107-16130108-
16130242-16128465-16128633-16130124-16130125-16128468-16128469-94541118-90111399-16130745-90111083-16131217-
16128794-16131177-16129160-16128012-49175991-16128952-16128156-90111728-16129003-16131050-16131049-94541109-
16130147-16130175-16130176-16131356-90111361-16130702-16128279-16130163-16128570-16131873-90111679-16129556-
16130069-90111652-16131671-16130780-16128537-90111253-16129303-16131217-49176358-16132038-90111707-16128092-
16128396-16130737-16130584-16131334-49176170-16130923-16130924-16131504-16130168-16130168-16131598-16131880-
90111561-16130175-16130176-16131890-16132170-90111622-16131490-16128668-16131305-16128156-16131238-16128822-
16130939-90111121-16130275-16128105-16128106-16131155-90111156-16128962-16129387-16129388-16128961-90111310-
16129601-90111154-16128602-16129387-16129388-90111494-16130749-16128527-16130430-90111581-16132089-16132090-
90111370-226524728-16129244-16130504-16130505-90111522-16130867-16128295-49176343-16130319-16131275-
16131274-16130306-16130430-16131486-90111114-90111286-16131949-16130339-16131065-16131916-16130338-90111685-
90111568-16128008-16128007-16131896-16132177-16129494-145698252-90111137-16129452-90111316-16128040-
16131895-90111497-16129057-90111246-16129265-16130306-226524711-16129301-16129447-145698260-16129433-
16128602-90111154-16129420-90111266-16131967-16131966-16129644-16131935-90111552-16131934-226524761-
16132161-16132162-16130071-16130070-16131488-90111084-16128857-16128807-90111175-16130619-16131486-16129150-
16129149-16129919-16130546-16128944-16129563-16128227-16128228-90111155-16128966-16128967-90111268-16128134-
16130580-16130581-16131536-90111209-16129029-16128674-94541101-16128675-16128564-90111154-16128602-16130275-
16128156-16131296-16131295-226524765-49176497-16132087-16129806-16129805-16130738-16129123-16130022-
226524741-16130643-16129513-16128547-16130430-16129004-16131498-16131455-16131456-16129335-16129504-
16129102-16129130-16131760-16130296-16130297-90111309-16129660-145698256-90111429-16130376-16128458-
49176489-16129697-16128904-16128564-16129187-16131970-16129916-90111362-16130324-16130325-16129854-
226524726-16128147-16128148-16130946-16130945-16129884-16129885-16128012-49175991-16130371
CRS59 16130965-16132188-16130966-94541134-16128576-145698229-16128543-16128258-16132188-94541134-16132188-
94541134-16132115-16132114-90111675-16131845-94541112-90111448-16130424
CRS60 226524762-16128217-16129841-16129739-16129759-16132099-16131974-16129511-16130150-16131119-16130825-
145698281-16129042-16128534-90111321-226524711-16130584-16128694-16130338-16130339-16131065-16131890-
16128203-16129367-49175994-16130075-16128674-16129662-16129934-226524761-16131498-16129929-16130306-
16130977-145698345-16131712-16131712-16128058-16128057-16129378-16131482-16131916-16131483-90111685-
90111433-145698340-16128639-16128271-16128272-16128570-16131307-16131308-16128676-16130349-90111428-
16130269-16131108-16131109-16132000-16128026-16128594-90111235-90111262-16129501-16129520-90111343-16129927-
16131172-16131819-16131172-145698260-16129433-16131890-16131458-16132003-16130885-16130886-16130064-
16130065-226524746-16128596-162135897-162135899-16131011-16131536-16131244-16128295-145698224-16128676-
90111163-90111171-16129146-16130724-162135908-16131019-16131377-90111274-49176282-16131851-90111133-
145698258-16130530-145698331-16131626-16128968-16129616-145698270-16131148-345452722-49176263-16128473-
145698276-16128474-49176140-90111274-16129441-90111730-16128657-145698258-90111282-16128989-16132099-
16128988-16130309-16128527-16130512-16128396-145698230-16128026-16128407-16128408-16129805-16129806-
16130348-90111726-16129076-16128435-16131765-16128912-16129496-16129562-16129757-16130657-16131823-16128004-
90111159-16129326-16129495-16129863-16129121-16130224-16130602-49176401-16128151-16128636-16131932-16128321-
90111285-16131048-145698334-16128488-16128489-16129335-16129504-145698276-49176140-16131782-90111665-
90111476-90111611-16129205-16129783-16130967-16131395-90111429-145698310-16129079-16131856-16129078-
145698339-16131445-16131446-16131963-90111568-16131470-145698331-16131626-16130272-16131916-90111685-
16128224-16132042-16128223-16132041-16131988-16128357-16128990
CRS61 16129511-16131294-16132208-90111747-16131104-90111743-90111083-16129123-226524721-16130585-16130168-
49176257-90111384-16130771-145698274-16130617-90111481-16131591-16128773-90111384-16130312-145698331-
16130311-16131626-94541090-16128970-226524719-145698230-16130844-16128854-16129303-16131944-90111253-
16131945-16132177-16129127-16129126-226524725-90111347-49176077-16129065-16130311-16130312-16129539-
16130965-16130966-16128203-16130335-16130336-16128547-16131030-16128034-16128271-16128272-16128364-90111125-
16129072-16130430-16131294-16132202-16131526-16129211-16129244-90111428-16131765-16131766-16131857-16131463-
16131464-16129130-16132202-90111427-90111100-16128208-90111123-16129378-16129700-226524724-16129794-
16129795-16131140-16131141-90111280-90111389-16128315-16128316-90111122-16128481-345452718-345452719-
16129650-145698341-90111253-16129303-90111360-16131395-145698340-16132220-226524762-90111122-16128817-
16128818-16130350-16130430-49176350-16129558-16129559-16128481-145698229-16128543-16129441-16130698-
16128755-16130430-16130751-16131677-16131678-16131843-16128723-90111103-16128585-16131591-49176479-49176480-
16129659-49176293-16131765-16131766-16130010-16130011-16131653-16131703-16131704-16128694-49176351-16131101-
90111389-16131390-16131391-16131364-16131365-16132082-16132083-16132183-16131689-16128805-16131688-16129100-
16131770-16130835-94541127-16131473-16129004-16132220-16129244-16129511-16130169-16128822-16128073-16129934-
16130400-16128443-16131951-16131952-16129093-90111489-16130682-90111079-90111122-16129338-16130172-16131892-
16131284-16128843-16128844-16128773-16130119-16132000-145698346-49176377-16129981-90111495-16130429-
16130428-16131780-90111214-16129075-16130272-16131247-90111617-16131529-16128012-16128013-16129583-90111629-
16131879-49176479-49176480-16132197-16132198-90111382-16129805-16129806-16129978-16130580-16130581-90111299-
90111300-16129495-16129496-16129619-16130518-16129101-16131284-16131387-16128573-16128574-16129101-16129102-
16129102-16131034-49176401-16132026-16128227-16128228-16128914-16128915-16132195-16130708-90111268-16129410-

16129411-16130655-16132166-49176071-16128979-90111615-16131426-16131760-16128205-16128206-16128605-90111180-16131360-16129428-16130130-16128101-16128383-16128384-16128770-16128773-16129123-16129630-16131152-16131388-16131389-49176377-16131498-90111651-16128203-16128481-16129390-226524725-16130424-94541127-16131473-16131935-90111347-90111448-16131104-16129985-16129455-16130300-16131571-16129863-16129557-90111440-16131736-145698342-16130395-16129558-145698244-145698243-16129579-16129578-16131020-49176442-16132186-16128012-49175991-16129826-16130272-16130428-16130429-16130543-16131019-16131747-16131979-90111694-90111510-90111509-16131377-16129202-16131941-16131529-16130755-16130882-16131314-16130477-16131391-90111154-16128602-16130584-16131390-16130325-16131126-16131127-16130324-16128366-16128367-16130129-16128264-16128417-16128906-16129478-16128665-90111444-16130667-145698344-16128255-16128864-16128865-16129405-90111383-16130026-16130745-16131360-162135894-16130049-16131840-90111685-16131916-16128195-16128196-16131772-16131880-16132077-16132076-16128515-145698340-16132194-16130175-16130176-16132186-16131819-16130987-90111122-226524735-16131316-16128570-16129426-16130602-16132026-16132038-90111707-16131944-16131945-16130755-16128763-16130368-16130013-16128291-90111137-90111144-16128605-16129723-16129927-90111440-90111474-16131382-16131383-16131736-16132213-16132214-16131458-90111622-16131808-16131490-90111114-16128134-90111316-90111552-16131455-90111685-16131916-16132114-16132115-90111123-16129244-16131456-16131935-145698291-226524711-16128986-49176293-16131852-145698342-16128706-16128705-16128789-16130999-16131575-16130272-16130146-16129460-16129971-90111562-16131133-16131202-16131203-90111495-16128904-90111190-16129387-16129388-16129658-16129659-16129755-16130110-16130524-90111666-16128674-94541101-16128675-16130225-49176377-90111505-16130779-16131857-16131498-16128105-16128106-16129752-16131780-90111702-90111703-16130990-49176377-16129562-16128124-16128125-16131658-49176416-16131770-16129035-145698244-16129036-145698243-16129661-16128966-16128967-16129495-145698229-16128543-16129496-94541112-90111559-49176329-16131117-16129901-16128171-16128851-16128955-16129517-90111363-16131591-16131819-16128956-16129516-16129945-16131813-16129338-16131958-16130745-16130187-145698288-16130583-16131445-16131446-16129198-16131407-16131408-16129261-90111286-16128828-16129312-16128828-16128513-16130723-16131718-90111718-16128904-16130158-90111190-16131582-16131583-16129185-16129186-16130100-49176193-16128303-16131170-16131591-16131979-16128134-16128302-90111694-16129863-90111670-145698338-16128364-90111125-16129630-16131377-16131155-16131882-49176479-90111147-16129511-49176480-16130707-16129185-16129211-90111448-16131127-16129186-16130424-16131780-16131715-16130147-16131681-16131949-16128585-16130844-16131591-16131688-16131689-16130717-90111491-145698342-49176479-49176480-16132188-94541134-16128367-16128573-16128574-16129187-16130885-16130886-16130999-16131217-16131389-90111728-145698346-16130756-16129561-90111286-90111449-16130755-16131949-16128669-145698231-16128843-16128844-16129631-16129919-16129011-16131203-16131955-16129010-16131202-16131388-16131544-90111428-16128570-16130505-16131503-16128607-90111246-16129265-16129697-16130504-16131504-90111278-16129422-16131305-16128628-90111158-16128833-16128834-16128896-16130620-16128605-16130608-90111165-49176260-16129348-16129349-16129954-16128679-16128684-16128685-16129927-16128896-16130875-16131851-16131773-16131774-16130647-90111125-16128364-16128747-90111718-16131718-16131954-16130771-16129739-162135904-16129952-145698309-16128367-16128366-16128109-90111310-49176480-16128105-16128675-90111238-16129601-49176479-94541101-16129847-16130172-16128795-16128796-226524716-16132003-16128205-16128206-16129123-16129338-16129558-145698309-16131970-16129630-16129841-16130350-90111497-16131924-16130749-90111494-16129714-145698262-16129715-16132115-90111166-16129675-16130049-16132114-16128164-16129736-16130671-16130715-90111702-16128203-16129737-90111361-16130672-90111703-16132112-16130148-49176489-16127995-16130824-16128272-16128675-16129179-16129925-16128271-94541101-16128805-16129180-90111638-16128513-90111147-16129252-16131395-16130965-16130966-16130965-16130966-90111312-145698269-16130967-16129739-16129414-16129160-90111214-16129075-90111573-49176351-16128488-16128489-16131529-90111651-16128770-16131152-16129428-16130999-16128779-145698224-145698254-16129365-145698282-145698306-145698309-145698344-16129919-16131653-16128844-49176170-90111557-145698344-16128602-90111154-16130146-16131970-16131377-16131467-16132068-90111252-16129300-90111438-145698334-16128989-16128625-16128626-90111214-16129075-16131591-145698341-16131881-16130430-16130751-16128501-16128502-16128705-16128706-16128605-16128926-16129126-16129127-16129771-16130281-16130620-16130939-16128925-16129807-16129626-16129020-16129338-16131262-16128417-16128473-16128474-16129973

CRS62 16131951-16131952-90111547-90111548-16130668-16132070-16129367-16129557-16128590-16128591-90111489-16130682

CRS63 16129460-16130999-16132220-16129123-90111295-16130597-16128321-90111214-16129075-16130393-16130394-90111537-16131364-16131365-145698309-16128968-16128782-16129969-16131456-16131455-16130835-16131815-90111603-16129211-16130583-16128779-16130307-16130308-16129676-49176138-90111407-16130186-49176474-94541126-16131432-49176207-90111684-90111391-16130939-16129455-16128780-16129847-16128576-16130835-90111481-90111440-16130617-16131736-49176356-16131591-16128570-16132003-16132133-16131387-16131330-90111622-16131490-345452722-90111114-49176263-16128754-90111407-16130186-16130815-16128134-16130272-16129801-16129802-16131200-16130168-16130581-16130580-16129236-16128893-16129973-16131034-16131736-16130964-16131247-16129123-16128676-16129676-49176138-16129054-16130168-16129577-345452718-345452719-16130187-145698288-145698341-16131881-16130965-16130966-16129102-16131203-16131202-16130965-16130966-16129826-90111552-16128008-16128007-16128527-16129658-16129659-16130093-16130965-16130966-16130306-16131970-90111186-145698230-16129338-49176463-90111363-16129929-16131455-16129863-16131456-16131057-90111083-16129102-226524732-16128886-16128681-16131356-16129345-90111264-16130965-16130966-16129663-16130049-16130049-16131388-16130965-16130966-

16132202-16130814-16128684-16128685-145698256-16129261-16128605-90111295-16129308-16129309-16128303-16128302-16131148-16128984-90111147-16128828-16130835-16128605-90111277-16129413-16130546-16131490-90111622-16128773-345452718-345452719-16131996-16128548-16130393-16128519-16130394-16128321-16129387-16129388-16129459-90111123-16132135-16130401-16131049-16131050-16128915-16128914-16128407-16128408-145698344-16131442-49176373-16128311-16131200
CRS64 16128900-16129884-16129885-16131469-49176398-16129751-16128766-16128767-16130835-16131710-49176434-16129762-90111336-16129755-49176247-16129159-16131382-16131383-16129428-16130737-16129985-90111523-16130333-16130334-16130088-16131388-16131385-90111494-16130749-16131098-16128233-90111407-16130186-16128183-16128981-16129072-90111097-49176004-90111581-16128849-49176343-16128848-90111178-16129637-16130103-16131627-16128474-16128473-16130558-16130969-16130970-16128917-16131411-16131389-16128815-16128816-16128105-16131836-16131903-16129160-16128560-16128135-16129197-16129871-16129870
CRS65 90111743-16128192-16128193-16131202-16131203-49176377-16128738-16129414-90111489-16130682-16128548-16129017-16129018-16128300-16128928-16128929-16131227-16129460-16131226-16130522-90111465-90111657-90111321-90111389-16130912-16129826-16131806-90111286-16129557-16129630-16130587-16131704-16131873-16131387-16131703-90111679-16130672-16130755-16130671-90111121-16128950-16128407-16128408-345452718-345452719-90111102-16131857-16128481-16129159-90111242-90111497-16131126-16131127-90111096-16129854-226524726-16129660-226524751-16131506-16131542-16128586-16129929-16131074-49176414
CRS66 16130530-16130871-16132035-16132036-90111552-16131611-16131125-16131612-90111151-16128588-226524726-16129854-90111444-16131076-16131077-90111576-16130807-49176408-16128321-16128896-16128654-16130755-16128655-16130687-145698336-16129099-90111454-16130780-162135898-16131759-16131758-145698340-16131959-16129027-16129598-49176129-16129739-90111401-16128576-16128886-16130307-16130308-16129598-49176129-16130129-171701683-16130130-49176239-16129904-16131881-16129905-145698341-145698270-16129616-16129863-16130587-16131126-16131148-16128605-16129927-16130946-90111568-16131275-16131885-90111735-16129139-16130945-16131274-145698334-16131884-16130395-16131754-162135899-16129146-16129659-16129658-16129978-16130212-90111453-16131295-16131296-16128074-90111629-16131482-16131483-16131819-16128542-16128738-145698306-90111149-16129221-16132188-94541134-90111364-16131148-16128396-16128926-16128925-16130456-16131935-16129747-90111334-16130826-16128556-145698255-16129373-16129914-16128445-16128111-16128694-90111244-90111245-16130278-226524763-16128272-16130244-16131840-90111269-90111085-16129382-16128435-16130708-49176045-226524704-90111174-16129051-16129539-226524751-16131307-16131312-90111211-16131308-16131313-16128160-90111266-16129452-16130049-16130520-145698344-16128147-16128148-16128896-90111154-16131104-90111603-16131611-16128515-16128602-16131612-90111743-16128759-16128758-16130282-16131170-16132134-16128012-49175991-90111495-49176329-16131892-16129452-16131020-16131117-90111495-16130301-16131935-16131373-16131934-16131217-90111732-90111731-16129804-90111596-16131488-90111655-16131998-16128665-90111597-16128527-16129159-16129461-16131792-16131461-16129675-16131415-16132040-16128015-16128016-16128249-16128250-16128259-16128260-90111199-94541103-16129845-16129846-16131316-16132114-16132115-16132120-16128265-90111643-90111171-16129862-162135897-49176045-16128188-90111665-90111438-16129266-16129798-16129575-90111349-16130519-145698322-16131610-16128477-16129236-90111114-90111114-49176361-49176373-16131442-16132038-90111707-16128039-16131824-16131217-16128239-49176171-16131217-16129796-16131426-90111615-49176474-90111288-16129485-16130963-288551665-16129042-16128173-16129459-16131021-16130734-16128258-90111330-16131247-16128366-16128367-16129614-16130584-49176489-90111137-16131148-90111617-16130400-16130429-16130428-16129826-90111429-16130271-16129972-16129680-16129681-16128573-16128574-16128520-90111294-16130231-90111685-16132013-16130230-16131916-16128250-16128630-16128986-49176140-16130175-16130715-16130844-90111665-16131881-16128631-145698276-16129776-16130176-16132060-16129123-16130725-145698233-90111256-16131857-16128737-90111255-16129460-16129533-16129581-16131217-16131536-16128755-16129971-16130867-90111391-16131389-16131846-16131892-90111690-90111691-16128633-16131020-16131591-16128828-16129525-16130113-16130168-16130168-16128584-16128477-16130401-16131470-16131486-90111728-90111406-16129761-90111235-16130325-16128501-16129420-16130478-16128611-16130324-16128340-16128513-90111147-16129149-16129150-16129455-16131170-90111378-90111237-16129213-16131148-90111295-16127999-16128594-90111360-16130742-16130743-16129640-90111474-16131747-49176442-16130311-16131531-16130312-16130441-94541128-90111550-16128465-16129236-16129781-90111339-16129975-16130187-145698288-16130668-49176267-16131019-16131389-16131529-90111561-16131331-16130771-16131330-16130738-16131714-16131961-90111214-16129075-16129925-16130443-90111489-16130682-49176286-90111514-16130974-16131059-16131104-226524753-145698342-90111149-90111083-16128542-90111158-16128628-16129575-16130087-90111171-162135897-16129847-16130887-90111645-345452722-49176263-16128196-16131780-90111702-16128195-16129975-16131916-16131942-90111703-90111730-16131766-16129198-16131765-16129407-226524714-90111280-90111405-16130186-90111407-16129119-145698249-90111665-90111679-16131873-16131916-16128970-90111509-90111685-16129236-145698270-16129616-16130835-16131145-49176361-16131591-16131780-16129113-16129977-90111186-90111253-16130780-16130924-16131390-16129121-16129303-16129796-16130923-16130990-16131391-16128147-16128148-16129626-16129671-16130205-90111413-90111566-16131582-16131583-16130013-16131086-16129640-16131085-16128694-16129501-16131395-16129308-16129309-16129619-16129658-16129659-16130708-16131390-16131891-16131949-16131879-16129959-145698220-90111372-16128506-16128855-16129806-49176350-16129805-16129670-90111443-16128068-16128367-16129102-145698256-90111361-145698293-16130403-90111481-16130617-16130708-145698312-16131119-16132026-16128540-90111121-16128807-16129084-16129429-16130503-16131300-16131342-16131365-90111175-16129003-16129452-90111462-49176249-16131299-

16131343-16131364-16128007-16128008-16128147-16128148-16128367-16129211-16129376-16129378-16130113-16130393-16130394-16130476-16130477-16130742-16130743-16131202-16131203-16131364-16131365-16128324-16130966-16131389-157783149-16130965-16128463-16128464-49176361-162135897-90111575-16131435-16132056-90111122-90111171-16131552-16131575-16132057-16128654-16128655-90111083-16128258-16130175-16130176-16130543-226524726-16129854-145698318-90111681-16131887-16128096-16129513-16129864-16129901-49176377-90111703-16128097-16131469-90111702-16128614-16128722-16131305-16128721-16130441-16128501-16128502-16128636-16129034-16130152-16131920-16130599-16129179-16129180-226524732-16128234-16128280-16129990-16129990-16130605-16128528-16129139-16130962-145698258-90111274-16130094-16131893-16129703-145698338-226524721-16129702-90111455-90111670-16128676-16131011-90111622-16131524-16131813-145698341-16128026-90111440-162135908-16131490-16131525-16131881-90111728-16130638-16131633-49176282-90111084-16128952-16128970-94541090-16129676-49176138-90111363-16130755-90111557-90111561-16131482-16131483-16131882-16130316-16129064-16128556-90111167-90111130-16130168-16130826-16131034-16131448

CRS67 16128980-16128981-16128375-16131856-145698339-16130113-16128291-16131950-16128060-16130650-16129341-16129342-145698248-16129182-16130780-345452715-16128252-16128224-16128223-16132114-16132115-16128815-16128816-90111634-16131333-16131445-16131446-90111562-16131133-16131754-16129794-16129795-16129697-90111557-16128790-16128270-16128421-16128675-90111606-90111690-16128674-94541101-90111605-90111691-16130224-16129380-16129379-49176330-90111647-16128187-94541107-90111634-16129643-16129644-90111497-90111084-16129054-16128427-16131284-16130815-16128217-16129090-16129089-226524711-16131892-16128271-16128272-16128659-157783150-16129152-16129151-16130998-16132150-16132176-16131471-90111739-16130093-16128329-16130655-16128280-16128383-16128384-90111250-16129721-16129157-16129158-16129577-90111156-16130325-16128892-16130089-90111361-16129898-16129848-90111184-90111539-16131542-16131851-90111286-145698329-16130945-16130946-16129604-90111121-16132120-16128477-16131504-16131503-90111441-145698336-16128782-16131316-16131486-16131808-16131843-16129592-16129852-16131391-16128295-16128843-16128844-16129622-16130081-16131799-90111634-16131633-16129139-90111509-90111510-16129075-90111214-16131474-16130882-16128843-90111380-16130008-90111295-16129714-16129715-16128430-16128455-90111136-90111440-308209622-90111640-226524718-16130147-16128655-16130268-16128654-16132054-16132055-16131474-16129020-49176407-90111206-16128849-16128848-16128000-16131519-90111628-16129747-90111334-16128968-49176276-16128928-16128929-16128217-90111147-16130319-16131294-162135897-90111171-16132012-90111701-145698293-16131712-16129904-16129905-16128163-16129901-16130720-16129614-16129324-145698220-90111718-16130476-16128787-16128788-16129778-16130477-16130305-90111100-16128208-16129487-49176373-16131442-16131416-16129179-16129180-16129233-16129232-16130545-90111307-49176127-16128968-16128459-16131611-16131612-16129348-16129349-16131390-16130251-16129499-16128074-16128445-16128779-16130738-90111708-16132120-16128127-16128460-16128461-16131740-16128593-90111702-90111703-16131846-16128111-90111271-49176426-16128502-16128501-16128981-16128980-16129390-90111177-90111139-16129693-16129694-16130231-16128034-16131313-16131312-16129613-90111305-16129612-16128779-16131117-16129561-49176329-49176404-16129230-90111476-90111083-16128957-49176407-16129450-16130897-49176045-16129778-16130688-16128926-16128925-16129969-16129076-16131155-16128723

CRS68 90111246-16129265-145698260-16129433-145698345-90111631-16131535-16131111-16128417-90111183-90111675-16131845-16131294-16130583-16128415-90111627-16131155-16131011-162135908-16131364-16131365-16128534-16129622-90111503-16130768-16128570-16129631-16131119-145698340-90111246-16129265-16128264-16130049-16130504-16130505-16130278-49176295-16129065-49176077-16131722-90111659-16132219-16132218-16129506-16131678-16131677-16129339-94541111-16131063-16131382-16131383-16130224-16131806-16131503-16131504-90111316-16131736-16132186-16129973-16128604-16130584-49176377-16128266-16128734-16128735-16131331-16128828-16130823-16128982-16128122-16128258-16129972-16129020-90111509-16129852-16130601-16129906-16129240-16131774-16131773-16129065-49176077-16130022-16131048-90111672-16130278-16128864-16128865-16131840-90111703-90111702-16129433-145698260-16129851-16128519-16128054-16129885-16131949-16129884-16129927-16130296-16130297-16128914-16129615-90111153-16128915-16130332-145698306-16128193-16128192-16128257-16132134-16128755-16130129-16130130-226524752-16131935-90111181-16131934-90111583-162135911-16128864-16128865-16129225-90111481-16130617-345452718-345452719-162135906-16128203-16129838-16129736-16128138-16130129-16130130-16130604-16130751-16132166

CRS69 162135920-16130715-49176358-16130715-90111335-16129754-16131330-16131632-16131631-16129533-16132142-16132143-16128060-226524720-49176018-16130306-162135907-90111457-16128638-16131257-16130143

CRS70 16131028-16131023-16131024-16131023-16131024-16131806

CRS71 16131815-49176479-49176480-16128417-16128906-49176479-49176480-16128583-16130608-16128582-49176260-16129583-16131879-16128528-16129455-16130939-16129100-49176207-90111295-16131736-90111385-16130036-49176368-16131411-16129852-16130829-16131849-16130403-90111443-90111178-145698306-49176302-90111122-16128034-90111081-16128264-90111214-16129075-16131712-16128605-16129771-16130281-90111083-16129123-16129017-16129382-16129018-90111269-90111440-90111629-90111122-16129211-16128548-90111122-16128925-16128926-16129663-16129765-16129766-16128570-16129335-16129504-90111603-16129458-16130967-145698310-16128281-16128988-16129244-16131115-16130602-16130430-90111209-16129029-16127999-16130999-16131653-49176267-90111361-16128210-16132194-16129511-16130593-16131531-94541128-90111591-16130368-90111561-90111622-16131490-16129121-90111352-16129828-16129845-16129846-94541128-16129152-90111724-16131531-16131755-16129100-16129954-16130335-16130336-16130010-16130011-16128740-90111242-49176177-16130583-162135903-16130597-90111144-16129084-16130987-16130599-

16130988-16131030-90111557-90111316-16129126-16129127-16129047-90111210-16128628-16128980-16130974-16128981-16128547-90111158-226524753-16129245-90111158-16128628-16129858-145698342-226524739-16130591-16131161-49176293-16129338-16130602-16129407-226524714-16128989-16131736-16131770-90111448-16130424-16129390-16129697-16130320-16130321-49176293-16131392-90111476-90111154-16128602-16130584-16131294-16132208-90111747-16128271-16130152-16128272-16128571-16129525-16129755-90111211-49176414-16129051-16129870-16129871-16131455-16131456-16131591-16129010-16129011-16131202-16131203-16131388-16131498-90111079-16130606-16130049-16130505-16130504-162135903-16128515-145698312-16129516-16130384-16129517-16128605-16131242-16131243-90111361-16128547-16129495-16129496-16129631-49176401-16131935-90111684-90111147-16130476-16130477-16131314-16129075-16130272-90111214-16131247-16131529-16128469-16130835-16128468-16131200-16132099-49176407-16130505-16132035-16128375-16128527-16129378-16130504-16132036-145698322-16128134-16128904-90111190-16128843-16128844-16131970-16128773-16130324-16130325-16130715-16132000-16129168-16129167-16130473-16131364-16129460-145698279-16131365-16128898-16128899-16128871-90111287-16131247-16129338-16130152-16131588-16129123-16128989-90111713-16130352-16130353-16131455-16131456-16130978-90111575-90111629-49176398-16129553-90111701-16128255-16129554-16132012-90111449-16131364-16131365-90111165-16129338-16129568-90111666-16128465-16129805-16129806-49176392-49176393-90111190-16130429-16131887-16128904-16130428-90111681-90111679-16131873-16131757-16130540-16130168-90111655-16128481-90111158-16128628-90111123-16129237-90111332-145698312-16129743-16130545-90111568-16131780-16129646-16130306-16128266-16131955-90111083-90111144-16130022-16129855-16130641-16131935-90111631-16131535-16129203-226524730-90111381-90111676-16128527-226524731-16131773-16131774-16131836-90111184-16129165-16131598-16131838-16131839-16131903-16129975-16131954-16128721-16128722-16130299-16131411-16131640-90111147-16131011-162135908-16128839-16129972-16129211-90111114-16128574-16128573-145698230-90111384-16129516-16129517-16128526-16128955-16128956-90111155-90111458-16131860-16130146-16131736-16129452-162135916-90111705-16128526-16130187-145698288-16129650-16129862-16128205-16128206-90111083-16129633-16128164-16131703-16131704-16131481-16128567-90111361-16130158-16128302-16128303-16129123-16131436-16131437-90111489-16130682-90111190-16129981-145698345-16130998-16129517-16129516-16131028-16128034-16130667-90111433-90111562-16131133-16128012-49175991-16128156-16129973-16131295-16131296-94541112-16132026-16131588-90111087-16128116-16128635-16129101-16129102-16130013-16132099-90111220-49176207-16130964-16129104-16129558-90111416-16129375-16132134-16131161-16129324-16130430-16130755-16128586-16129511-16128625-16128626-16130095-90111154-16129676-49176138-16130096-16131153-16132133-16128602-16131152-16128828-16131957-226524719-16129511-16129127-16129348-16130747-16129126-16129349-16130049-145698263-16129245-16128801-16130887-16129511-226524725-90111347-16130168-16130580-16130581-16132213-16132214-90111474-16130939-145698291-16131122-16129160-90111560-16131379-16132177-90111361-16129376-16131836-16129659-16129664-49176017-16128501-16128502-16130275-16130771-16130814-16131314-16132134-16132134-16131916-16128481-16129660-90111685-16130659-16130660-49176329-16131117-49176392-49176393-16130148-16130858-16131808-16132112-16128491-16128007-16129501-16128008-16129242-16129339-94541111-16129429-16129855-145698309-16131030-145698344-16131590-16128856-16130308-16130307-16129623-16130771-16131944-16131945-16129630-90111622-16131330-16131490-145698340-16132161-16129122-16132162-16130275-16130599-16130844-90111163-16127995-16130010-16130011-16129539-16129970-16130146-16128675-16129703-16130743-16128674-94541101-16129702-16129852-16130742-16129216-16129217-16129461-16131631-16131632-16131316-16130620-16129294-90111416-16131816-90111718-90111277-16129413-16128279-16130735-90111193-16128773-49176301-90111286-16130261-90111563-16132150-90111186-16128378-90111128-16128586-145698274-16129759-16131023-16131024-16129324-16130734-90111157-16129295-16130106-16130659-16130660-16132089-16132090-16130477-16131390-16131391-16129739-90111679-16131873-16129187-221800782-16129691-16130844-16131529-16128718-16128770-16130430-16130751-16131591-16128069-16128970-94541090-16131367-16129675-16131366-16130552-16130306-16130939-16128527-90111214-90111634-16129075-16131034-16129444-221800779-16128864-16129661-16128865-16131295-16131296-16131458-16129101-16129101-16129102-16129102-16128828-16130505-90111474-16130504-16129925-90111685-90111428-90111617-16131916-90111743-90111163-16129410-16129411-16131279-16128844-16128537-16128843-16128013-16128012-16128386-90111378-16130807-16131427-90111438-90111532-16128012-49175991-90111121-16128203-16129511-90111728-16130639-16130640-90111246-16131049-16131436-16129265-16131050-16131437-16128561-226524729-226524730-16131030-90111381-16129697-49176377-16132090-16132089-16131653-16131703-16131704-16131591-16128654-16129919-16130424-16128655-90111448-16129020-16130780-16131101-49176351-16128669-145698231-16129852-16130172-16130923-16130924-16131316-90111703-90111702-16128923-16129612-90111111-16128922-16129613-94541101-16128805-16130672-16128675-16130671-90111491-16130717-16131011-162135908-16131779

CRS72 16129661-16128567-16128568-16128659-157783150-16128938-16128540-16131105-16130520-90111718-226524714-16129407-16129676-49176138-16132000-16128568-16128567-226524721-16129455-16129541-16129540-16128723-16129975-16131384-16129219-16128034-90111081-16128417-90111497-16130049-16128366-16128367-90111144-145698282-16129424-226524753-16131633-16130594-16129004-16129614-16129661-49176463-90111603-16130096-16130095-16128540-16128659-157783150-16131105-221800782-90111257-16129004-16129855-90111269-16129382-16128208-90111100-16128366-16128367-16130650-16128364-90111238-90111269-90111125-16129382-90111254-90111254-16131172-16131034-16128147-16128148-16132030-16128300-16129458-16132177-16128364-90111125-16131531-49176267-94541128-16129702-16131895-226524739-145698254-16129365-16130300-16130301-90111743-16129072-16128568-16128567-16129553-16129554-16129971-16129927-16128740-16128445-16130584-16131840-16129291-16129292-16130857-16128567-16128568-16129661-16128040-16130998-145698244-145698243-90111403-16129429-16128034-16131152-

16131153-226524741-16130643-90111739-16132176-16129072-16130084-16128012-49175991-16131387-90111444-16131887-90111681-90111316-16128573-16128574-16130999-16131217-90111087-16128116-16131356-16129879-16128780-16131591-16130885-16130886-16129237-90111511-16130812-49176377-16130395-16130084-90111084-16130824-16131316-16130169-16128227-16128228-49176377-145698229-16128543-94541112-16130999-90111084-16128437-16131387-16128095-49176025-16130208-226524734-90111622-16131490-16132133-16130269-90111622-16131490-90111190-16128904-16131873-90111679-90111455-16129122-16129539-145698276-49176140-16129545-16130887-16132026-16129324-16128367-16128789-49176474-16132109-16129428-345452718-345452719-16129676-49176138-90111739-16132176-16128570-16130175-16130176-16131427-90111702-90111703-49176087-49176088-49176089-16129826-16130799-16130800-16130684-226524742-16128904-90111190-16130632-16130633-16131217-16131052-16128723-345452722-49176263-16129335-16129504-16129556-221800781-16129289-16128192-16128193-16130824-16128481-16130657-16130871-16129428-16129626-16131795-16131796-16127999-16131392-16129583-16129613-16129612-16129093-16130205-90111413-16129650-16132114-16132115-16132188-94541134-221800782-16131105-16130643-16131077-226524741-16131076-94541126-16131432-16131873-90111679-16130745-16128970-94541090-16129017-16129018-16131104-49176358-16130924-90111286-16130923-16131949-90111250-16129640-49176442-16131747-16128527-16130147-16131318-16131958-90111147-16129378-145698309-90111125-145698254-16129382-49176137-16128364-16129365-90111269-16130684-226524742-49176358-49176373-16131442-16130208-90111180-226524734-16130745-16128501-16129429-16131843-16129562-16130632-16128502-16130633-16132114-16132115-16130594-16131411-49176368-16130844-226524713-16131202-16131203-16128481-49176074-16129179-16129180-90111268-16129691-49176401-16131740-16128833-16128834-16130999-16131934-16131935

CRS73 16129845-16129846-16129856-16129857-49176045-16132134-16128504-49176045-16131633-16128656-16129925-90111421-16131817-145698292-90111083-16132076-16132077-16131955-16128291-94541128-16129561-16131531-16128291-16130081-16129494-90111186-16129326-16129658-16129659-90111305-16129801-16129802-16129838-16131458-16128576-16129540-16129541-16132000-16131570-16129972-16130965-16130966-16131416-90111605-90111606-16128748-16128749-16130049-16130049-16128607-16129540-16129541-90111378-16130150-16131327-145698340-145698220-16130175-16131942-16128654-16128655-16130230-16130231-90111454-16131440-16131773-16131774-16132134-16128105-16130176-90111427-16128761-16129301-16129737-16130441-16130815-162135920-16129736-226524738-16131148-49176358-16131653-90111308-90111288-16129485-16130522-90111465-16130756-16130977-16131148-16129553-16129554-16129680-16129681-16128528-16129572-16129360-16128724-16128777-16131244-16129467-16129737-90111670-145698338-90111568-16129848-16130671-16130672-90111679-16128311-16131873-90111252-16129851-90111171-162135897-16129300-16131526-16130557-16130875-16128630-16128631-16129935-90111379-16130007-16128828-16131659-94541130-16128383-16128384-90111384-16130418-16130419-16131415-16131775-16131863-16128311-90111180-16131069-16131070-16130330-16129003-345452718-345452719-16130002-16130613-16132040-16132199-145698262-16131824-226524726-16129854-16130350-145698341-16128310-16129424-16129622-16132186-16131671-16130734-90111652-16128590-16128591-16129367-16129557-145698297-16129472-16130106-16129471-16128016-16130205-16131959-16129985-16131395-221800782-145698262-90111413-16131765-16128917-16129233-16130144-16129232-16129804-16129898-145698346-16128013-16130394-16128817-16128818-16129862-16130393-16130591-49176343-16131299-16131300-16128904-90111190-16128906-90111557-16132048-16132049-16128060-16130396-16128382-16129378-49176171-16129977-16131388-16131633-16131837-16128547-90111307-16128321-90111164-49176127-16129721-16129901-16130292-16130652-16131108-16131109-145698318-16131961-16128564-16129539-16129615-16129978-90111387-145698312-49176425-16131680-90111665-16128464-16130584-16132090-16128463-16129583-226524732-16130388-16130389-16132089-16128755-16129978-16128012-49175991-16128481-90111149-16128542-16128590-16128591-162135898-16129099-90111294-16129747-90111334-16129972-90111631-16131535-16129049-16131591-16132117-16128462-90111214-16130175-16130312-90111495-90111629-90111703-16128650-16129075-90111286-16130176-16130311-49176267-16131849-90111702-16130279-16130280-90111147-16128258-16128348-16129102-49176170-90111415-16130322-16131653-16128258-16128271-16128481-16129455-90111378-90111387-16131776-16131777-16130131-16129973-16128195-16128196-16128695-16129204-16129862-16130350-16129052-16130306-90111121-16128366-16128367-16128586-16128694-16128855-90111253-16129303-16129378-145698256-226524753-16130333-16128152-16128153-16130334-145698256-145698331-16131626-90111121-16131469-90111642-16128068-16128705-16131436-16131966-16128706-16128839-16131437-16131967-16130217-90111509-90111510-16129003-16128833-16128834-16129501-16130107-16130108-16130707-16131221-16131555-16128425-90111310-16129601-226524721-16130645-16130739-90111638-16131274-16131275-16131738-16131247-16129151-16129749-16129152-16130594-16128654-16131119-16128272-16128655-16132197-16132198-16129452-16129675-16130095-16130096-145698299-16130396-16131342-16131343-16131659-16130230-16128106-16130887-16131722-90111659-16129301-16129831-226524742-16131776-16132035-16130172-16130684-16131777-16132036-16128488-90111494-90111552-90111685-16132013-90111083-90111121-16128489-16130749-145698336-16131916-16132135-345452718-345452719-90111295-16128681-16129863-16131356-16131954-16128548-16129970-16130703-16131316-90111718-16128017-16128018-49176074-90111629-16129652-16129653-16128165-49176368-16130153-16131411-49176414-308209620-16130999-16129141-16130723-16131712-16128364-90111125-16128659-157783150-16128938-145698254-16129365-16129373-145698255-49176137-90111344-90111389-49176358-16128227-16128228-16129419-16128570-16128593-16129348-16129349-16129863-16130147-16130430-90111450-16128637-90111326-226524746-16129565-145698268-16128527-90111383-16130026-16128007-16128310-16129380-145698274-16128143-16129244

CRS74 16130617-90111481-94541105-16128026-90111444-90111245-90111244-16129700-16130887-16130745-16130512-90111476-49176293-16132186-90111081-16128034-16131019-16131020-90111244-90111245-16132176-90111739-16130756-16128417-16130887-16129072-16128300-16129559-16129558-16131217-16129736-16129737-16130835-90111154-16128602-16130530-90111703-90111702-90111536-90111535-345452722-16129198-49176263-16131152-16131153-16131152-16131153-16128832-16130828-16128650-16130272-16129003-16130720-16131356-221800779-16131259-16129913-16128576

CRS75 16129024-16129545-16129236-16128989-16130400-16131085-16131086-16129799-16131959-16131387-90111158-16128628-16130751-16128871-16128321-16131104-90111476-16130875-16128034-16132220-16131838-16131839-16128537-16129751-16129862-16128556-49176088-16129702-16129703-16130300-16130301-171701682-16129786-16129244-145698346-16128570-16129640-16131740-16128133-16129121-16129072-90111316-16128843-16128844-16131837-16128604-16131153-16131152-226524746-16129047-90111210-16130272-16132120-16132080-16131488-16128501-16128502-90111247-90111622-16131490-16130689-16130690-90111643-16129826-16129495-16129496-90111730-16131078-16129072-90111316-16131767-16131294-145698309-16128291-16130205-90111413-16128203-90111361-162135912-16131019-16131020-16130340-16130939-16129045-16129863-16131529-49176343-49176479-49176480-16127999-16130964-16131653-16128568-90111361-16128567-16128904-90111190-16131020-16131019-90111428-16129950-16130689-16130690-16129801-16129802-16128892-145698325-49176370-16131247-16131503-16131504-90111510-90111509-16130835-16128630-16128631-16131104-16132013-16129084-16131611-16131612-16130515-16130516-49176247-145698223-16128332-16128723-16130703-49176416-16131658-16131247-16130885-16130886-16128195-16129018-16128196-16129017-90111385-16130036-90111410-16130946-16130945-94541107-16131411-16130272-16130659-16130660-16129324-16130147-16130885-16130886-16131904-16128223-16128224-16128373-226524702-16131384-90111431-16131104-16130457-16130458-162135910-16129978-90111537-16129981-16128870-90111267-90111154-16128602-16128650-16131958-90111708-90111603-16131857-90111645-16130649-16131760-16130391-16130577-16129801-16129802-90111410-16128695-16129950-16129623-16130875-16131458-16131104-16131202-16131203-16128770-49176356-16128605-16130887-90111643-16130146-16131871-16131034-16131034-16129863-16130734-162135912-16132002-16131596-16131295-16131296-90111387-16130887-16128552-49176247-226524711-16130309-90111426-16131238-16127999-16129486-16131881-16131947-90111167-16130519-90111123-16128007-16128008-16128384-16128383-16131571-16132003-16132068-90111685-16131916-16128548-16131078

CRS76 16128650-16128575-16128576-16132030-16131446-16131445-16130152-16128291-94541127-16131473-16128675-16129049-16128674-94541101-16130123-16131458-16130153-145698258-90111274-16130930-90111528-16129087-16129367-90111303-16132081-16128631-16128630-16131959-226524700-16128007-16129342-145698342-16128008-16129341-16130670-16128004-16131153-90111612-16131420-16129604-226524762-90111246-16129265-90111123-16130990-16131427-16131777-16131776-226524739-16130371-16129407-226524714-16129691-90111203-90111269-16129382-16131244-16131698-16131699-16130513-16128054-90111288-16129485-16128527-16130103-16131763-16131843-16131742-16131742-16131742-16131894-16130477-16130653

CRS77 16131626-16128711-16129090-16129089-16130426-16131161-16130477-49176374-49176286-90111514-90111262-16130512-16128493-90111268-16129390-16131445-16131446-16129312-16131119-16129029-90111209-16131227-16131226-16130067-16127995-16129622-16128879-16128462-16128897-16131365-16131279-16131364-16128459-16128473-16130946-16128474-16130945-49176119-90111295-16131869-16131063-16130602-90111718-16131779-16130620-16131706-16130699-16131895-16131896-16131440-16131307-16128681-16131308-145698322-16128398-90111144-16131308-90111129-16128611-16131307-90111277-16129413-16130430-16130981-16131892-94541107-90111375-16129714-16129715-16131611-16131612-226524741-16130643-16130208-226524734-90111468-16130737-16129411-16129410-16131314-49176311-16131545-16129210-145698250-16131262-16130286-16128291-16129149-16129150-16130012-16131145-94541114-16129780-90111325-16130296-16129713-16130297-16130984-16130900-16130901-90111332-90111732-16129743-90111731-16130248-16131561-16130110-16129157-16129158-16130619-16130739-16131830-16132087-16131582-16131583-16131059-90111459-145698236-16130170-16130607-226524743-90111460-16128373-90111303-16131742-16131742-16129778-16128026-16128462-16129100-16130341-16129759-16129598-49176129-16130740-145698248-16129182-90111405-16132199-16131881-16129670-16130815-145698341-16129495-16129496-16129581-16130651-90111250-16130132-49176368-90111133-16130652-162135903-16129676-49176138-16130022-16130882-16129592-90111098-16128924-16128584-16128779-16130050-90111326-16130230-16130231-16129140-16130198-49176446-16131787-90111503-16129198-16128564-49176035-16129699-16129851-16130082-16130093-16129395-16128504-94541092-16129701-16130152-16128724-16130797-16130349-90111303-16130796-16129121-90111486

CRS78 16128447-16128448-16130945-16130946-16128396-16128552-16128447-16128448-16128026-145698270-16129616-16131331-16128105-16128106-16131598-90111134-90111135-90111700-16129004-16130818-16132041-16132042-16128417-49176489-90111098-16130108-16128167-16131148-16128855-16129466-16131312-16130815-16131172-16128105-16128106-90111702-90111703-16130309-90111426-16130208-226524734-90111190-90111544-16129452-16129376-16128468-16128469-90111716-16129516-16129517-16130110-16130504-16130505-16131486-90111385-16130036-16131846-16129675-90111307-49176127-16129848-16128928-16128929-16128626-16128625-16128585-16130540-16131074-16131734-16131735-16128194-16129804-16130278-16130631-226524743-16128615-16131794-90111726-16128188-16130443-90111641-16129849-90111622-16131490-49176087-16128844-16130907-16129308-16129455-16128478-16129796-90111576-16131926-16128471-16130580-16130581-49176311-16128920-90111244-90111245-16129520-16128001-16128002-16129420-16129851-16130522-90111465-16129390-16128302-16128303-16128851-16129558-49176119-16129724-16129495-16129496-90111495-16131884-16129604-16131885-145698318-16130990-16128537-16128537-90111155-16130583-16130078-16130079-

49176442-16131747-16128604-16128321-16128114-16130666-16130844-16132114-16132115-16131143-16128893-49176415-16132082-16132083-16128610-16129078-16129079-16129184-16128386-16131904-16129848-16132098-16129847-16130119-345452721-90111739-16132176-16129557-16130798-16128060-16130707-16130546-16129736-16129737-90111308-16131101-16131843-16129852-16131078-16130026-90111383-16128809-16128810-16129739-16128718-16129202-16131639-49176416-16131777-16131658-16131776-16128675-16128203-16128674-94541101-16131824-16128119-16128120-16131584-16131365-16131364-16129770-16129771-16128961-16128962-16128966-16128967-16129171-288551665-94541130-16130740-145698344-90111552-16130522-90111465-16129089-16129090-16130070-16130071-90111713-16128233-16128749-16128458-145698322-16128291-16130324-16130325-16131299-16131300-16128552-145698258-90111274-16131368-49176416-16131658-16131937-16131257

CRS79 16130632-16130633-16130620-16129072-16128548-16128537

CRS80 94541112-16128576-16128543-145698229-16128576-145698229-16128543-16132188-94541134-16132115-16132114-16132188-94541134-16130093

CRS81 16128224-16128223-16130507-16130506-90111220-16131693-16131692-16131966-16131967-16129173-16129172-16130300-16130301-16130183-16129405-90111394-90111395-16130929-16131710-162135895-16128459-16129495-16129496-16130966-16130965-16132204-16132205-16128462-16129388-16129224-16129387-16132041-16132042-16130898-90111131-16129160-90111370-90111112-16128290-16132056-16132057-16128422-16130225-16131513-16128147-16128148-16129743-16131166-90111332-49176336-16130403-90111443-16129927-221800780-16132197-16132198-16131392-145698322-16128279-16129805-16130158

CRS82 16129020-16129719-16129720-16130022-16131023-16131024-16131542-49176392-49176393-16128843-16128844-16129879-16130300-16130301-49176293-90111622-16131490-16129919-90111520-16131134-16131134-16128650-16130715-49176414-90111672-16131939-16131949-16128515-94541092-16129395-90111544-16131732-16128654-16128655-162135896-16128765-16131063-16131119-16131692-16131693-16131862-16130796-16130797-16131126-16131127-16131392-145698322-16128855-16128856-16130108-16131049-16131050-16131076-16131077-90111679-16131873-16132114-16132115-16130391-16131416-16131903-16128279-16129179-16129180-16130113-16131366-16131367-16128110-16128111-16128527-16128564-16128656-16128897-16128904-16129242-16129572-16129805-16129806-90111407-16130186-16130300-16130301-16130512-345452722-49176263-16130735-90111516-49176293-90111552-16131330-16131470-16131934-16131935-16130091-16130092-16128780-16129875-16129876-90111407-16130186-16130368-16130512-16130894-90111544-90111163-16129284-16129511-90111321-16129759-16130666-90111568-221800782-162135916-90111705-16130929-16131596-16131609-145698336-90111677-90111100-16128208-16128575-16130441-16130630-16130825-16128034-16129013-16129626-16129652-16129653-90111326-16130897-16131294-16131849

CRS83 16130395-16129739-16129863-90111083-145698243-145698244-16129751-16128723-90111497-226524700-16130445-90111451-94541134-16132188-16130168-16131747-49176442-16129863-90111581-16131391-16131390-16128955-16128956-16129244-16129700-90111550-16128815-16128816-16131262-16128138-90111389-171701684-16131849-16128049-16128048-90111665-16130147-16129301-16131653-226524748-16131779-90111276-145698306-16129216-16129217-226524704-90111174-226524711-16128265-90111631-16132038-90111707-16131535-16130205-90111413-16131247-94541107-16131445-16131446-16128012-49175991-16131653-90111622-16131490-16128829-16131942-16129652-16129653-16129876-16129875-90111631-16131535-16131935-16131934-16130844

CRS84 308209621-90111083-16128626-16128625-16129826-90111286-16128417-16128417-90111563-16128264-16129664-16130633-16130632-90111622-16131490-90111183-16131200-16130651-145698233-16128737-16129583-16129312-16130885-16130886-16128674-94541101-16128675-16128007-16128008-16130306-16131879-145698331-16131626-16129380-49176107-16129379-16130814-90111444-16131970-49176326-90111561-16131795-16131796-16129959-90111372-16131935-90111407-16130186-16131161-16128681-16131011-162135908-16129123-16128924-16131388-16129428-16129826-16129736-16129737-16128084-16131653-16129411-16129410-16128239-16128241-16132186-16130751-16130530-16129419-16128988-16128473-16128474-16130939-90111695-16129216-16129217-90111665-16130175-16130176-16131427-16131970-16129691-16128925-16128926-16128373-16129072-16132134-16129631-16129463-49176207-49176343-16131871-16129981-345452718-345452719-90111214-16129075-16128513-90111147-49176458-16128871-16128547-16131049-16131050-16130751-16131217-16129419-16128795-16128796-16130079-16130078-16129018-16129017-90111679-16131873-16129852-16128650-16130657-16130918-90111154-16128602

CRS85 16130049-16130504-16130505-90111680-90111154-16128602-16130584-90111665-16131049-16131050-16128258-16130667-90111361-16131740-16131896-16131895-16128105-16128106-16131780-90111154-16131306-16128602-90111305-16128255-90111701-16132012-16128515-16130002-16128255-16130506-16130507-16130010-16130011-16130882-16128611-16128828-90111685-16131916-90111214-16129075-16129626-16128105-16128106-90111702-90111703-16130002-16131467-16132068-16131126-90111277-49176329-16129413-16130244-16131117-16131202-16131203

CRS86 16130587-16130587-16130652-16131548-16128318-16128318-16128318-16130652-16129304-16128069-16128654-16128655-16130520-16130512-16130577

CRS87 16130828-16131330-16131331-16130281-16131330-16131331-16129693-16129694-16129804-16129776-226524730-90111381-16129249-16131775-16130591-16131552-145698247-16128018-16128017-16131359-16128874-90111084-49176035-16128564-16131780-90111316-16129934-145698339-16131856-16132089-16132090-16130424-90111448-16132134-16131328-226524700-16129545-16129637-16130262-16128194-16129545-16130433-16130434-16131382-16131383-16130079-16130078-16131836-49176480-49176479-16128870-16130657-145698239-16128935-16131959-16131903-16129167-16129168-16130591-16131392-16131744-16131745-16128986-16128046-16128357-16128990-16129960-16130591-49176391-16130143-16128898-226524706-16129620-16130594-16131034-16128899-16128993-49176133-

90111335-16129754-90111557-16131596-145698274-16128458-145698322-16128593-16128779-16129182-49176479-145698248-90111429-49176480-16128519-90111677-16128550-16129652-16129653-16128099-16129171-16129087-16130428-16130429-16131850-90111267-16131890-16129211-16131148-16130432-16130236-16130237-90111337-16131751-16128111-16130064-16130065-16130671-16130672-90111293-145698348-16130281-16128196-16128195-16129253-16131274-16132162-16131275-16132161-90111084-16128194-90111103-16128676-16129338-16130179-49176249-90111681-90111474-16129116-90111151-16128588-16130857-16128740-90111178-16129119-90111454-16129078-16129079-16130333-16130334-16128165-16129204-16129692-16129211-16131333-16130814-16128679-90111435-16130360-226524759-16132177-16128611-16130998-16129802-16131268-16128855-16128856-16130272-90111474-16130723-16131382-16131383-16129801-90111588-16130988-16130242-16130987-16130914-49176356-16131961-16128639-16129348-16129349-16130002-145698334-16131857-90111152-16132170-16128437-49176025-16131666-16132195-16130146-16130998-16128773-226524737-16131068-16130338-16130339-16128458-16128871-16129933-16129933-16130262-16130289-226524743-16131578-16131658-90111683-16131268-16131303-157783152-16131515-16132134-16131939-145698229-16128543-16129086-90111430-16131161-16129455-16130219-16131307-16131308-145698279-16129832-16130799-16130800-16131571-90111335-16129754-16128703-16130619-16130999-16131760-16129121-226524725-90111347-145698342-16131958-16131706-145698228-16130518-16130123-16131925-90111082-16128986-16129121-16129298-16129670-16130311-16130480-90111458-145698310-49176343-16128665-16128917-49176170-16130825-16130967-16131471-16131782-16128548-16130929

CRS88 16131885-16129826-16131884-16128584-16129237-16131703-16131704-16130887-16131152-16131153-90111378-16131152-16131153-16128295-16129140-16129139-16129661-16131034-16131837-90111707-16131653-16132038-16128807-90111175-16129969-16131202-16131203-226524719-16128766-16128767-16128194-221800779-16130224-16128832-16132068-90111563-16131852-16131034-226524753-90111739-16130667-16132176-90111444-16128515-16128681-16129419-16131356-16128042-16129545-16130430-16128966-16128967-145698258-90111378-90111274-90111083-90111295-145698252-16130956-16130957-90111677-16131458-345452718-345452719-16128722-16128721-16131259-16131740-145698344-16129927-16130583-16129583-16130049-16131053-90111612-16131420-16131365-16131364-16132176-90111739-16129630-145698299-16130396-90111695-16129861-16129862-16132220-226524713-16131455-16131843-16129245-94541109-16131456-90111679-16131873-16128548-16131388-16129165-16130430-16130751-16131247-16131247-90111617-16131395-16129455-16129237-90111631-16131535-90111079-16130281-90111182-16128845-16130745-145698300-16130583-16128611-16131840-16131639-16128193-16129429-16129984-16128192-16131823-16131373-90111122-16129563-16130875-145698344-16128213-16128212-16128018-16130796-145698309-16128017-16128988-16130797-16130306-226524749-16128203-16129185-16129186-16131284-90111383-145698336-16130026-145698233-345452718-345452719-90111444-221800782-16131247-16128737-16129676-49176138-16129826-16130584-16130166-16130167-16129020-16129049-16129414-16129927-90111389-90111489-16130682-221800782-16130667-16128749-16129146-16128748-16128540-145698256-90111310-16129601-16128528-16130319-16129119-16131021-16128922-90111277-16128923-16128968-16129413-16129179-16129180-16129637-16130231-90111622-16129950-16130147-16130230-16131490-16129375-16131948-90111363-16131776-16131777-16128445-16131941-90111603-16129545-16130887-16128203-90111561-90111678-16131868-145698331-16131626-16128134-16129373-145698255-16129713-90111325-16129458-16131079-16130331-16131703-16131704-16131795-16131796-16129935-16129913-90111497-49176301-145698336-16128527-16128685-16129919-16130049-16130584-16131314-16131653-16128684-16130049-90111552-16131611-16131612-16129460-16129901-16131873-16128241-16129455-16131217-90111679-16131970-16131740-16129702-16129338-16130604-90111563-16128425-16129863-16131391-16132120-16128133-16128266-16128748-16129308-16129309-16131390-16128407-16128408-16128528-16129017-16129018-16131869-16128573-16128574-16128828-16129187-16130999-16131740-16128989-49176356-16128396-16128703-16129455-16130148-16131216-90111629-16132041-16132042-16132112-308209621-16131903-90111389-16131384-16128032-16128989-16129349-16129598-49176129-345452721-16130734-16128462-90111165-16129348-16131318-16131780-16128694-16131479-16129981-16131848-16131847-16129747-90111334-16130639-16130640-16129041-16128925-16129126-16128926-16129771-16129127-16128548-16129517-16129516-16130584-16129303-90111380-90111253-16130008-90111389-16130026-16131152-90111383-16131153-145698336-145698342-16128864-16129616-16128865-145698270-16129702-16131148-145698336-145698338-16132060-90111670-16128430-90111136-16128162-16128459-16129057-16129969-49176373-16131442-16129539-16128946-16132204-16132205-90111123-16129244-16131456-16131935-49176479-16129697-16131200-49176480-90111741-16128203-16131455-90111701-16130745-16130939-16132012-145698236-16129211-16131772-16131793-90111681-16131887-90111307-49176127-16129592-16129846-16130835-16128993-16130602-16131385-16130334-16131382-16128548-16129846-226524732-16130333-16131383-90111474-16128576-16129545-90111416-16130400-16130734-16131947-90111603-16130278-16130835-90111214-16129613-16130306-145698342-49176480-16128789-16129075-16129612-49176479-16128034-90111081-90111244-90111081-16128528-90111245-16128143-90111352-16129828-16130757-145698336-90111155-49176170-16131954-16128548-16129562-16128034-16129284-16130169-49176329-90111708-16129724-16131117-16130503-16129752-90111462-16128855-16128515-16129495-16130524-16129496-16130771-90111581-16129331-90111261-16128143-226524724-16131765-16130558-16128986-145698310-16130967-16128002-16128001-16131704-16129051-145698334-90111211-16128302-16128303-16128695-16131498-16128012-16128013-90111083-16129020-16130462-16130463-16130689-16131030-16131177-16131448-16131456-16131970-16129219-90111238-16129561-16131455-16130829-16129146-49176458-162135899-16130230-90111643-16130231-16129395-94541092-16130319-16130756-16131411-16128586-16129157-16129158-16129721-145698342-16128718-16129185-16130518-16129186-16128527-16128747-16128605-90111180-16129971-226524734-16130745-90111562-16128594-90111186-16129242-16130445-16131202-16128570-16128602-16129428-16130208-90111451-

16131133-16131203-16131318-16131318-90111617-16131529-90111079-16131903-16129119-162135900-16130272-16130512-16130594-16131284-16129121-90111234-16129863-16130153-16131348-16129927-90111193-16129076-16130208-226524734-226524753-90111404-145698270-16129616-16129778-145698252-16129759-16128705-16128706-16130844-16131942-49176071-16128979-16128980-16128981-90111253-16130835-16131023-90111557-49176329-16131117-16131153-16131377-16131591-16129122-16129255-16129303-90111268-16131024-16131152-16131545-16132186-90111307-49176127-90111462-16130503-16131927-16131467-16130734-16130990-16128310-16129755-16130213-16129861-16131747-49176442-16128375-16130428-16128547-16129852-16130429-16131653-90111383-16130026-16130492-16128796-16128795-226524756-16131910-16130739-16129675-16130742-16130743-16132120-16128311-16128447-16128448-16128481-16128966-16128967-16129101-16129661-16129852-16131155-16131224-16129065-49176400-49176077-16129458-90111646-16130175-16128008-16130176-16132026-90111603-16129699-16131846-16132117-16129203-16130724-16132049-16132048

CRS89 16130522-90111465-16129856-16129857-16130522-90111465-16130522-90111465

CRS90 16132062-90111741-16130245-16129698-90111273-16129004-16130893-16128642-16131656-16129771-16128357-16128990-16128373-16128414-16130348-16128091-226524746-16131979-90111694-16129116-16130163-16130651-16130545-16130388-16130389-16130724-16130245-16129406-16131568-90111747-16132208-90111380-16130008-90111438-16129830-226524762-16130195-90111165-16129563-49176423-16130720-49176422-90111486-16131780-16128543-145698229-94541112-16130888-90111524-16130807-16128415-16131129-16130102-16130499-16131926-16130843-90111741-16130091-16130092-16129084-90111683-16131905-90111097-145698316-90111257-16131912-90111211-16129051-308209621-16128820-90111097-16131519-90111628-90111458-90111631-16131535-16128166-16131640-90111680-16131792-16130217-16131148-90111680-90111114-90111267-16129443-296044035-16132021-16129406-49176391-16132150-90111609-16128105-16130067-16131852-16128117-49176446-90111088-16129891-90111360-16131787-16130230-16130231-16128874-16131920-16129197-16129914-90111581-16130075-16128550-16129982-90111678-16131868-16128068-90111419-16130266-16128607-90111348-16129819-16130075-16129216-16129217-16130143-16130846-16128426-16130929-16129026-16128568-16131803-16131804-16130652-16130094-16129768-16130251-16130579-16130213-16130271-16130137-90111681-16132060-16130452-16131921-16130012-16131731-16130825-16131321-16131513-16128055-16128055-16128055-16128982-145698339-16131856-16129701-16131912-16132108-16128362-16128361-16128506-90111416-16129254-16131659-90111645-16130984-16128373-90111176-16132113-16128937-16128179-16129253-16128097-16128096-16129658-16129659-16128506-16131334-16128039-16128101-162135896-16128765-16130444-145698312-90111424-16131545-16129482-16129616-145698270-16130124-16130125-16130137-16131703-16131704-16128506-16130720-90111379-16130007-90111185-16128873-94541090-16128366-16129170-16130271-90111564-16128461-16128460-16128315-16128316-16130579-16132113-16129375-145698298-16129331-16129172-16129173-49176087-49176088-49176089-49176368-16128970-16129252-16129735-16130149-90111428-90111189-16130198-16131954-145698255-16129373-16131799-16132087-16129655-16130540-16131098-16130524-16130699-16128681-16128596-145698329-16131065-16130937-90111530-16128681-16131823-16130745-16131129-49176448-16128567-16128568-16130075-90111081-16131063-16128191-16129680-16129681-16131920-145698226-16128721-145698262-90111291-16131678-16128722-16129419-16131677-16129493-16130495-90111159-16129633-16131324-16131950-90111544-16129565-145698268-16129255-16131927-16129252-90111289-162135903-16128829-16129488-16131542-16128794-16130545-16128681-90111168-16129598-49176129-16130442-16130494-16130496-16130498-16130520-16131541-16128828-16130141-16128084-90111683-16130217-16130289-16130684-226524742-16130196-16130141-16130892-16131058-90111633-16128671-16130135-16130137-90111493-16130727-16130868-16130961-16128427-16130960-16129774-226524755-90111189-16131328-16131665-16132080-16129999-16131271-16128519-16130638-16130651-90111158-16128628-145698293-16130384-16128610-16129999-16131941-16129525

CRS91 16131279-16131364-16131365-16129122-90111665-16131121-16131760-16129349-16129348-16128250-16132105-16131934-16131935-16128723-16132060-16130049-16130520-94541134-16132188-16128586-16130505-16130504-16128264-16130049-16129172-16129173-16130429-16130643-16130428-226524741-16128367-16129631-16131119-16128570-90111238-16129090-16132201-16129089-16130499-16130151-90111481-16130617-16129615-16129794-16129795-16130587-16131740-16128266-16130278-16131101-49176295-16131011-162135908-90111741-49176358-16130961-16130960-49176393-49176392-16129146-162135899-49176127-90111307-16128491-90111360-16130742-16128594-16130708-16130743-16131030-16131389-16128993-226524706-16131203-16131504-16131202-16131503-16129871-16129870-90111557-16129973-16131365-16131364-49176138-16129925-16129676-16129973-90111084-16128952-16128896-16130743-16130742-16129852-16128395-16131313-16131312-16129633-90111568-16130977-16129771-16128980-16131470-16127995-16128012-49175991-49176497-226524765-16128938-16127995-16130747-16128105-16131446-16131445-16132026-16132003-16130463-16130462-16131843-16131503-49176414-16129633-16128568-16132134-16128567-90111370-226524728-16128548-16128257-16129375-16129511-16130814-16131591-90111702-90111703-16132134-16129378-16130248-16129003-16129997-16129761-16128906-16129539-16129458-16128156-145698340-16131860-16131861-16128068-16131238-16131531-94541128-16129561-16129978-16131892-49176479-49176480-16131482-16131483-16128551-16128466-90111140-16129499-16130419-16130418-16129211-16129388-90111494-16129387-16129631-145698297-16130749-16129237-16128012-49175991-90111286-16131330-16131331-16128105-16128654-16128655-16128540-16129013-16131762-90111653-16128896-16128034-90111081-16128367-16128474-16130945-16128459-16128473-16130946-16129100-16128759-16128758-49176408-16131712-90111142-16130186-90111407-16130984-16129981-16129539-145698298-145698312-90111665-90111316-16129732-16129733-16129854-16130939-226524726-16128366-16129904-16129905-16129075-16128396-90111214-16130278-90111544-49176474-90111714-49176489-90111211-16129051-16130123-90111688-

90111440-16129140-16130867-16131881-145698341-90111384-145698260-16130988-16129433-16130987-16130175-16130176-16129927
CRS92 16129024-16130168-90111108-90111083-16129335-16129504-16128537-16129954-90111205-16129057-16131819-16131857-16128255-16131127-16128571-16128904-90111190-16130512-16129179-16129180-16130875-145698309-16128635-90111199-94541103-226524718-16129057-16129345-16128748-16128749-90111264-16129210-145698250-16129660-16129494-145698312-16129187-16130596-90111458-16128527-16129913-16129675-16129615-16132153-16131794-16128674-94541101-90111555-16128675-16128383-16128384-16131798-49176358-145698310-16132134-49176480-49176479-49176368-16131411-16129818-16131458-16129929-145698341-16131881-49176107-16129379-16129380-16128460-16128461-16131368-162135903-16131970-16131577-90111198-90111730-90111087-16128116-49176071-16128979-16130050-16129697-90111407-16130186-16128091-90111361-16130141-90111527-16131436-16131437-16128212-16128213-145698274-90111561-16131436-16131437-16129697-16131935-90111440-16131524-16131525-16131333-16128980-16128981-16130793-16128844-16129743-90111332-16130271-162135897-90111171-16128196-16128195-16130168-16128955-16128956-90111257-16130999-90111440-16130583-226524763-16129239-16129238-16129100-90111629-16131216-16131810-49176276-16130771-16128410-90111132-90111305-90111083-49176267-16128575-16131502-16131718-16130722-90111492-16131529-49176390-16128375-90111659-16131722-90111325-16129713-16131101-16131240-16130937-90111530-90111603-16129167-16129168-16128779-16129691-90111495-90111692-16131760-16129407-226524714-16129846-16130708-90111086-16128465-16131988-16130357-16131110-16128982-16131836-16131101-16131958-226524735-16130146-16131011-90111121-16128481-16129919-16129954-16130882-16132177-16131734-16131735-16128650-49176459-90111663-16129390-90111407-16128895-16130186-16131217-16128701-90111431-16128962-16128961-16130671-16130672-16130226-145698231-16128528-16128669-90111497-16128008-49176137-145698318-16132127-90111122-90111440-16129121-16128252-345452715-16128154-16128501-16128502-90111378-16131529-90111380-16130008-49176448-16131843-90111144-16130641-90111155-145698312-16131294-16129395-94541092-16128605-94541112-16129054-145698252-16130153-16131384-145698339-16131856-145698224-90111561-16129933-16128944-16131463-16128537-16128676-16131464-16131325-16132082-16132083-16128703-16129158-16129157-226524732-16132135-16130755-16130757-16128571-16129884-16129885-16130278-16130620-16130491-16128490-16130939-16131455-16131456-16132063-308209623-90111123-16129295-94541101-16128675-16131366-16131367-16130187-16131217-16131808-16130183-145698288-16131153-16130520-16128026-16129646-16129714-16129715-90111677-16132082-16132083-90111250-90111355-16128915-16128914-16130371-90111262-16128554-16128555-16129631-16130771-49176276-16129633-16128535-145698297-16131028-16129338-16129375-16130320-16130321-145698309-16131596-16129743-16131752-90111332-49176443-16130049-16130747-16130583-90111440-16131736-16130270-16128642-16131295-16131408-16131296-16131407-16129952-162135904-16128279-16128892-16128950-16129245-16129715-16129714-16129101-16129102-16130368-16130591-16129017-16129018-90111497-90111295-16128568-16128567-16129919-16128671-16128437-49176025-16130345-16130344-16128567-90111154-16128602-90111184-16130543-16129076-94541124-16129003-16130768-16130103-90111643-16129615-16129737-16129884-16129885-16131591-16129078-16129079-16128109-16129185-16130798-16131752-90111131-16131653-49176443-16130534-16130533-16128950-90111603-16128920-49176177-16130225-16128239-16129428-16131011-162135908-16129482-90111591-16129861-90111171-162135897-90111209-16129029-16131833-90111674-16128364-90111125-49176018-90111305-16128937-16130219-16131598-16128988-49176329-16131117-16128650-16129072-16131279-16131848-16131847-16129614-16130163-16128778-90111631-16131535-16128528-16130687-16128165-16128425-16129851-16129622-16128311-16130445-90111451-16131709-16131242-16131243-16129559-16129363-16129937-16130763-16129100-16131879-16130939-16129721-16128367-16131119-345452725-16130297-16130296-16130147-90111182-90111320-16128845-90111575-16130225-16128241-16128239-16128519-16128417-16128906-162135916-90111705-16130918-145698291-16129804-226524753-16130583-16130912-145698248-16131216-16131736-16129182-16130755-16129494-16130546-16130844-90111250-16128763-16130740-16131852-90111614-16131779-90111114-16130606-16130671-16130672-16129101-16129101-16129102-16129102-16130147-90111468-49176414-16131488-16131757-145698233-16129511-16128737-16130153-16131846-49176407-16128555-16128554-16129575-16130807-16130049-145698279-16128343-16128604-16130924-16130923-16132197-16132198-308209621-90111389-16129660-226524751-16131506-16131542-162135906-16128920-16128635-16128694-16131593-16130207-16130184-16131506-16130828-49176193-49176293-16130100-90111381-226524729-226524730-16129988-16131314-16128425-16128165-16128654-90111256-16128655-90111255-16131839-16131838-16128478-16130603-16128989-16128572-16131221-345452721-16132060-16130049-16131839-16131838-16130316-16128025-16128266-90111081-90111266-16128034-16130630-16132158-90111114-94541127-16131473-16131830-16128034-90111081-16130744-90111237-16129213-16128583-16130608-16128582-49176260-90111242-16129422-94541110-90111278-16129076-90111147-16129899-90111359-16130492-16129101-16130013-16129102-16130543-16128904-90111190-16131524-16131525-145698292-16130163-90111421-16131119-90111690-90111691-16128779-90111705-162135916-90111291-16129493-16129036-16129801-145698341-16129035-16129802-16130962-16131200-16131881
CRS93 16128547-16129455-16130300-16130350-16130430-226524711-16129700-90111384-16128585-16130172-16130667-16130745-16131104-16131316-16131653-16130010-16130011-16132177-16130430-16130751-145698341-49176329-90111743-16131117-16131849-16130824-16129100-162135894-16130049-16131840-90111685-16131916-145698325-49176370-90111280-90111389-162135896-16128765-16129093-16129185-16129186-16129338-16131958-16132188-94541134-16128605-16129185-16128366-16128367-16132183-16128366-16128367-16128676-16131779-16128723-162135894-16130049-16131840-90111685-16131916-90111079-16129100-162135899-16129146-16130755-16131954-16128590-16128591-90111186-16129619-16129975-16131247-16131318-16129202-16129003-145698258-90111274-

90111123-16129004-16130885-16130886-145698309-16128053-16129165-90111288-16130168-16129485-226524719-90111685-16131916
CRS94 221800782-16130082-16131859-16129252-90111163-49176045-16131757-16130143-16130914-16129310-16129569-16128703-90111176-16131477-16129197-16128414-16131230-16131231-16130070-16130071-16132150-16131479-16128568-16128459-16131461-90111417-16130143-90111637-145698339-16131856-16130495-226524743-16129245-16128244-16130444-90111105-16132148-16128900-16129231-16129087-90111605-90111606-16130242-16128795-16128796-90111522-16130867-16128711-16130088-90111403-16128225-16130619-90111611-16130630-16130496-16128095-16132108-16128965-16130013-16130650-16129642-16131498-16129988-16130132-226524735-16131508-16131063-16128478-16128022-16128032-16128209-16128214-16128308-16128308-90111117-345452717-94541100-16128650-16128665-16128701-90111173-16128828-16128984-16129013-16129041-16129695-49176170-16129985-16129985-16129999-16130069-16130082-16130170-90111452-16130719-16130825-16130859-90111523-16130965-16130966-16130978-16131134-16131134-49176343-16131323-16131325-16131415-16131471-90111642-16131593-16131595-16131742-16131742-16131742-16131755-16131775-16131842-16131842-16131859-16131863-16131880-16131894-16131922-16131933-16131933-16132002-49176475-16132059-16132081-16132145-16131226-16131227-90111176-16130838-16131782-16130831-16131934-94541118-90111399-90111247-16128340-90111458-16128270-16130347-16129047-90111210-16130519-226524764-16132172-16131851-16131850-16129919-90111468-16131471-145698267-16129230-16130577-16131912-90111181-16128308-16128308-16128308-90111117-345452717-16128701-16128828-16130170-16130170-16130170-90111452-16130719-16130966-16130978-90111537-49176343-16131742-16131742-16131880-16131894-16131922-16132002-16132059-16128022-94541100-16128650-16128665-16128754-145698236-16129041-49176170-16129999-16130069-16130825-90111523-16130965-16131415-16131471-90111642-16131755-16131775-16131782-16131842-16131863-16131863-16131933-16131933-16131970-49176475-16132145-16132199-16129688-16129249-16131593-16130619-16129700-16130488-90111106-162135895-345452715-16130804-16128252-16128575-16130687-49176437-16128979-49176071-16130703-90111382-16130735-296044035-16132021-16128754-16132080-16129184-90111519-16128763-16130653-16128902-16131519-90111628-90111261-16131323-90111693-16132110-90111368-90111168-49176302-16130311-16128540-16131416-16128385-49176390-90111094-16131412-16130213-16130257-90111108-16130012-16129113-90111554-16131072-16131111-16129626-90111716-16129633-16130868-49176459-16130132-16130670-90111573-49176308-16131906-94541100-16130825-16130082-16131806-16128156
CRS95 145698269-90111312-16128723-90111735-145698229-16128543-16130428-16130429-16129640-16128809-16128810-90111612-16131420-16128904-90111190-16128573-16130129-16128574-16130130-16130745-94541112-16130130-16128576-16128203-16129975-16129324-16129335-16129504-16131776-16131777-16130340-16130266-90111419-16129168-16129167-16128192-16128193-16130667-16131294-16129101-90111277-16129413-16130400-226524724-16129204-49176329-16131117-16130703-16130967-145698310-90111378-16132214-16132213-16129558-16129559-16131202-16131503-16131203-16131504-16128723-16128096-16128097
CRS96 16130064-16130065-16132134-16128586-16129630-16130248-16130278-16131101-16132134-49176497-226524765-16130663-90111237-16129213-16129455-16130956-16130957-16130720-16129103-16131202-16131203-16128528-16130735-16128026-16128452-16130093-16128451-16130301-16128896-16130751-16130757-90111173-16131840-49176489-16129630-16131314-16131342-16131343-16131659-226524741-16130643-16131947-49176422-49176423-16128567-16128568-16129003-16130049-16130520-16128655-145698336-16128012-49175991-16128654-90111552-16128462-16128264-16129852-16132200-16128034-16130186-16130887-90111081-90111186-16129123-90111407-90111609-16132026-145698279-16129855-49176127-90111307-16129666-16129848-16132089-16132090-16128537-16129675-16129308-16132040-90111353-16130591-226524748-16128614-49176480-49176479-16128659-157783150-16129013-16129700-16130894-16131768-16128706-16128705-145698291-16130707-16130720-16129452-16130150-49176425-16131680-16132002-162135908-16130649-16131011-16128110-16128111-16128968-49176418-16128605-16128488-16128489-90111294-16130230-16130231-16131440-16128986-90111495-90111326-16129203-16130231-16128445-16130230-16130430-90111255-90111256-16130010-16130011-16131407-16131408-16129310-90111642
CRS97 16131680-49176425-90111261-16131323-16128711-16128763-90111153-16130577-90111637-16128900-16129620-49176133-16128946-16132040-16131593-49176417-16131666-16129010-16130504-16130505-145698321-16130624-16129778-16130103-16131137-345452716-16128192-16128193-16130067-16129751-16130545-90111558-16128308-16128308-16128308-90111117-345452717-16128701-16128828-16130170-16130170-16130170-90111452-16130719-16130966-16130978-49176343-16131742-16131742-16131880-16131894-16131922-16132002-16132059-16128022-94541100-16128650-16128665-16128754-145698236-16129041-49176170-16129999-16130069-16130825-90111523-16130965-16131415-16131471-90111642-16131755-16131775-16131782-16131842-16131863-16131863-16131933-16131933-16131970-49176475-16132145-16132199-16131815-16129482-49176282-16130630-16131508-90111741-16129247-16130289-90111573-16130577-16131549-226524758
CRS98 16131197-16130868-16128188-16129045-16131656-16128061-16132197-16132198-90111508-16131919-16130732-145698258-90111274-16131601-16131578-16129116-16128989-49176390-16131799-90111509-90111510-16128811-162135905-16131919-16128938-16129249-16128095-16130846-90111339-16129781-90111582-90111493-90111672-16128596-16128191-16130131-16131568-49176390-16131411-49176368-16128668-16130270-16131941-16129057-16128794-16128550-16128663-16128662-16128674-16131079-16129265-90111246-90111177-90111430-49176448-16128761-16130732-16131978-90111354-16129237-16130219-16132144-16128513-16130626-16130195-16131229-90111332-16129743-16130897-226524732-90111428-16129702-145698310-49176448-16132196-16130672-16130671-16131893-16130012-16129261-16130899-90111168-145698310-16129254-16130253-90111082-16132150-16129830-90111558-90111493-90111488-16129632-16131842-16131947-16131947-90111361-90111520-16131926-16130625-16129914-16128439-16128440-16129700-

16129363-16129937-16130763-16131548-16131924-16131530-16129499-16128095-16131321-16131263-16132144-90111361-16131508-90111428-90111429-49176475-16130892-16129700-16129721-90111520-16129141-308209620-16131961-90111430

CRS99 16129151-90111354-16129152-16130104-90111550-226524750-16128765-16130183-162135896-16131744-16131263-16131745

CRS100 16129759-16131436-16131437-16131934-16131935-90111106-16130771-16132188-94541134-16131768-16129058-16130251-90111718-16130169-16131436-16131437-345452719-16130738-16130780-16131836-16131436-16131437-90111454-16130439-16130738-16129862-90111631-16131535-90111282-145698288-16130755-16129441-16130187

GFR1 16130424-16128507-16129193-16130433-16130401-16129242

GFR2 16130499-16128102-16129694-16128898-162135898

GFR3 16130403-16128025-16130742-16131307-16131845-16130807-16131796

GFR4 90111645-16131604-16131639-16132000-16130443-16129581

GFR5 16131596-16131593-16128384-16131365-16131846-16130887-16131341-16128001

GFR6 16131063-16132076-16131238-16131127-16131795-16128026-16130858-16129715-16129152

GFR7 16131483-16131757-16129733-16130828-16131754-16129632-16131851-16129970-16130815-16132212-16128723-16128109-16128107-16130826-16130320-16129807-16128493

GFR8 16129620-16129238-16130937-16128398-90111575-16130151-16128743-16128742

GFR9 49176207-16130212-145698291-16130388

GFR10 16132002-16128510-16131955-16130330-16128187-16129828-16130052-16129819-16130604-16129595-16131431

GFR11 16131597-16131598-90111555-16128366-16129155-16128807-49176077-16128142-16131072-16128089

GFR12 16128421-16128422-16128424-16128425-16128426

GFR13 16131057-226524751-16131059-16128162-16128167-16128163-16128165-16131121

GFR14 16128371-16128228-16128980-16128897-16131103-16128258-16130722

GFR15 16128127-16128124-16128138-16128410-16128096-16129766

GFR16 16131813-16131815-16131816-16129675-16129670-16131076-16130539-16130494-16131200-16130530-16131189-16129167-16129672-16128017

GFR17 16130640-16130607-16131869-16129235-16131665-16128092

GFR18 16131884-16128747-16131704-16131506-16131885-90111516-16130862-16131047-16130505-16129814-16129703-16130337-16129077-16128682

GFR19 16130963-16130961-16130962-16131177

GFR20 16131632-16128068-90111084-16130814-16128656-16129960

GFR21 16130659-16130671-16130340-16131678-16131777-16131779-16131839

GFR22 16131570-16130166-16131773-16129718-16130930-16130915-16130817-16130587-16129227-16130266-16128383-16128202

GFR23 16131765-16131300-16130518-16131299

GFR24 90111581-226524719-16128874-49176336-16128373-16128576-16131880-16130522-16130744-16129660-16128722

GFR25 16131830-16128888-16128848-16130735-16129301-16132183-16128451-90111205-16129568-16129795-16130169

GFR26 16131755-16130350-16132064-16131891-16131894-16128981-94541101-16131230-16129211-16130227-16128738-16128462-16128449

GFR27 16128851-16128458-16131817

GFR28 16130838-16128114-145698307

GFR29 16128703-16128701-16131436-16131916-16130389-16128002-16131384-16131028-16131019-16131280-16129768-16129569-16129658

GFR30 16131768-16130199-16131698-16131808-16131319-16128795-49176426-90111515-16130184-16130167-16128749-16128611-16128645-16128003

GFR31 16128575-16128568-16131666-16128720-16128573-16129855-16130324-16130936-16131284-16129303

GFR32 16128161-16128164-16128018-16128042-145698236

GFR33 16129871-16129870-16129811

GFR34 16131068-16131069-90111553-16132189

GFR35 16128871-16128869-16129805-16129197-226524704-16131435-16129982-16132197

GFR36 16129204-16129205-16129213-16128148-16131903-16129683

GFR37 16131215-16128780-16129852-16129794

GFR38 16132060-16132059-16132041

GFR39 16128057-16128055-16128058-16130707-16130703

GFR40 16129230-16129776-16131848-16129049

GFR41 16130533-16128008-16130852-16131145

GFR42 16128778-16128779-16131524-16131753-16128731-49176401

GFR43 16131574-16131571-16131609

GFR44 16130730-16130725-16131420-16130096-16130008-16130605-16129818-16128439

GFR45 16128715-16128711-90111166-16128718

GFR46 90111212-16129058-16129057-16131143

GFR47 16131513-90111624-16131836-16132067

GFR48 16130434-16128407-16128513-157783150
GFR49 16131860-16131859-16131932-16131330
GFR50 16128879-16128877-16130732-16132193-16130123
GFR51 16132055-90111557-16128615-16130445-16128074-90111157
GFR52 16129035-145698279-16129034
GFR53 16131117-16131949-16129667-16130093-16128143-16132114-16132112-16131804-16130959-16130984-16131935-16131549-16131400-16131963-16129324-16128595-16128040
GFR54 16131416-16131415-16131582-16131170
GFR55 16129017-16128208-16131039-16129995-16129808-16129985
GFR56 16131995-16131994-16131989-16131999
GFR57 16129172-16129170-16129173
GFR58 16130248-16130251-16130255-16130311
GFR59 16129065-16128567-16128773-16128570-16129149-16128152-16128556
GFR60 16128857-16128855-16128915
GFR61 16132082-16132081-16128147
GFR62 16131306-49176351-16131126-16131236
GFR63 16131115-90111559-16131172
GFR64 16131776-16131677-16131850-16128650-16131612-16130906-16127995-90111288-16128895
GFR65 16131337-16131504-16131520
GFR66 16128637-16128638-16128192-90111355-49176333-16129752-16128386
GFR67 16131905-16131735-16131895-16131620
GFR68 16129553-16129554-16129561
GFR69 49176450-16128459-16129934
GFR70 16130339-16129236-16130347-16129341-16129721-16131389
GFR71 16130577-16130591-16130824-16132030-16130094-16131941-16129451-16130503
GFR72 16131763-16130175-162135900
GFR73 16130953-16129488-90111182-16129249-16130597-16128463-16130599-16130121-16129388-16129842
GFR74 16131824-16128408-16130042-16131288-16129596-16128194
GFR75 90111384-16130029-16130225
GFR76 16131631-16131627-16129702-16131957-16128179
GFR77 16131228-16131226-16131242-16131275-16130498
GFR78 16129099-16128318-16129237-16131794-16130147-16128696
GFR79 16131878-16132023
GFR80 16131742-16131744-16131745-49176045-49176446
GFR81 49176156-16129783-16129720-16129762
GFR82 16129885-16129891-16130477-16129016-16131373-16132176-90111289-16129838
GFR83 16129210-16128758-16129619-16131868-16130914-16131985
GFR84 16130156-16130007-16130130-16128554
GFR85 16128899-16128912-16129969
GFR86 16132142-16132117-16131928-16129179-16131474
GFR87 16129284-16129196-16130693-16130153-16131539-90111362-16130301-16128519
GFR88 16129691-16129692-16129771-16130341
GFR89 16131967-16130960-16131806
GFR90 16131988-16131477-16132204
GFR91 16131241-16129226
GFR92 16128828-16128832-16129857-16128313-16130244-16131966-16131053-16130245-16128105
GFR93 16130843-16130861-16130796-90111661
GFR94 16129759-16129758-145698269
GFR95 16131710-16131709-16131371
GFR96 16131391-16130945-16130095
GFR97 16131336-16130531-90111653
GFR98 16128448-16128447-16128465
GFR99 16129499-16129498-16130596-16129295
GFR100 16129036-16129041-16129072
PCS1 145698223-145698261-16128430-16130643
PCS2 94541124-145698310-90111382-16129090
PCS3 145698224-145698245-16129498-90111381
PCS4 145698230-145698236-16131520-16129612-16128857-16130862
PCS5 145698233-145698255-16131671-16128765
PCS6 145698239-145698262-16128602-16129246
PCS7 145698247-16128068-16128607-16129954
PCS8 145698254-145698331-16128117-16128785

PCS9 145698256-145698300-90111460-16129380
PCS10 145698260-16128054-16128676-90111611
PCS11 145698268-145698292-162135918
PCS12 145698269-16128123-16130542-90111716
PCS13 145698279-145698288-16130540-16131341
PCS14 145698281-145698308-16129249-90111451-16129143
PCS15 145698285-145698338-16130121-16130799
PCS16 145698291-145698295-16130212-16129613-162135905
PCS17 145698301-145698303-49176235
PCS18 145698314-16128073-16132213-16131582
PCS19 145698322-16128017-16132076-16131871
PCS20 145698327-16128003-90111675-226524763
PCS21 145698334-16128001-16131836-16131916-16131593-90111325
PCS22 145698340-16128107-16129811-16128171
PCS23 157783149-16128000-16128899-16129036
PCS24 157783152-16128202-16131967-16131506
PCS25 16128002-16128026-16130684-16128528
PCS26 16128004-16128013-49176011-16131932
PCS27 16128022-16128032-16128105-16131319
PCS28 16128025-16128074-16128656-16131792
PCS29 16128034-16128148-16130779-16131047
PCS30 16128040-16128124-16129186
PCS31 16128042-16128049-16128912-16130130
PCS32 16128048-16128089-16130169-16130170
PCS33 16128053-16128057-16129644-90111688
PCS34 16128058-16128063-16129300-16128464-16128461
PCS35 16128059-16128120-16131971-16129072
PCS36 16128092-16128110-16131913-90111225
PCS37 16128096-16128102-16131806
PCS38 16128097-16128111-16128142-90111272-16128384
PCS39 16128101-16128205-16130145-16128316
PCS40 16128103-16128109-16131815-90111088
PCS41 16128106-16128119-16132002-16128779
PCS42 16128114-16128153-16128879-16130879
PCS43 16128116-16128152-90111523
PCS44 16128127-16128201-16130492-16129167-16128611
PCS45 16128133-16128213-16128428-16129744-16128465
PCS46 16128138-16128192-16130457-16128332
PCS47 16128143-16128271-16131722
PCS48 16128147-16128149-90111084-16130394
PCS49 16128154-16128165-16128715-16131833
PCS50 16128160-16128183-16128758-90111320-16131437
PCS51 16128163-16128244-16131420-16131710
PCS52 16128193-16128210-16128395-49176361
PCS53 16128194-16128321-16132090-90111230
PCS54 16128195-16128196-16132000-16128501-16131861
PCS55 16128208-16128209-16129182-16129235
PCS56 16128212-16128217-16128439-16129650
PCS57 16128214-16128290-90111539-16129818
PCS58 16128223-16128224-16128313-16131985
PCS59 16128228-16128324-16129876-16131754
PCS60 16128239-16128257-16131141-16132145
PCS61 16128241-16128318-16132112-16129214
PCS62 16128252-16128303-16128389-16129504
PCS63 16128266-16128383-16129126-49176267
PCS64 16128273-16128435-90111273-16131094
PCS65 16128281-16128452-16129616
PCS66 16128291-16128315-16131296-90111234
PCS67 16128299-16128370-16128731
PCS68 16128300-16128447-16129494-90111253
PCS69 16128310-16128443-16130095
PCS70 16128348-16128459-16132041-49176475

PCS71 16128361-16128366-16129776-90111167-16129398
PCS72 16128371-16128424-16131059-16131148
PCS73 16128378-16128390-16128408-16131838
PCS74 16128398-16128402-16128567-16131069
PCS75 16128407-16128471-16130701-16129999-16130175
PCS76 16128410-16128448-16130671
PCS77 16128417-16128588-16131609-16130515-16128851-16130330-16131166
PCS78 16128421-16128422-16128425-16128591-49176171
PCS79 16128426-16128457-16128478-16131880
PCS80 16128427-16128575-16128576-345452717
PCS81 16128437-16128462-90111336-16129150-16130125
PCS82 16128440-16128453-90111706-16130641
PCS83 16128449-16128549-16131388-16129652-16130044
PCS84 16128460-16128526-16128225-16129851
PCS85 16128463-16128637-49176479-16129752
PCS86 16128468-16128509-16128720-16131885
PCS87 16128473-16128513-16131337-16130456
PCS88 16128474-16128722-90111183-16128874
PCS89 16128481-16128586-16129065-226524754
PCS90 16128488-16128489-49176329-16129485
PCS91 16128491-16128493-16129632
PCS92 16128507-16128554-67005950-16131231
PCS93 16128519-16128615-90111173-16130852
PCS94 16128520-16128556-90111412-90111174
PCS95 16128547-16128568-90111548-16131772
PCS96 16128552-16128593-162135894-90111547-16130743
PCS97 16128555-16128561-16128815
PCS98 16128560-16128594-49176045-16128813
PCS99 16128564-16128574-16131330-16130736
PCS100 16128573-16128749-16129838

Table S4. The EASE scores of predicted regulon clusters based on CRS, GFR and PCS.

EASE	CRS	CRS-c	GFR	PCS
**1**	4.72E-14	1.11E-12	2.02E-08	0.0202565
**2**	1.09E-13	9.24E-07	9.23E-06	0.00626589
**3**	1.22E-13	0.00235294	0.00548588	0.066413
**4**	2.74E-12	0.000739571	5.72E-06	0.00782775
**5**	1.81E-11	0.000167079	6.61E-13	0.102572
**6**	9.96E-11	0.00619299	5.52E-10	0.0909337
**7**	1.86E-10	4.87E-06	8.62E-06	0.0447632
**8**	1.89E-10	1.85E-06	0.0447632	0.000372416
**9**	8.47E-10	4.33E-07	0.122333	0.00938777
**10**	1.45E-09	2.77E-05	0.00783236	0.00313664
**11**	2.10E-09	1.85E-06	0.0575217	0.0612602
**12**	2.17E-09	2.80E-08	0.0139282	0.032568
**13**	5.35E-09	4.33E-07	0.000579284	0.0218018
**14**	5.98E-08	3.14E-06	0.0218018	0.00313664
**15**	6.37E-08	0.00178822	0.00182292	0.00470311
**16**	7.24E-08	0.000121573	1.25E-05	0.00626589
**17**	9.56E-08	0.0025341	0.0447632	0.00313664

**18**	1.30E-07	0.00548588	0.00195108	0.00313664
**19**	1.48E-07	0.00704774	4.16E-09	0.00313664
**20**	1.63E-07	0.00392003	0.00939513	0.0125023
**21**	3.48E-07	5.15E-06	1.11E-05	0.00626589
**22**	4.82E-07	0.00470311	1.30E-08	0.00313664
**23**	1.85E-06	5.15E-06	0.0086139	0.0337573
**24**	2.36E-06	0.00157466	0.0101761	0.0125023
**25**	3.24E-06	1.04E-05	0.0101761	0.00470219
**26**	4.62E-06	0.0029469	0.0163931	0.00938777
**27**	6.39E-06	0.00313664	4.28E-09	0.00587543
**28**	1.54E-05	4.15E-05	0.0156064	0.0125023
**29**	1.55E-05	1.63E-07	0.00470219	0.025261
**30**	1.66E-05	9.92E-05	0.0431615	0.00313664
**31**	1.69E-05	7.18E-05	0.00437077	0.102572
**32**	2.23E-05	0.000426485	0.0264268	0.0125023
**33**	2.31E-05	1.33E-06	0.00352803	0.00313664
**34**	2.88E-05	1.81E-06	4.60E-05	0.00352803
**35**	3.70E-05	0.000110249	0.257835	0.0156064
**36**	5.16E-05	0.00470311	0.018702	0.12655
**37**	5.85E-05	9.23E-06	0.00938961	0.0156095
**38**	6.08E-05	0.00313664	0.00470219	0.0520525
**39**	6.08E-05	7.18E-05	0.0264268	0.010946
**40**	6.91E-05	0.00977701	0.257835	0.00313664
**41**	9.24E-05	0.00392003	0.00782775	0.00392003
**42**	9.51E-05	0.00470311	0.0752779	0.0202565
**43**	9.92E-05	0.000304857	0.00235294	0.00313664
**44**	0.000111162	0.0125023	0.000125145	0.00977701
**45**	0.0001164	0.0013103	0.102572	0.0909337
**46**	0.000126003	0.00470311	0.0171603	0.000739571
**47**	0.000151061	0.00313664	0.0667462	0.00782775
**48**	0.00026388	5.15E-06	0.00470219	0.00313664
**49**	0.000265133	9.92E-05	0.000171393	0.00626589
**50**	0.000287032	0.00392003	0.0337573	0.0125023
**51**	0.000318714	0.00313664	0.00235294	0.0187093
**52**	0.000339261	0.000904277	0.00782775	0.00470219
**53**	0.000373133	0.00548588	0.00352803	0.00313664
**54**	0.00038315	0.00326861	0.00049159	0.00235294
**55**	0.000414649	5.02E-05	0.0271723	0.00313664
**56**	0.000449229	0.00352803	0.00782775	0.0264268
**57**	0.000474222	0.00626589	0.0198825	0.0909337
**58**	0.000563523	0.00271154	0.00938777	0.0909337

**59**	0.000640507	0.000904277	0.0125023	0.0688073
**60**	0.000663805	0.00470219	0.00235294	0.00782775
**61**	0.000739571	0.00587543	0.00568411	0.00235294
**62**	0.000765221	0.00977701	0.0730683	0.021789
**63**	0.000765221	0.000739571	0.0667462	0.112365
**64**	0.000766307	0.000313137	0.00392003	0.00626589
**65**	0.00077915	6.86E-08	0.0013103	0.00178822
**66**	0.000791962	0.00392003	3.85E-05	0.00392003
**67**	0.000948039	0.00156893	9.23E-06	0.00938777
**68**	0.00104791	0.00235294	0.0014355	0.00470219
**69**	0.00111964	0.00392003	0.0686359	0.010946
**70**	0.00112551	0.00235294	0.00313664	0.0125023
**71**	0.00112551	0.00392003	0.0198825	0.0688073
**72**	0.00130124	0.00313664	0.00782775	5.54E-06
**73**	0.00130344	0.00313664	0.0143776	0.00352803
**74**	0.00138033	0.0447632	5.26E-07	0.00313664
**75**	0.00149821	0.00027759	0.00392003	0.0032776
**76**	0.00155399	0.0140623	1.69E-06	0.00392003
**77**	0.00164528	0.00313664	0.0156095	0.00235294
**78**	0.0024872	0.00470311	0.00704774	0.00626589
**79**	0.003679	9.23E-06	0.0152282	0.0462794
**80**	0.00370819	0.000904277	0.200359	0.00352803
**81**	0.00461326	0.00977701	9.24E-07	0.00178822
**82**	0.00487906	0.00587543	0.00313664	0.00392003
**83**	0.00512806	0.00626589	0.00938961	0.00938777

Table S5. Regulon coverage score analysis of top 100 clusters for CRS, GFR and PCS.

regulon	reg_size	score	top_n	top_size	overlap	RCS	overlap_coe
**Fis**	60	CRS	5	91	8	0.133333	0.133333
**FNR**	95	CRS	5	91	14	0.147368	0.153846
**H-NS**	61	CRS	5	91	3	0.04918	0.04918
**FruR**	33	CRS	5	91	6	0.181818	0.181818
**Lrp**	30	CRS	5	91	1	0.033333	0.033333
**LexA**	29	CRS	5	91	11	0.37931	0.37931
**IHF**	68	CRS	5	91	4	0.058824	0.058824
**NsrR**	32	CRS	5	91	4	0.125	0.125
**CRP**	183	CRS	5	91	19	0.103825	0.208791
**MarA**	21	CRS	5	91	0	0	0
**PhoP**	29	CRS	5	91	1	0.034483	0.034483
**ArcA**	58	CRS	5	91	4	0.068966	0.068966
**NarL**	29	CRS	5	91	4	0.137931	0.137931
**Fur**	45	CRS	5	91	1	0.022222	0.022222

**CpxR**	30	CRS	5	91	2	0.066667	0.066667
**SoxS**	21	CRS	5	91	0	0	0
**Fis**	60	CRS	10	180	11	0.183333	0.183333
**FNR**	95	CRS	10	180	19	0.2	0.2
**H-NS**	61	CRS	10	180	3	0.04918	0.04918
**FruR**	33	CRS	10	180	8	0.242424	0.242424
**Lrp**	30	CRS	10	180	1	0.033333	0.033333
**LexA**	29	CRS	10	180	12	0.413793	0.413793
**IHF**	68	CRS	10	180	9	0.132353	0.132353
**NsrR**	32	CRS	10	180	6	0.1875	0.1875
**CRP**	183	CRS	10	180	23	0.125683	0.127778
**MarA**	21	CRS	10	180	0	0	0
**PhoP**	29	CRS	10	180	2	0.068966	0.068966
**ArcA**	58	CRS	10	180	8	0.137931	0.137931
**NarL**	29	CRS	10	180	6	0.206897	0.206897
**Fur**	45	CRS	10	180	2	0.044444	0.044444
**CpxR**	30	CRS	10	180	2	0.066667	0.066667
**SoxS**	21	CRS	10	180	0	0	0
**Fis**	60	CRS	15	366	16	0.266667	0.266667
**FNR**	95	CRS	15	366	29	0.305263	0.305263
**H-NS**	61	CRS	15	366	13	0.213115	0.213115
**FruR**	33	CRS	15	366	11	0.333333	0.333333
**Lrp**	30	CRS	15	366	2	0.066667	0.066667
**LexA**	29	CRS	15	366	13	0.448276	0.448276
**IHF**	68	CRS	15	366	14	0.205882	0.205882
**NsrR**	32	CRS	15	366	10	0.3125	0.3125
**CRP**	183	CRS	15	366	42	0.229508	0.229508
**MarA**	21	CRS	15	366	1	0.047619	0.047619
**PhoP**	29	CRS	15	366	4	0.137931	0.137931
**ArcA**	58	CRS	15	366	15	0.258621	0.258621
**NarL**	29	CRS	15	366	7	0.241379	0.241379
**Fur**	45	CRS	15	366	19	0.422222	0.422222
**CpxR**	30	CRS	15	366	2	0.066667	0.066667
**SoxS**	21	CRS	15	366	0	0	0
**Fis**	60	CRS	20	667	19	0.316667	0.316667
**FNR**	95	CRS	20	667	42	0.442105	0.442105
**H-NS**	61	CRS	20	667	21	0.344262	0.344262
**FruR**	33	CRS	20	667	15	0.454545	0.454545
**Lrp**	30	CRS	20	667	6	0.2	0.2
**LexA**	29	CRS	20	667	14	0.482759	0.482759
**IHF**	68	CRS	20	667	22	0.323529	0.323529
**NsrR**	32	CRS	20	667	16	0.5	0.5
**CRP**	183	CRS	20	667	70	0.382514	0.382514
**MarA**	21	CRS	20	667	5	0.238095	0.238095
**PhoP**	29	CRS	20	667	11	0.37931	0.37931
**ArcA**	58	CRS	20	667	28	0.482759	0.482759
**NarL**	29	CRS	20	667	11	0.37931	0.37931

**Fur**	45	CRS	20	667	28	0.622222	0.622222
**CpxR**	30	CRS	20	667	6	0.2	0.2
**SoxS**	21	CRS	20	667	3	0.142857	0.142857
**Fis**	60	CRS	25	1110	29	0.483333	0.483333
**FNR**	95	CRS	25	1110	61	0.642105	0.642105
**H-NS**	61	CRS	25	1110	40	0.655738	0.655738
**FruR**	33	CRS	25	1110	24	0.727273	0.727273
**Lrp**	30	CRS	25	1110	18	0.6	0.6
**LexA**	29	CRS	25	1110	16	0.551724	0.551724
**IHF**	68	CRS	25	1110	44	0.647059	0.647059
**NsrR**	32	CRS	25	1110	20	0.625	0.625
**CRP**	183	CRS	25	1110	102	0.557377	0.557377
**MarA**	21	CRS	25	1110	11	0.52381	0.52381
**PhoP**	29	CRS	25	1110	20	0.689655	0.689655
**ArcA**	58	CRS	25	1110	36	0.62069	0.62069
**NarL**	29	CRS	25	1110	14	0.482759	0.482759
**Fur**	45	CRS	25	1110	35	0.777778	0.777778
**CpxR**	30	CRS	25	1110	12	0.4	0.4
**SoxS**	21	CRS	25	1110	7	0.333333	0.333333
**Fis**	60	CRS	30	1206	30	0.5	0.5
**FNR**	95	CRS	30	1206	62	0.652632	0.652632
**H-NS**	61	CRS	30	1206	41	0.672131	0.672131
**FruR**	33	CRS	30	1206	24	0.727273	0.727273
**Lrp**	30	CRS	30	1206	19	0.633333	0.633333
**LexA**	29	CRS	30	1206	16	0.551724	0.551724
**IHF**	68	CRS	30	1206	46	0.676471	0.676471
**NsrR**	32	CRS	30	1206	21	0.65625	0.65625
**CRP**	183	CRS	30	1206	109	0.595628	0.595628
**MarA**	21	CRS	30	1206	12	0.571429	0.571429
**PhoP**	29	CRS	30	1206	23	0.793103	0.793103
**ArcA**	58	CRS	30	1206	38	0.655172	0.655172
**NarL**	29	CRS	30	1206	15	0.517241	0.517241
**Fur**	45	CRS	30	1206	36	0.8	0.8
**CpxR**	30	CRS	30	1206	15	0.5	0.5
**SoxS**	21	CRS	30	1206	7	0.333333	0.333333
**Fis**	60	CRS	35	1353	35	0.583333	0.583333
**FNR**	95	CRS	35	1353	67	0.705263	0.705263
**H-NS**	61	CRS	35	1353	46	0.754098	0.754098
**FruR**	33	CRS	35	1353	25	0.757576	0.757576
**Lrp**	30	CRS	35	1353	20	0.666667	0.666667
**LexA**	29	CRS	35	1353	18	0.62069	0.62069
**IHF**	68	CRS	35	1353	52	0.764706	0.764706
**NsrR**	32	CRS	35	1353	23	0.71875	0.71875
**CRP**	183	CRS	35	1353	122	0.666667	0.666667
**MarA**	21	CRS	35	1353	13	0.619048	0.619048
**PhoP**	29	CRS	35	1353	24	0.827586	0.827586
**ArcA**	58	CRS	35	1353	41	0.706897	0.706897

**NarL**	29	CRS	35	1353	17	0.586207	0.586207
**Fur**	45	CRS	35	1353	38	0.844444	0.844444
**CpxR**	30	CRS	35	1353	15	0.5	0.5
**SoxS**	21	CRS	35	1353	9	0.428571	0.428571
**Fis**	60	CRS	40	1426	36	0.6	0.6
**FNR**	95	CRS	40	1426	69	0.726316	0.726316
**H-NS**	61	CRS	40	1426	49	0.803279	0.803279
**FruR**	33	CRS	40	1426	25	0.757576	0.757576
**Lrp**	30	CRS	40	1426	20	0.666667	0.666667
**LexA**	29	CRS	40	1426	19	0.655172	0.655172
**IHF**	68	CRS	40	1426	52	0.764706	0.764706
**NsrR**	32	CRS	40	1426	23	0.71875	0.71875
**CRP**	183	CRS	40	1426	125	0.68306	0.68306
**MarA**	21	CRS	40	1426	13	0.619048	0.619048
**PhoP**	29	CRS	40	1426	24	0.827586	0.827586
**ArcA**	58	CRS	40	1426	41	0.706897	0.706897
**NarL**	29	CRS	40	1426	17	0.586207	0.586207
**Fur**	45	CRS	40	1426	39	0.866667	0.866667
**CpxR**	30	CRS	40	1426	16	0.533333	0.533333
**SoxS**	21	CRS	40	1426	9	0.428571	0.428571
**Fis**	60	CRS	45	1657	41	0.683333	0.683333
**FNR**	95	CRS	45	1657	72	0.757895	0.757895
**H-NS**	61	CRS	45	1657	54	0.885246	0.885246
**FruR**	33	CRS	45	1657	29	0.878788	0.878788
**Lrp**	30	CRS	45	1657	21	0.7	0.7
**LexA**	29	CRS	45	1657	20	0.689655	0.689655
**IHF**	68	CRS	45	1657	56	0.823529	0.823529
**NsrR**	32	CRS	45	1657	24	0.75	0.75
**CRP**	183	CRS	45	1657	131	0.715847	0.715847
**MarA**	21	CRS	45	1657	14	0.666667	0.666667
**PhoP**	29	CRS	45	1657	26	0.896552	0.896552
**ArcA**	58	CRS	45	1657	46	0.793103	0.793103
**NarL**	29	CRS	45	1657	19	0.655172	0.655172
**Fur**	45	CRS	45	1657	39	0.866667	0.866667
**CpxR**	30	CRS	45	1657	19	0.633333	0.633333
**SoxS**	21	CRS	45	1657	11	0.52381	0.52381
**Fis**	60	CRS	50	1786	43	0.716667	0.716667
**FNR**	95	CRS	50	1786	74	0.778947	0.778947
**H-NS**	61	CRS	50	1786	55	0.901639	0.901639
**FruR**	33	CRS	50	1786	30	0.909091	0.909091
**Lrp**	30	CRS	50	1786	23	0.766667	0.766667
**LexA**	29	CRS	50	1786	21	0.724138	0.724138
**IHF**	68	CRS	50	1786	58	0.852941	0.852941
**NsrR**	32	CRS	50	1786	24	0.75	0.75
**CRP**	183	CRS	50	1786	134	0.73224	0.73224
**MarA**	21	CRS	50	1786	15	0.714286	0.714286
**PhoP**	29	CRS	50	1786	26	0.896552	0.896552

**ArcA**	58	CRS	50	1786	46	0.793103	0.793103
**NarL**	29	CRS	50	1786	19	0.655172	0.655172
**Fur**	45	CRS	50	1786	39	0.866667	0.866667
**CpxR**	30	CRS	50	1786	20	0.666667	0.666667
**SoxS**	21	CRS	50	1786	12	0.571429	0.571429
**Fis**	60	CRS	55	2021	47	0.783333	0.783333
**FNR**	95	CRS	55	2021	81	0.852632	0.852632
**H-NS**	61	CRS	55	2021	58	0.95082	0.95082
**FruR**	33	CRS	55	2021	31	0.939394	0.939394
**Lrp**	30	CRS	55	2021	24	0.8	0.8
**LexA**	29	CRS	55	2021	21	0.724138	0.724138
**IHF**	68	CRS	55	2021	63	0.926471	0.926471
**NsrR**	32	CRS	55	2021	25	0.78125	0.78125
**CRP**	183	CRS	55	2021	145	0.79235	0.79235
**MarA**	21	CRS	55	2021	15	0.714286	0.714286
**PhoP**	29	CRS	55	2021	27	0.931034	0.931034
**ArcA**	58	CRS	55	2021	51	0.87931	0.87931
**NarL**	29	CRS	55	2021	23	0.793103	0.793103
**Fur**	45	CRS	55	2021	40	0.888889	0.888889
**CpxR**	30	CRS	55	2021	22	0.733333	0.733333
**SoxS**	21	CRS	55	2021	13	0.619048	0.619048
**Fis**	60	CRS	60	2157	47	0.783333	0.783333
**FNR**	95	CRS	60	2157	84	0.884211	0.884211
**H-NS**	61	CRS	60	2157	58	0.95082	0.95082
**FruR**	33	CRS	60	2157	32	0.969697	0.969697
**Lrp**	30	CRS	60	2157	26	0.866667	0.866667
**LexA**	29	CRS	60	2157	25	0.862069	0.862069
**IHF**	68	CRS	60	2157	65	0.955882	0.955882
**NsrR**	32	CRS	60	2157	27	0.84375	0.84375
**CRP**	183	CRS	60	2157	150	0.819672	0.819672
**MarA**	21	CRS	60	2157	16	0.761905	0.761905
**PhoP**	29	CRS	60	2157	27	0.931034	0.931034
**ArcA**	58	CRS	60	2157	53	0.913793	0.913793
**NarL**	29	CRS	60	2157	24	0.827586	0.827586
**Fur**	45	CRS	60	2157	40	0.888889	0.888889
**CpxR**	30	CRS	60	2157	23	0.766667	0.766667
**SoxS**	21	CRS	60	2157	15	0.714286	0.714286
**Fis**	60	CRS	65	2239	49	0.816667	0.816667
**FNR**	95	CRS	65	2239	86	0.905263	0.905263
**H-NS**	61	CRS	65	2239	58	0.95082	0.95082
**FruR**	33	CRS	65	2239	32	0.969697	0.969697
**Lrp**	30	CRS	65	2239	26	0.866667	0.866667
**LexA**	29	CRS	65	2239	25	0.862069	0.862069
**IHF**	68	CRS	65	2239	65	0.955882	0.955882
**NsrR**	32	CRS	65	2239	27	0.84375	0.84375
**CRP**	183	CRS	65	2239	157	0.857923	0.857923
**MarA**	21	CRS	65	2239	17	0.809524	0.809524

**PhoP**	29	CRS	65	2239	27	0.931034	0.931034
**ArcA**	58	CRS	65	2239	54	0.931034	0.931034
**NarL**	29	CRS	65	2239	24	0.827586	0.827586
**Fur**	45	CRS	65	2239	41	0.911111	0.911111
**CpxR**	30	CRS	65	2239	24	0.8	0.8
**SoxS**	21	CRS	65	2239	16	0.761905	0.761905
**Fis**	60	CRS	70	2360	50	0.833333	0.833333
**FNR**	95	CRS	70	2360	87	0.915789	0.915789
**H-NS**	61	CRS	70	2360	59	0.967213	0.967213
**FruR**	33	CRS	70	2360	33	1	1
**Lrp**	30	CRS	70	2360	26	0.866667	0.866667
**LexA**	29	CRS	70	2360	26	0.896552	0.896552
**IHF**	68	CRS	70	2360	67	0.985294	0.985294
**NsrR**	32	CRS	70	2360	28	0.875	0.875
**CRP**	183	CRS	70	2360	163	0.89071	0.89071
**MarA**	21	CRS	70	2360	17	0.809524	0.809524
**PhoP**	29	CRS	70	2360	27	0.931034	0.931034
**ArcA**	58	CRS	70	2360	54	0.931034	0.931034
**NarL**	29	CRS	70	2360	24	0.827586	0.827586
**Fur**	45	CRS	70	2360	43	0.955556	0.955556
**CpxR**	30	CRS	70	2360	24	0.8	0.8
**SoxS**	21	CRS	70	2360	16	0.761905	0.761905
**Fis**	60	CRS	75	2430	51	0.85	0.85
**FNR**	95	CRS	75	2430	87	0.915789	0.915789
**H-NS**	61	CRS	75	2430	59	0.967213	0.967213
**FruR**	33	CRS	75	2430	33	1	1
**Lrp**	30	CRS	75	2430	27	0.9	0.9
**LexA**	29	CRS	75	2430	27	0.931034	0.931034
**IHF**	68	CRS	75	2430	67	0.985294	0.985294
**NsrR**	32	CRS	75	2430	29	0.90625	0.90625
**CRP**	183	CRS	75	2430	165	0.901639	0.901639
**MarA**	21	CRS	75	2430	18	0.857143	0.857143
**PhoP**	29	CRS	75	2430	27	0.931034	0.931034
**ArcA**	58	CRS	75	2430	54	0.931034	0.931034
**NarL**	29	CRS	75	2430	24	0.827586	0.827586
**Fur**	45	CRS	75	2430	43	0.955556	0.955556
**CpxR**	30	CRS	75	2430	24	0.8	0.8
**SoxS**	21	CRS	75	2430	17	0.809524	0.809524
**Fis**	60	CRS	80	2467	52	0.866667	0.866667
**FNR**	95	CRS	80	2467	87	0.915789	0.915789
**H-NS**	61	CRS	80	2467	59	0.967213	0.967213
**FruR**	33	CRS	80	2467	33	1	1
**Lrp**	30	CRS	80	2467	28	0.933333	0.933333
**LexA**	29	CRS	80	2467	28	0.965517	0.965517
**IHF**	68	CRS	80	2467	67	0.985294	0.985294
**NsrR**	32	CRS	80	2467	29	0.90625	0.90625
**CRP**	183	CRS	80	2467	166	0.907104	0.907104

**MarA**	21	CRS	80	2467	18	0.857143	0.857143
**PhoP**	29	CRS	80	2467	28	0.965517	0.965517
**ArcA**	58	CRS	80	2467	54	0.931034	0.931034
**NarL**	29	CRS	80	2467	24	0.827586	0.827586
**Fur**	45	CRS	80	2467	43	0.955556	0.955556
**CpxR**	30	CRS	80	2467	24	0.8	0.8
**SoxS**	21	CRS	80	2467	18	0.857143	0.857143
**Fis**	60	CRS	85	2483	52	0.866667	0.866667
**FNR**	95	CRS	85	2483	88	0.926316	0.926316
**H-NS**	61	CRS	85	2483	59	0.967213	0.967213
**FruR**	33	CRS	85	2483	33	1	1
**Lrp**	30	CRS	85	2483	28	0.933333	0.933333
**LexA**	29	CRS	85	2483	28	0.965517	0.965517
**IHF**	68	CRS	85	2483	68	1	1
**NsrR**	32	CRS	85	2483	29	0.90625	0.90625
**CRP**	183	CRS	85	2483	166	0.907104	0.907104
**MarA**	21	CRS	85	2483	18	0.857143	0.857143
**PhoP**	29	CRS	85	2483	28	0.965517	0.965517
**ArcA**	58	CRS	85	2483	55	0.948276	0.948276
**NarL**	29	CRS	85	2483	25	0.862069	0.862069
**Fur**	45	CRS	85	2483	43	0.955556	0.955556
**CpxR**	30	CRS	85	2483	24	0.8	0.8
**SoxS**	21	CRS	85	2483	18	0.857143	0.857143
**Fis**	60	CRS	90	2565	55	0.916667	0.916667
**FNR**	95	CRS	90	2565	90	0.947368	0.947368
**H-NS**	61	CRS	90	2565	59	0.967213	0.967213
**FruR**	33	CRS	90	2565	33	1	1
**Lrp**	30	CRS	90	2565	29	0.966667	0.966667
**LexA**	29	CRS	90	2565	28	0.965517	0.965517
**IHF**	68	CRS	90	2565	68	1	1
**NsrR**	32	CRS	90	2565	30	0.9375	0.9375
**CRP**	183	CRS	90	2565	174	0.95082	0.95082
**MarA**	21	CRS	90	2565	18	0.857143	0.857143
**PhoP**	29	CRS	90	2565	28	0.965517	0.965517
**ArcA**	58	CRS	90	2565	55	0.948276	0.948276
**NarL**	29	CRS	90	2565	27	0.931034	0.931034
**Fur**	45	CRS	90	2565	43	0.955556	0.955556
**CpxR**	30	CRS	90	2565	25	0.833333	0.833333
**SoxS**	21	CRS	90	2565	18	0.857143	0.857143
**Fis**	60	CRS	95	2614	56	0.933333	0.933333
**FNR**	95	CRS	95	2614	91	0.957895	0.957895
**H-NS**	61	CRS	95	2614	59	0.967213	0.967213
**FruR**	33	CRS	95	2614	33	1	1
**Lrp**	30	CRS	95	2614	29	0.966667	0.966667
**LexA**	29	CRS	95	2614	28	0.965517	0.965517
**IHF**	68	CRS	95	2614	68	1	1
**NsrR**	32	CRS	95	2614	30	0.9375	0.9375

**CRP**	183	CRS	95	2614	175	0.956284	0.956284
**MarA**	21	CRS	95	2614	19	0.904762	0.904762
**PhoP**	29	CRS	95	2614	28	0.965517	0.965517
**ArcA**	58	CRS	95	2614	55	0.948276	0.948276
**NarL**	29	CRS	95	2614	27	0.931034	0.931034
**Fur**	45	CRS	95	2614	43	0.955556	0.955556
**CpxR**	30	CRS	95	2614	27	0.9	0.9
**SoxS**	21	CRS	95	2614	19	0.904762	0.904762
**Fis**	60	CRS	100	2634	56	0.933333	0.933333
**FNR**	95	CRS	100	2634	91	0.957895	0.957895
**H-NS**	61	CRS	100	2634	59	0.967213	0.967213
**FruR**	33	CRS	100	2634	33	1	1
**Lrp**	30	CRS	100	2634	29	0.966667	0.966667
**LexA**	29	CRS	100	2634	28	0.965517	0.965517
**IHF**	68	CRS	100	2634	68	1	1
**NsrR**	32	CRS	100	2634	30	0.9375	0.9375
**CRP**	183	CRS	100	2634	175	0.956284	0.956284
**MarA**	21	CRS	100	2634	19	0.904762	0.904762
**PhoP**	29	CRS	100	2634	28	0.965517	0.965517
**ArcA**	58	CRS	100	2634	55	0.948276	0.948276
**NarL**	29	CRS	100	2634	27	0.931034	0.931034
**Fur**	45	CRS	100	2634	43	0.955556	0.955556
**CpxR**	30	CRS	100	2634	27	0.9	0.9
**SoxS**	21	CRS	100	2634	19	0.904762	0.904762
**Fis**	60	GFR	5	32	1	0.016667	0.03125
**FNR**	95	GFR	5	32	4	0.042105	0.125
**H-NS**	61	GFR	5	32	0	0	0
**FruR**	33	GFR	5	32	0	0	0
**Lrp**	30	GFR	5	32	1	0.033333	0.033333
**LexA**	29	GFR	5	32	0	0	0
**IHF**	68	GFR	5	32	1	0.014706	0.03125
**NsrR**	32	GFR	5	32	0	0	0
**CRP**	183	GFR	5	32	2	0.010929	0.0625
**MarA**	21	GFR	5	32	0	0	0
**PhoP**	29	GFR	5	32	0	0	0
**ArcA**	58	GFR	5	32	0	0	0
**NarL**	29	GFR	5	32	0	0	0
**Fur**	45	GFR	5	32	1	0.022222	0.03125
**CpxR**	30	GFR	5	32	0	0	0
**SoxS**	21	GFR	5	32	0	0	0
**Fis**	60	GFR	10	81	5	0.083333	0.083333
**FNR**	95	GFR	10	81	6	0.063158	0.074074
**H-NS**	61	GFR	10	81	0	0	0
**FruR**	33	GFR	10	81	7	0.212121	0.212121
**Lrp**	30	GFR	10	81	3	0.1	0.1
**LexA**	29	GFR	10	81	0	0	0
**IHF**	68	GFR	10	81	3	0.044118	0.044118

**NsrR**	32	GFR	10	81	0	0	0
**CRP**	183	GFR	10	81	8	0.043716	0.098765
**MarA**	21	GFR	10	81	0	0	0
**PhoP**	29	GFR	10	81	1	0.034483	0.034483
**ArcA**	58	GFR	10	81	1	0.017241	0.017241
**NarL**	29	GFR	10	81	1	0.034483	0.034483
**Fur**	45	GFR	10	81	2	0.044444	0.044444
**CpxR**	30	GFR	10	81	0	0	0
**SoxS**	21	GFR	10	81	2	0.095238	0.095238
**Fis**	60	GFR	15	117	8	0.133333	0.133333
**FNR**	95	GFR	15	117	7	0.073684	0.073684
**H-NS**	61	GFR	15	117	0	0	0
**FruR**	33	GFR	15	117	7	0.212121	0.212121
**Lrp**	30	GFR	15	117	3	0.1	0.1
**LexA**	29	GFR	15	117	0	0	0
**IHF**	68	GFR	15	117	3	0.044118	0.044118
**NsrR**	32	GFR	15	117	0	0	0
**CRP**	183	GFR	15	117	10	0.054645	0.08547
**MarA**	21	GFR	15	117	1	0.047619	0.047619
**PhoP**	29	GFR	15	117	1	0.034483	0.034483
**ArcA**	58	GFR	15	117	1	0.017241	0.017241
**NarL**	29	GFR	15	117	1	0.034483	0.034483
**Fur**	45	GFR	15	117	2	0.044444	0.044444
**CpxR**	30	GFR	15	117	1	0.033333	0.033333
**SoxS**	21	GFR	15	117	2	0.095238	0.095238
**Fis**	60	GFR	20	161	9	0.15	0.15
**FNR**	95	GFR	20	161	9	0.094737	0.094737
**H-NS**	61	GFR	20	161	1	0.016393	0.016393
**FruR**	33	GFR	20	161	7	0.212121	0.212121
**Lrp**	30	GFR	20	161	5	0.166667	0.166667
**LexA**	29	GFR	20	161	7	0.241379	0.241379
**IHF**	68	GFR	20	161	3	0.044118	0.044118
**NsrR**	32	GFR	20	161	1	0.03125	0.03125
**CRP**	183	GFR	20	161	11	0.060109	0.068323
**MarA**	21	GFR	20	161	1	0.047619	0.047619
**PhoP**	29	GFR	20	161	1	0.034483	0.034483
**ArcA**	58	GFR	20	161	4	0.068966	0.068966
**NarL**	29	GFR	20	161	1	0.034483	0.034483
**Fur**	45	GFR	20	161	2	0.044444	0.044444
**CpxR**	30	GFR	20	161	2	0.066667	0.066667
**SoxS**	21	GFR	20	161	2	0.095238	0.095238
**Fis**	60	GFR	25	206	12	0.2	0.2
**FNR**	95	GFR	25	206	9	0.094737	0.094737
**H-NS**	61	GFR	25	206	3	0.04918	0.04918
**FruR**	33	GFR	25	206	7	0.212121	0.212121
**Lrp**	30	GFR	25	206	6	0.2	0.2
**LexA**	29	GFR	25	206	7	0.241379	0.241379

**IHF**	68	GFR	25	206	4	0.058824	0.058824
**NsrR**	32	GFR	25	206	1	0.03125	0.03125
**CRP**	183	GFR	25	206	19	0.103825	0.103825
**MarA**	21	GFR	25	206	1	0.047619	0.047619
**PhoP**	29	GFR	25	206	2	0.068966	0.068966
**ArcA**	58	GFR	25	206	5	0.086207	0.086207
**NarL**	29	GFR	25	206	1	0.034483	0.034483
**Fur**	45	GFR	25	206	4	0.088889	0.088889
**CpxR**	30	GFR	25	206	3	0.1	0.1
**SoxS**	21	GFR	25	206	2	0.095238	0.095238
**Fis**	60	GFR	30	252	12	0.2	0.2
**FNR**	95	GFR	30	252	12	0.126316	0.126316
**H-NS**	61	GFR	30	252	6	0.098361	0.098361
**FruR**	33	GFR	30	252	9	0.272727	0.272727
**Lrp**	30	GFR	30	252	7	0.233333	0.233333
**LexA**	29	GFR	30	252	7	0.241379	0.241379
**IHF**	68	GFR	30	252	4	0.058824	0.058824
**NsrR**	32	GFR	30	252	1	0.03125	0.03125
**CRP**	183	GFR	30	252	23	0.125683	0.125683
**MarA**	21	GFR	30	252	2	0.095238	0.095238
**PhoP**	29	GFR	30	252	4	0.137931	0.137931
**ArcA**	58	GFR	30	252	7	0.12069	0.12069
**NarL**	29	GFR	30	252	2	0.068966	0.068966
**Fur**	45	GFR	30	252	5	0.111111	0.111111
**CpxR**	30	GFR	30	252	3	0.1	0.1
**SoxS**	21	GFR	30	252	3	0.142857	0.142857
**Fis**	60	GFR	35	282	12	0.2	0.2
**FNR**	95	GFR	35	282	15	0.157895	0.157895
**H-NS**	61	GFR	35	282	9	0.147541	0.147541
**FruR**	33	GFR	35	282	10	0.30303	0.30303
**Lrp**	30	GFR	35	282	7	0.233333	0.233333
**LexA**	29	GFR	35	282	7	0.241379	0.241379
**IHF**	68	GFR	35	282	6	0.088235	0.088235
**NsrR**	32	GFR	35	282	1	0.03125	0.03125
**CRP**	183	GFR	35	282	24	0.131148	0.131148
**MarA**	21	GFR	35	282	3	0.142857	0.142857
**PhoP**	29	GFR	35	282	4	0.137931	0.137931
**ArcA**	58	GFR	35	282	8	0.137931	0.137931
**NarL**	29	GFR	35	282	3	0.103448	0.103448
**Fur**	45	GFR	35	282	11	0.244444	0.244444
**CpxR**	30	GFR	35	282	3	0.1	0.1
**SoxS**	21	GFR	35	282	4	0.190476	0.190476
**Fis**	60	GFR	40	304	14	0.233333	0.233333
**FNR**	95	GFR	40	304	16	0.168421	0.168421
**H-NS**	61	GFR	40	304	10	0.163934	0.163934
**FruR**	33	GFR	40	304	10	0.30303	0.30303
**Lrp**	30	GFR	40	304	8	0.266667	0.266667

**LexA**	29	GFR	40	304	7	0.241379	0.241379
**IHF**	68	GFR	40	304	7	0.102941	0.102941
**NsrR**	32	GFR	40	304	1	0.03125	0.03125
**CRP**	183	GFR	40	304	27	0.147541	0.147541
**MarA**	21	GFR	40	304	3	0.142857	0.142857
**PhoP**	29	GFR	40	304	4	0.137931	0.137931
**ArcA**	58	GFR	40	304	9	0.155172	0.155172
**NarL**	29	GFR	40	304	3	0.103448	0.103448
**Fur**	45	GFR	40	304	13	0.288889	0.288889
**CpxR**	30	GFR	40	304	4	0.133333	0.133333
**SoxS**	21	GFR	40	304	4	0.190476	0.190476
**Fis**	60	GFR	45	329	14	0.233333	0.233333
**FNR**	95	GFR	45	329	17	0.178947	0.178947
**H-NS**	61	GFR	45	329	11	0.180328	0.180328
**FruR**	33	GFR	45	329	11	0.333333	0.333333
**Lrp**	30	GFR	45	329	8	0.266667	0.266667
**LexA**	29	GFR	45	329	7	0.241379	0.241379
**IHF**	68	GFR	45	329	8	0.117647	0.117647
**NsrR**	32	GFR	45	329	1	0.03125	0.03125
**CRP**	183	GFR	45	329	29	0.15847	0.15847
**MarA**	21	GFR	45	329	3	0.142857	0.142857
**PhoP**	29	GFR	45	329	4	0.137931	0.137931
**ArcA**	58	GFR	45	329	10	0.172414	0.172414
**NarL**	29	GFR	45	329	3	0.103448	0.103448
**Fur**	45	GFR	45	329	13	0.288889	0.288889
**CpxR**	30	GFR	45	329	4	0.133333	0.133333
**SoxS**	21	GFR	45	329	4	0.190476	0.190476
**Fis**	60	GFR	50	350	15	0.25	0.25
**FNR**	95	GFR	50	350	17	0.178947	0.178947
**H-NS**	61	GFR	50	350	11	0.180328	0.180328
**FruR**	33	GFR	50	350	11	0.333333	0.333333
**Lrp**	30	GFR	50	350	9	0.3	0.3
**LexA**	29	GFR	50	350	7	0.241379	0.241379
**IHF**	68	GFR	50	350	8	0.117647	0.117647
**NsrR**	32	GFR	50	350	1	0.03125	0.03125
**CRP**	183	GFR	50	350	31	0.169399	0.169399
**MarA**	21	GFR	50	350	3	0.142857	0.142857
**PhoP**	29	GFR	50	350	5	0.172414	0.172414
**ArcA**	58	GFR	50	350	10	0.172414	0.172414
**NarL**	29	GFR	50	350	3	0.103448	0.103448
**Fur**	45	GFR	50	350	13	0.288889	0.288889
**CpxR**	30	GFR	50	350	4	0.133333	0.133333
**SoxS**	21	GFR	50	350	4	0.190476	0.190476
**Fis**	60	GFR	55	386	15	0.25	0.25
**FNR**	95	GFR	55	386	19	0.2	0.2
**H-NS**	61	GFR	55	386	12	0.196721	0.196721
**FruR**	33	GFR	55	386	12	0.363636	0.363636

**Lrp**	30	GFR	55	386	9	0.3	0.3
**LexA**	29	GFR	55	386	7	0.241379	0.241379
**IHF**	68	GFR	55	386	11	0.161765	0.161765
**NsrR**	32	GFR	55	386	1	0.03125	0.03125
**CRP**	183	GFR	55	386	37	0.202186	0.202186
**MarA**	21	GFR	55	386	3	0.142857	0.142857
**PhoP**	29	GFR	55	386	6	0.206897	0.206897
**ArcA**	58	GFR	55	386	11	0.189655	0.189655
**NarL**	29	GFR	55	386	4	0.137931	0.137931
**Fur**	45	GFR	55	386	18	0.4	0.4
**CpxR**	30	GFR	55	386	4	0.133333	0.133333
**SoxS**	21	GFR	55	386	4	0.190476	0.190476
**Fis**	60	GFR	60	407	15	0.25	0.25
**FNR**	95	GFR	60	407	20	0.210526	0.210526
**H-NS**	61	GFR	60	407	12	0.196721	0.196721
**FruR**	33	GFR	60	407	12	0.363636	0.363636
**Lrp**	30	GFR	60	407	9	0.3	0.3
**LexA**	29	GFR	60	407	8	0.275862	0.275862
**IHF**	68	GFR	60	407	12	0.176471	0.176471
**NsrR**	32	GFR	60	407	1	0.03125	0.03125
**CRP**	183	GFR	60	407	39	0.213115	0.213115
**MarA**	21	GFR	60	407	4	0.190476	0.190476
**PhoP**	29	GFR	60	407	6	0.206897	0.206897
**ArcA**	58	GFR	60	407	12	0.206897	0.206897
**NarL**	29	GFR	60	407	4	0.137931	0.137931
**Fur**	45	GFR	60	407	21	0.466667	0.466667
**CpxR**	30	GFR	60	407	4	0.133333	0.133333
**SoxS**	21	GFR	60	407	4	0.190476	0.190476
**Fis**	60	GFR	65	429	17	0.283333	0.283333
**FNR**	95	GFR	65	429	21	0.221053	0.221053
**H-NS**	61	GFR	65	429	12	0.196721	0.196721
**FruR**	33	GFR	65	429	12	0.363636	0.363636
**Lrp**	30	GFR	65	429	9	0.3	0.3
**LexA**	29	GFR	65	429	8	0.275862	0.275862
**IHF**	68	GFR	65	429	12	0.176471	0.176471
**NsrR**	32	GFR	65	429	1	0.03125	0.03125
**CRP**	183	GFR	65	429	42	0.229508	0.229508
**MarA**	21	GFR	65	429	4	0.190476	0.190476
**PhoP**	29	GFR	65	429	8	0.275862	0.275862
**ArcA**	58	GFR	65	429	13	0.224138	0.224138
**NarL**	29	GFR	65	429	4	0.137931	0.137931
**Fur**	45	GFR	65	429	22	0.488889	0.488889
**CpxR**	30	GFR	65	429	4	0.133333	0.133333
**SoxS**	21	GFR	65	429	4	0.190476	0.190476
**Fis**	60	GFR	70	452	19	0.316667	0.316667
**FNR**	95	GFR	70	452	23	0.242105	0.242105
**H-NS**	61	GFR	70	452	13	0.213115	0.213115

**FruR**	33	GFR	70	452	12	0.363636	0.363636
**Lrp**	30	GFR	70	452	9	0.3	0.3
**LexA**	29	GFR	70	452	8	0.275862	0.275862
**IHF**	68	GFR	70	452	13	0.191176	0.191176
**NsrR**	32	GFR	70	452	1	0.03125	0.03125
**CRP**	183	GFR	70	452	44	0.240437	0.240437
**MarA**	21	GFR	70	452	4	0.190476	0.190476
**PhoP**	29	GFR	70	452	9	0.310345	0.310345
**ArcA**	58	GFR	70	452	14	0.241379	0.241379
**NarL**	29	GFR	70	452	5	0.172414	0.172414
**Fur**	45	GFR	70	452	22	0.488889	0.488889
**CpxR**	30	GFR	70	452	4	0.133333	0.133333
**SoxS**	21	GFR	70	452	4	0.190476	0.190476
**Fis**	60	GFR	75	482	20	0.333333	0.333333
**FNR**	95	GFR	75	482	24	0.252632	0.252632
**H-NS**	61	GFR	75	482	14	0.229508	0.229508
**FruR**	33	GFR	75	482	12	0.363636	0.363636
**Lrp**	30	GFR	75	482	10	0.333333	0.333333
**LexA**	29	GFR	75	482	8	0.275862	0.275862
**IHF**	68	GFR	75	482	14	0.205882	0.205882
**NsrR**	32	GFR	75	482	3	0.09375	0.09375
**CRP**	183	GFR	75	482	46	0.251366	0.251366
**MarA**	21	GFR	75	482	4	0.190476	0.190476
**PhoP**	29	GFR	75	482	9	0.310345	0.310345
**ArcA**	58	GFR	75	482	15	0.258621	0.258621
**NarL**	29	GFR	75	482	5	0.172414	0.172414
**Fur**	45	GFR	75	482	22	0.488889	0.488889
**CpxR**	30	GFR	75	482	6	0.2	0.2
**SoxS**	21	GFR	75	482	4	0.190476	0.190476
**Fis**	60	GFR	80	505	20	0.333333	0.333333
**FNR**	95	GFR	80	505	26	0.273684	0.273684
**H-NS**	61	GFR	80	505	15	0.245902	0.245902
**FruR**	33	GFR	80	505	15	0.454545	0.454545
**Lrp**	30	GFR	80	505	10	0.333333	0.333333
**LexA**	29	GFR	80	505	8	0.275862	0.275862
**IHF**	68	GFR	80	505	14	0.205882	0.205882
**NsrR**	32	GFR	80	505	3	0.09375	0.09375
**CRP**	183	GFR	80	505	52	0.284153	0.284153
**MarA**	21	GFR	80	505	5	0.238095	0.238095
**PhoP**	29	GFR	80	505	9	0.310345	0.310345
**ArcA**	58	GFR	80	505	18	0.310345	0.310345
**NarL**	29	GFR	80	505	5	0.172414	0.172414
**Fur**	45	GFR	80	505	23	0.511111	0.511111
**CpxR**	30	GFR	80	505	8	0.266667	0.266667
**SoxS**	21	GFR	80	505	5	0.238095	0.238095
**Fis**	60	GFR	85	530	22	0.366667	0.366667
**FNR**	95	GFR	85	530	28	0.294737	0.294737

**H-NS**	61	GFR	85	530	15	0.245902	0.245902
**FruR**	33	GFR	85	530	16	0.484848	0.484848
**Lrp**	30	GFR	85	530	10	0.333333	0.333333
**LexA**	29	GFR	85	530	8	0.275862	0.275862
**IHF**	68	GFR	85	530	14	0.205882	0.205882
**NsrR**	32	GFR	85	530	4	0.125	0.125
**CRP**	183	GFR	85	530	53	0.289617	0.289617
**MarA**	21	GFR	85	530	6	0.285714	0.285714
**PhoP**	29	GFR	85	530	9	0.310345	0.310345
**ArcA**	58	GFR	85	530	18	0.310345	0.310345
**NarL**	29	GFR	85	530	5	0.172414	0.172414
**Fur**	45	GFR	85	530	24	0.533333	0.533333
**CpxR**	30	GFR	85	530	10	0.333333	0.333333
**SoxS**	21	GFR	85	530	6	0.285714	0.285714
**Fis**	60	GFR	90	553	22	0.366667	0.366667
**FNR**	95	GFR	90	553	28	0.294737	0.294737
**H-NS**	61	GFR	90	553	17	0.278689	0.278689
**FruR**	33	GFR	90	553	18	0.545455	0.545455
**Lrp**	30	GFR	90	553	10	0.333333	0.333333
**LexA**	29	GFR	90	553	8	0.275862	0.275862
**IHF**	68	GFR	90	553	14	0.205882	0.205882
**NsrR**	32	GFR	90	553	4	0.125	0.125
**CRP**	183	GFR	90	553	57	0.311475	0.311475
**MarA**	21	GFR	90	553	6	0.285714	0.285714
**PhoP**	29	GFR	90	553	9	0.310345	0.310345
**ArcA**	58	GFR	90	553	19	0.327586	0.327586
**NarL**	29	GFR	90	553	5	0.172414	0.172414
**Fur**	45	GFR	90	553	24	0.533333	0.533333
**CpxR**	30	GFR	90	553	10	0.333333	0.333333
**SoxS**	21	GFR	90	553	6	0.285714	0.285714
**Fis**	60	GFR	95	574	23	0.383333	0.383333
**FNR**	95	GFR	95	574	28	0.294737	0.294737
**H-NS**	61	GFR	95	574	17	0.278689	0.278689
**FruR**	33	GFR	95	574	19	0.575758	0.575758
**Lrp**	30	GFR	95	574	10	0.333333	0.333333
**LexA**	29	GFR	95	574	8	0.275862	0.275862
**IHF**	68	GFR	95	574	16	0.235294	0.235294
**NsrR**	32	GFR	95	574	4	0.125	0.125
**CRP**	183	GFR	95	574	60	0.327869	0.327869
**MarA**	21	GFR	95	574	6	0.285714	0.285714
**PhoP**	29	GFR	95	574	9	0.310345	0.310345
**ArcA**	58	GFR	95	574	20	0.344828	0.344828
**NarL**	29	GFR	95	574	5	0.172414	0.172414
**Fur**	45	GFR	95	574	24	0.533333	0.533333
**CpxR**	30	GFR	95	574	10	0.333333	0.333333
**SoxS**	21	GFR	95	574	6	0.285714	0.285714
**Fis**	60	GFR	100	590	24	0.4	0.4

**FNR**	95	GFR	100	590	30	0.315789	0.315789
**H-NS**	61	GFR	100	590	17	0.278689	0.278689
**FruR**	33	GFR	100	590	19	0.575758	0.575758
**Lrp**	30	GFR	100	590	10	0.333333	0.333333
**LexA**	29	GFR	100	590	8	0.275862	0.275862
**IHF**	68	GFR	100	590	17	0.25	0.25
**NsrR**	32	GFR	100	590	5	0.15625	0.15625
**CRP**	183	GFR	100	590	61	0.333333	0.333333
**MarA**	21	GFR	100	590	7	0.333333	0.333333
**PhoP**	29	GFR	100	590	10	0.344828	0.344828
**ArcA**	58	GFR	100	590	22	0.37931	0.37931
**NarL**	29	GFR	100	590	5	0.172414	0.172414
**Fur**	45	GFR	100	590	24	0.533333	0.533333
**CpxR**	30	GFR	100	590	10	0.333333	0.333333
**SoxS**	21	GFR	100	590	7	0.333333	0.333333
**Fis**	60	PCS	5	22	1	0.016667	0.045455
**FNR**	95	PCS	5	22	1	0.010526	0.045455
**H-NS**	61	PCS	5	22	0	0	0
**FruR**	33	PCS	5	22	0	0	0
**Lrp**	30	PCS	5	22	0	0	0
**LexA**	29	PCS	5	22	1	0.034483	0.045455
**IHF**	68	PCS	5	22	1	0.014706	0.045455
**NsrR**	32	PCS	5	22	1	0.03125	0.045455
**CRP**	183	PCS	5	22	0	0	0
**MarA**	21	PCS	5	22	0	0	0
**PhoP**	29	PCS	5	22	0	0	0
**ArcA**	58	PCS	5	22	0	0	0
**NarL**	29	PCS	5	22	0	0	0
**Fur**	45	PCS	5	22	0	0	0
**CpxR**	30	PCS	5	22	1	0.033333	0.045455
**SoxS**	21	PCS	5	22	0	0	0
**Fis**	60	PCS	10	42	2	0.033333	0.047619
**FNR**	95	PCS	10	42	1	0.010526	0.02381
**H-NS**	61	PCS	10	42	0	0	0
**FruR**	33	PCS	10	42	0	0	0
**Lrp**	30	PCS	10	42	0	0	0
**LexA**	29	PCS	10	42	2	0.068966	0.068966
**IHF**	68	PCS	10	42	2	0.029412	0.047619
**NsrR**	32	PCS	10	42	1	0.03125	0.03125
**CRP**	183	PCS	10	42	2	0.010929	0.047619
**MarA**	21	PCS	10	42	0	0	0
**PhoP**	29	PCS	10	42	0	0	0
**ArcA**	58	PCS	10	42	0	0	0
**NarL**	29	PCS	10	42	0	0	0
**Fur**	45	PCS	10	42	0	0	0
**CpxR**	30	PCS	10	42	1	0.033333	0.033333
**SoxS**	21	PCS	10	42	0	0	0

**Fis**	60	PCS	15	62	2	0.033333	0.033333
**FNR**	95	PCS	15	62	1	0.010526	0.016129
**H-NS**	61	PCS	15	62	1	0.016393	0.016393
**FruR**	33	PCS	15	62	0	0	0
**Lrp**	30	PCS	15	62	0	0	0
**LexA**	29	PCS	15	62	2	0.068966	0.068966
**IHF**	68	PCS	15	62	3	0.044118	0.048387
**NsrR**	32	PCS	15	62	1	0.03125	0.03125
**CRP**	183	PCS	15	62	4	0.021858	0.064516
**MarA**	21	PCS	15	62	0	0	0
**PhoP**	29	PCS	15	62	2	0.068966	0.068966
**ArcA**	58	PCS	15	62	1	0.017241	0.017241
**NarL**	29	PCS	15	62	0	0	0
**Fur**	45	PCS	15	62	1	0.022222	0.022222
**CpxR**	30	PCS	15	62	1	0.033333	0.033333
**SoxS**	21	PCS	15	62	0	0	0
**Fis**	60	PCS	20	82	2	0.033333	0.033333
**FNR**	95	PCS	20	82	1	0.010526	0.012195
**H-NS**	61	PCS	20	82	1	0.016393	0.016393
**FruR**	33	PCS	20	82	0	0	0
**Lrp**	30	PCS	20	82	0	0	0
**LexA**	29	PCS	20	82	2	0.068966	0.068966
**IHF**	68	PCS	20	82	3	0.044118	0.044118
**NsrR**	32	PCS	20	82	2	0.0625	0.0625
**CRP**	183	PCS	20	82	4	0.021858	0.04878
**MarA**	21	PCS	20	82	1	0.047619	0.047619
**PhoP**	29	PCS	20	82	2	0.068966	0.068966
**ArcA**	58	PCS	20	82	1	0.017241	0.017241
**NarL**	29	PCS	20	82	0	0	0
**Fur**	45	PCS	20	82	1	0.022222	0.022222
**CpxR**	30	PCS	20	82	1	0.033333	0.033333
**SoxS**	21	PCS	20	82	0	0	0
**Fis**	60	PCS	25	104	3	0.05	0.05
**FNR**	95	PCS	25	104	1	0.010526	0.010526
**H-NS**	61	PCS	25	104	1	0.016393	0.016393
**FruR**	33	PCS	25	104	0	0	0
**Lrp**	30	PCS	25	104	0	0	0
**LexA**	29	PCS	25	104	2	0.068966	0.068966
**IHF**	68	PCS	25	104	4	0.058824	0.058824
**NsrR**	32	PCS	25	104	2	0.0625	0.0625
**CRP**	183	PCS	25	104	4	0.021858	0.038462
**MarA**	21	PCS	25	104	1	0.047619	0.047619
**PhoP**	29	PCS	25	104	3	0.103448	0.103448
**ArcA**	58	PCS	25	104	1	0.017241	0.017241
**NarL**	29	PCS	25	104	0	0	0
**Fur**	45	PCS	25	104	1	0.022222	0.022222
**CpxR**	30	PCS	25	104	1	0.033333	0.033333

**SoxS**	21	PCS	25	104	0	0	0
**Fis**	60	PCS	30	123	4	0.066667	0.066667
**FNR**	95	PCS	30	123	3	0.031579	0.031579
**H-NS**	61	PCS	30	123	2	0.032787	0.032787
**FruR**	33	PCS	30	123	1	0.030303	0.030303
**Lrp**	30	PCS	30	123	0	0	0
**LexA**	29	PCS	30	123	2	0.068966	0.068966
**IHF**	68	PCS	30	123	6	0.088235	0.088235
**NsrR**	32	PCS	30	123	2	0.0625	0.0625
**CRP**	183	PCS	30	123	5	0.027322	0.04065
**MarA**	21	PCS	30	123	1	0.047619	0.047619
**PhoP**	29	PCS	30	123	3	0.103448	0.103448
**ArcA**	58	PCS	30	123	3	0.051724	0.051724
**NarL**	29	PCS	30	123	1	0.034483	0.034483
**Fur**	45	PCS	30	123	1	0.022222	0.022222
**CpxR**	30	PCS	30	123	1	0.033333	0.033333
**SoxS**	21	PCS	30	123	0	0	0
**Fis**	60	PCS	35	144	7	0.116667	0.116667
**FNR**	95	PCS	35	144	4	0.042105	0.042105
**H-NS**	61	PCS	35	144	2	0.032787	0.032787
**FruR**	33	PCS	35	144	1	0.030303	0.030303
**Lrp**	30	PCS	35	144	0	0	0
**LexA**	29	PCS	35	144	2	0.068966	0.068966
**IHF**	68	PCS	35	144	8	0.117647	0.117647
**NsrR**	32	PCS	35	144	3	0.09375	0.09375
**CRP**	183	PCS	35	144	8	0.043716	0.055556
**MarA**	21	PCS	35	144	1	0.047619	0.047619
**PhoP**	29	PCS	35	144	3	0.103448	0.103448
**ArcA**	58	PCS	35	144	4	0.068966	0.068966
**NarL**	29	PCS	35	144	1	0.034483	0.034483
**Fur**	45	PCS	35	144	1	0.022222	0.022222
**CpxR**	30	PCS	35	144	1	0.033333	0.033333
**SoxS**	21	PCS	35	144	0	0	0
**Fis**	60	PCS	40	164	8	0.133333	0.133333
**FNR**	95	PCS	40	164	5	0.052632	0.052632
**H-NS**	61	PCS	40	164	2	0.032787	0.032787
**FruR**	33	PCS	40	164	3	0.090909	0.090909
**Lrp**	30	PCS	40	164	0	0	0
**LexA**	29	PCS	40	164	2	0.068966	0.068966
**IHF**	68	PCS	40	164	9	0.132353	0.132353
**NsrR**	32	PCS	40	164	3	0.09375	0.09375
**CRP**	183	PCS	40	164	12	0.065574	0.073171
**MarA**	21	PCS	40	164	1	0.047619	0.047619
**PhoP**	29	PCS	40	164	3	0.103448	0.103448
**ArcA**	58	PCS	40	164	5	0.086207	0.086207
**NarL**	29	PCS	40	164	2	0.068966	0.068966
**Fur**	45	PCS	40	164	1	0.022222	0.022222

**CpxR**	30	PCS	40	164	1	0.033333	0.033333
**SoxS**	21	PCS	40	164	0	0	0
**Fis**	60	PCS	45	185	8	0.133333	0.133333
**FNR**	95	PCS	45	185	6	0.063158	0.063158
**H-NS**	61	PCS	45	185	2	0.032787	0.032787
**FruR**	33	PCS	45	185	4	0.121212	0.121212
**Lrp**	30	PCS	45	185	0	0	0
**LexA**	29	PCS	45	185	2	0.068966	0.068966
**IHF**	68	PCS	45	185	12	0.176471	0.176471
**NsrR**	32	PCS	45	185	3	0.09375	0.09375
**CRP**	183	PCS	45	185	13	0.071038	0.071038
**MarA**	21	PCS	45	185	1	0.047619	0.047619
**PhoP**	29	PCS	45	185	3	0.103448	0.103448
**ArcA**	58	PCS	45	185	6	0.103448	0.103448
**NarL**	29	PCS	45	185	2	0.068966	0.068966
**Fur**	45	PCS	45	185	1	0.022222	0.022222
**CpxR**	30	PCS	45	185	1	0.033333	0.033333
**SoxS**	21	PCS	45	185	0	0	0
**Fis**	60	PCS	50	205	9	0.15	0.15
**FNR**	95	PCS	50	205	6	0.063158	0.063158
**H-NS**	61	PCS	50	205	4	0.065574	0.065574
**FruR**	33	PCS	50	205	4	0.121212	0.121212
**Lrp**	30	PCS	50	205	1	0.033333	0.033333
**LexA**	29	PCS	50	205	2	0.068966	0.068966
**IHF**	68	PCS	50	205	12	0.176471	0.176471
**NsrR**	32	PCS	50	205	3	0.09375	0.09375
**CRP**	183	PCS	50	205	16	0.087432	0.087432
**MarA**	21	PCS	50	205	1	0.047619	0.047619
**PhoP**	29	PCS	50	205	4	0.137931	0.137931
**ArcA**	58	PCS	50	205	6	0.103448	0.103448
**NarL**	29	PCS	50	205	2	0.068966	0.068966
**Fur**	45	PCS	50	205	2	0.044444	0.044444
**CpxR**	30	PCS	50	205	2	0.066667	0.066667
**SoxS**	21	PCS	50	205	0	0	0
**Fis**	60	PCS	55	226	11	0.183333	0.183333
**FNR**	95	PCS	55	226	6	0.063158	0.063158
**H-NS**	61	PCS	55	226	4	0.065574	0.065574
**FruR**	33	PCS	55	226	4	0.121212	0.121212
**Lrp**	30	PCS	55	226	1	0.033333	0.033333
**LexA**	29	PCS	55	226	2	0.068966	0.068966
**IHF**	68	PCS	55	226	12	0.176471	0.176471
**NsrR**	32	PCS	55	226	4	0.125	0.125
**CRP**	183	PCS	55	226	19	0.103825	0.103825
**MarA**	21	PCS	55	226	1	0.047619	0.047619
**PhoP**	29	PCS	55	226	4	0.137931	0.137931
**ArcA**	58	PCS	55	226	6	0.103448	0.103448
**NarL**	29	PCS	55	226	2	0.068966	0.068966

**Fur**	45	PCS	55	226	2	0.044444	0.044444
**CpxR**	30	PCS	55	226	2	0.066667	0.066667
**SoxS**	21	PCS	55	226	0	0	0
**Fis**	60	PCS	60	246	11	0.183333	0.183333
**FNR**	95	PCS	60	246	6	0.063158	0.063158
**H-NS**	61	PCS	60	246	4	0.065574	0.065574
**FruR**	33	PCS	60	246	5	0.151515	0.151515
**Lrp**	30	PCS	60	246	1	0.033333	0.033333
**LexA**	29	PCS	60	246	4	0.137931	0.137931
**IHF**	68	PCS	60	246	12	0.176471	0.176471
**NsrR**	32	PCS	60	246	4	0.125	0.125
**CRP**	183	PCS	60	246	20	0.10929	0.10929
**MarA**	21	PCS	60	246	1	0.047619	0.047619
**PhoP**	29	PCS	60	246	4	0.137931	0.137931
**ArcA**	58	PCS	60	246	6	0.103448	0.103448
**NarL**	29	PCS	60	246	2	0.068966	0.068966
**Fur**	45	PCS	60	246	2	0.044444	0.044444
**CpxR**	30	PCS	60	246	3	0.1	0.1
**SoxS**	21	PCS	60	246	0	0	0
**Fis**	60	PCS	65	265	11	0.183333	0.183333
**FNR**	95	PCS	65	265	6	0.063158	0.063158
**H-NS**	61	PCS	65	265	4	0.065574	0.065574
**FruR**	33	PCS	65	265	5	0.151515	0.151515
**Lrp**	30	PCS	65	265	1	0.033333	0.033333
**LexA**	29	PCS	65	265	4	0.137931	0.137931
**IHF**	68	PCS	65	265	12	0.176471	0.176471
**NsrR**	32	PCS	65	265	4	0.125	0.125
**CRP**	183	PCS	65	265	21	0.114754	0.114754
**MarA**	21	PCS	65	265	1	0.047619	0.047619
**PhoP**	29	PCS	65	265	4	0.137931	0.137931
**ArcA**	58	PCS	65	265	6	0.103448	0.103448
**NarL**	29	PCS	65	265	2	0.068966	0.068966
**Fur**	45	PCS	65	265	4	0.088889	0.088889
**CpxR**	30	PCS	65	265	3	0.1	0.1
**SoxS**	21	PCS	65	265	0	0	0
**Fis**	60	PCS	70	283	12	0.2	0.2
**FNR**	95	PCS	70	283	6	0.063158	0.063158
**H-NS**	61	PCS	70	283	4	0.065574	0.065574
**FruR**	33	PCS	70	283	6	0.181818	0.181818
**Lrp**	30	PCS	70	283	1	0.033333	0.033333
**LexA**	29	PCS	70	283	4	0.137931	0.137931
**IHF**	68	PCS	70	283	12	0.176471	0.176471
**NsrR**	32	PCS	70	283	4	0.125	0.125
**CRP**	183	PCS	70	283	23	0.125683	0.125683
**MarA**	21	PCS	70	283	2	0.095238	0.095238
**PhoP**	29	PCS	70	283	5	0.172414	0.172414
**ArcA**	58	PCS	70	283	7	0.12069	0.12069

**NarL**	29	PCS	70	283	2	0.068966	0.068966
**Fur**	45	PCS	70	283	4	0.088889	0.088889
**CpxR**	30	PCS	70	283	4	0.133333	0.133333
**SoxS**	21	PCS	70	283	1	0.047619	0.047619
**Fis**	60	PCS	75	305	15	0.25	0.25
**FNR**	95	PCS	75	305	7	0.073684	0.073684
**H-NS**	61	PCS	75	305	4	0.065574	0.065574
**FruR**	33	PCS	75	305	6	0.181818	0.181818
**Lrp**	30	PCS	75	305	1	0.033333	0.033333
**LexA**	29	PCS	75	305	4	0.137931	0.137931
**IHF**	68	PCS	75	305	14	0.205882	0.205882
**NsrR**	32	PCS	75	305	4	0.125	0.125
**CRP**	183	PCS	75	305	26	0.142077	0.142077
**MarA**	21	PCS	75	305	2	0.095238	0.095238
**PhoP**	29	PCS	75	305	5	0.172414	0.172414
**ArcA**	58	PCS	75	305	7	0.12069	0.12069
**NarL**	29	PCS	75	305	2	0.068966	0.068966
**Fur**	45	PCS	75	305	5	0.111111	0.111111
**CpxR**	30	PCS	75	305	4	0.133333	0.133333
**SoxS**	21	PCS	75	305	1	0.047619	0.047619
**Fis**	60	PCS	80	328	17	0.283333	0.283333
**FNR**	95	PCS	80	328	8	0.084211	0.084211
**H-NS**	61	PCS	80	328	4	0.065574	0.065574
**FruR**	33	PCS	80	328	7	0.212121	0.212121
**Lrp**	30	PCS	80	328	1	0.033333	0.033333
**LexA**	29	PCS	80	328	4	0.137931	0.137931
**IHF**	68	PCS	80	328	15	0.220588	0.220588
**NsrR**	32	PCS	80	328	4	0.125	0.125
**CRP**	183	PCS	80	328	29	0.15847	0.15847
**MarA**	21	PCS	80	328	2	0.095238	0.095238
**PhoP**	29	PCS	80	328	5	0.172414	0.172414
**ArcA**	58	PCS	80	328	8	0.137931	0.137931
**NarL**	29	PCS	80	328	2	0.068966	0.068966
**Fur**	45	PCS	80	328	8	0.177778	0.177778
**CpxR**	30	PCS	80	328	5	0.166667	0.166667
**SoxS**	21	PCS	80	328	1	0.047619	0.047619
**Fis**	60	PCS	85	350	17	0.283333	0.283333
**FNR**	95	PCS	85	350	8	0.084211	0.084211
**H-NS**	61	PCS	85	350	4	0.065574	0.065574
**FruR**	33	PCS	85	350	7	0.212121	0.212121
**Lrp**	30	PCS	85	350	1	0.033333	0.033333
**LexA**	29	PCS	85	350	4	0.137931	0.137931
**IHF**	68	PCS	85	350	15	0.220588	0.220588
**NsrR**	32	PCS	85	350	4	0.125	0.125
**CRP**	183	PCS	85	350	30	0.163934	0.163934
**MarA**	21	PCS	85	350	2	0.095238	0.095238
**PhoP**	29	PCS	85	350	5	0.172414	0.172414

**ArcA**	58	PCS	85	350	8	0.137931	0.137931
**NarL**	29	PCS	85	350	2	0.068966	0.068966
**Fur**	45	PCS	85	350	9	0.2	0.2
**CpxR**	30	PCS	85	350	5	0.166667	0.166667
**SoxS**	21	PCS	85	350	1	0.047619	0.047619
**Fis**	60	PCS	90	370	18	0.3	0.3
**FNR**	95	PCS	90	370	9	0.094737	0.094737
**H-NS**	61	PCS	90	370	4	0.065574	0.065574
**FruR**	33	PCS	90	370	7	0.212121	0.212121
**Lrp**	30	PCS	90	370	2	0.066667	0.066667
**LexA**	29	PCS	90	370	5	0.172414	0.172414
**IHF**	68	PCS	90	370	16	0.235294	0.235294
**NsrR**	32	PCS	90	370	4	0.125	0.125
**CRP**	183	PCS	90	370	31	0.169399	0.169399
**MarA**	21	PCS	90	370	2	0.095238	0.095238
**PhoP**	29	PCS	90	370	5	0.172414	0.172414
**ArcA**	58	PCS	90	370	9	0.155172	0.155172
**NarL**	29	PCS	90	370	3	0.103448	0.103448
**Fur**	45	PCS	90	370	10	0.222222	0.222222
**CpxR**	30	PCS	90	370	6	0.2	0.2
**SoxS**	21	PCS	90	370	1	0.047619	0.047619
**Fis**	60	PCS	95	389	18	0.3	0.3
**FNR**	95	PCS	95	389	10	0.105263	0.105263
**H-NS**	61	PCS	95	389	6	0.098361	0.098361
**FruR**	33	PCS	95	389	8	0.242424	0.242424
**Lrp**	30	PCS	95	389	2	0.066667	0.066667
**LexA**	29	PCS	95	389	5	0.172414	0.172414
**IHF**	68	PCS	95	389	16	0.235294	0.235294
**NsrR**	32	PCS	95	389	4	0.125	0.125
**CRP**	183	PCS	95	389	33	0.180328	0.180328
**MarA**	21	PCS	95	389	2	0.095238	0.095238
**PhoP**	29	PCS	95	389	5	0.172414	0.172414
**ArcA**	58	PCS	95	389	9	0.155172	0.155172
**NarL**	29	PCS	95	389	3	0.103448	0.103448
**Fur**	45	PCS	95	389	11	0.244444	0.244444
**CpxR**	30	PCS	95	389	6	0.2	0.2
**SoxS**	21	PCS	95	389	1	0.047619	0.047619
**Fis**	60	PCS	100	408	18	0.3	0.3
**FNR**	95	PCS	100	408	12	0.126316	0.126316
**H-NS**	61	PCS	100	408	7	0.114754	0.114754
**FruR**	33	PCS	100	408	8	0.242424	0.242424
**Lrp**	30	PCS	100	408	3	0.1	0.1
**LexA**	29	PCS	100	408	5	0.172414	0.172414
**IHF**	68	PCS	100	408	16	0.235294	0.235294
**NsrR**	32	PCS	100	408	4	0.125	0.125
**CRP**	183	PCS	100	408	34	0.185792	0.185792
**MarA**	21	PCS	100	408	3	0.142857	0.142857

**PhoP**	29	PCS	100	408	5	0.172414	0.172414
**ArcA**	58	PCS	100	408	9	0.155172	0.155172
**NarL**	29	PCS	100	408	3	0.103448	0.103448
**Fur**	45	PCS	100	408	13	0.288889	0.288889
**CpxR**	30	PCS	100	408	6	0.2	0.2
**SoxS**	21	PCS	100	408	2	0.095238	0.095238

Table S6. Evaluation of the predicted regulons based on co-expression modules.

cluID	bicSmall	bicPosSmall	bicSum	bicPosSum	bicType	cluType
1	5.77E-05	1.26E-04	27.426	10.712	default	bbs_cluster
2	7.62E-03	9.83E-04	0	3.007	default	bbs_cluster
3	6.38E-03	6.18E-03	0	0	default	bbs_cluster
4	6.05E-07	6.26E-08	57.896	56.63	default	bbs_cluster
5	8.71E-09	6.22E-10	75.777	73.943	default	bbs_cluster
6	3.19E-05	1.82E-05	87.177	96.035	default	bbs_cluster
7	5.00E-15	3.68E-15	233.938	239.834	default	bbs_cluster
8	3.13E-07	1.34E-07	233.136	299.333	default	bbs_cluster
9	1.83E-03	5.25E-04	0	6.491	default	bbs_cluster
10	3.99E-03	1.03E-03	0	0	default	bbs_cluster
11	2.97E-02	1.32E-02	0	0	default	bbs_cluster
12	7.75E-14	7.75E-14	2264.211	1873.471	default	bbs_cluster
13	4.71E-08	4.02E-08	156.332	158.099	default	bbs_cluster
14	6.84E-08	6.84E-08	1093.024	1009.848	default	bbs_cluster
15	2.77E-08	9.28E-09	22.396	44.398	default	bbs_cluster
16	8.36E-03	7.33E-05	0	7.271	default	bbs_cluster
17	3.68E-05	3.97E-03	7.779	0	default	bbs_cluster
18	4.49E-06	4.49E-06	82.489	86.142	default	bbs_cluster
19	5.25E-05	5.25E-05	11.397	11.639	default	bbs_cluster
20	1.66E-03	8.45E-04	0	9.131	default	bbs_cluster
21	3.00E-02	2.11E-02	0	0	default	bbs_cluster
22	2.83E-06	2.83E-06	113.208	145.575	default	bbs_cluster
23	6.01E-05	2.47E-05	4.221	4.607	default	bbs_cluster
24	3.37E-04	6.13E-05	3.472	8.324	default	bbs_cluster
25	3.17E-04	5.78E-07	3.499	73.832	default	bbs_cluster
26	1.13E-06	1.13E-06	348.5	290.099	default	bbs_cluster
27	5.17E-03	9.83E-04	0	3.008	default	bbs_cluster
28	7.26E-04	6.99E-04	6.2	9.249	default	bbs_cluster
29	8.53E-04	2.63E-03	3.069	0	default	bbs_cluster
30	6.00E-07	1.41E-07	154.737	170.988	default	bbs_cluster
31	7.07E-05	3.38E-04	4.151	3.471	default	bbs_cluster
32	3.08E-02	1.30E-02	0	0	default	bbs_cluster
33	1.51E-03	1.39E-03	0	0	default	bbs_cluster
34	4.18E-04	4.18E-04	6.685	9.697	default	bbs_cluster
35	1.83E-08	1.83E-08	69.471	69.471	default	bbs_cluster
36	4.05E-05	1.05E-05	11.809	16.506	default	bbs_cluster
37	2.53E-04	2.25E-04	9.934	10.119	default	bbs_cluster
38	3.13E-05	8.47E-05	59.923	17.057	default	bbs_cluster

39	1.53E-02	1.42E-03	0	0	default	bbs_cluster
40	9.57E-06	9.57E-06	70.213	70.213	default	bbs_cluster
41	4.23E-06	1.77E-06	17.717	22.197	default	bbs_cluster
42	1.50E-04	1.28E-05	6.941	52.174	default	bbs_cluster
43	7.63E-05	3.15E-05	4.117	4.501	default	bbs_cluster
44	8.72E-04	3.82E-04	3.06	3.418	default	bbs_cluster
45	2.15E-19	3.35E-20	399.222	427.45	default	bbs_cluster
46	3.60E-03	3.13E-03	0	0	default	bbs_cluster
47	2.62E-02	4.10E-04	0	3.387	default	bbs_cluster
48	8.18E-02	2.35E-02	0	0	default	bbs_cluster
49	2.37E-04	7.03E-05	6.734	57.512	default	bbs_cluster
50	2.12E-03	1.51E-03	0	0	default	bbs_cluster
51	1.76E-04	1.11E-04	29.521	39.736	default	bbs_cluster
52	1.02E-03	2.03E-04	0	3.693	default	bbs_cluster
53	3.29E-05	1.02E-04	38.02	33.54	default	bbs_cluster
54	5.06E-05	5.63E-06	184.474	295.641	default	bbs_cluster
55	1.26E-03	4.91E-06	0	16.14	default	bbs_cluster
56	1.63E-13	1.63E-13	264.153	259.881	default	bbs_cluster
57	5.14E-03	1.56E-04	0	10.667	default	bbs_cluster
58	2.89E-06	2.89E-06	66.888	85.899	default	bbs_cluster
59	8.68E-04	7.93E-04	3.061	3.101	default	bbs_cluster
60	8.87E-06	6.58E-06	131.124	140.389	default	bbs_cluster
61	2.50E-04	7.83E-06	10.421	43.338	default	bbs_cluster
62	9.52E-03	3.37E-04	0	3.472	default	bbs_cluster
63	4.83E-09	3.89E-09	1013.829	928.86	default	bbs_cluster
64	9.77E-08	9.77E-08	92.267	95.765	default	bbs_cluster
65	2.54E-05	9.73E-06	13.121	14.343	default	bbs_cluster
66	2.74E-04	4.41E-05	6.926	31.991	default	bbs_cluster
67	7.15E-03	2.42E-03	0	0	default	bbs_cluster
68	3.16E-08	3.16E-08	238.388	239.931	default	bbs_cluster
69	3.10E-07	2.48E-07	148.236	144.329	default	bbs_cluster
70	3.86E-03	3.86E-03	0	0	default	bbs_cluster
71	8.46E-05	4.57E-06	35.08	53.032	default	bbs_cluster
72	2.40E-05	3.90E-06	25.042	67.352	default	bbs_cluster
73	6.43E-07	1.07E-05	103.667	106.112	default	bbs_cluster
74	1.73E-07	6.55E-10	275.628	416.218	default	bbs_cluster
75	7.01E-04	7.01E-04	3.154	6.309	default	bbs_cluster
76	1.77E-03	2.76E-03	0	0	default	bbs_cluster
77	9.11E-03	9.11E-03	0	0	default	bbs_cluster
78	1.23E-04	1.23E-04	3.911	7.045	default	bbs_cluster
79	2.45E-02	1.53E-02	0	0	default	bbs_cluster
80	1.10E-01	3.28E-02	0	0	default	bbs_cluster
81	1.11E-03	1.55E-04	0	10.018	default	bbs_cluster
82	9.01E-06	9.01E-06	68.626	46.645	default	bbs_cluster
83	3.34E-03	2.75E-03	0	0	default	bbs_cluster
84	2.16E-11	7.00E-12	1835.73	1582.336	default	bbs_cluster
85	1.48E-04	8.74E-06	7.529	60.512	default	bbs_cluster
86	6.31E-06	9.79E-06	76.738	37.353	default	bbs_cluster
87	3.69E-08	2.64E-08	154.497	152.737	default	bbs_cluster

88	3.82E-06	2.08E-08	101.404	150.055	default	bbs_cluster
89	1.36E-04	8.96E-05	7.491	7.955	default	bbs_cluster
90	1.03E-05	1.03E-05	88.939	84.083	default	bbs_cluster
91	5.66E-06	1.25E-06	28.769	34.901	default	bbs_cluster
92	7.38E-04	4.46E-03	3.132	0	default	bbs_cluster
93	1.58E-04	8.09E-05	10.489	17.662	default	bbs_cluster
94	5.42E-03	5.42E-03	0	0	default	bbs_cluster
95	1.31E-03	1.31E-03	0	0	default	bbs_cluster
96	5.62E-03	2.08E-03	0	0	default	bbs_cluster
97	6.25E-05	6.25E-05	58.215	62.154	default	bbs_cluster
98	6.52E-13	6.52E-13	1571.783	1427.768	default	bbs_cluster
99	1.43E-03	1.25E-03	0	0	default	bbs_cluster
100	2.92E-03	2.14E-03	0	0	default	bbs_cluster
1	3.00E-02	4.34E-02	0	0	default	edge_1_300
2	1.64E-03	1.29E-04	0	3.89	default	edge_1_300
3	3.80E-02	2.27E-02	0	0	default	edge_1_300
4	1.85E-03	1.85E-03	0	0	default	edge_1_300
5	8.50E-07	3.08E-07	58.101	55.337	default	edge_1_300
6	1.02E-13	1.02E-13	138.568	138.568	default	edge_1_300
7	4.28E-07	3.80E-07	133.738	143.395	default	edge_1_300
8	3.99E-03	1.75E-03	0	0	default	edge_1_300
9	1.70E-06	2.43E-07	35.921	42.506	default	edge_1_300
10	1.48E-02	5.45E-03	0	0	default	edge_1_300
11	1.92E-02	8.43E-03	0	0	default	edge_1_300
12	2.34E-03	2.34E-03	0	0	default	edge_1_300
13	6.92E-06	6.32E-06	104.747	105.684	default	edge_1_300
14	2.02E-02	3.62E-03	0	0	default	edge_1_300
15	1.36E-02	3.61E-03	0	0	default	edge_1_300
16	6.92E-07	6.92E-07	132.125	133.193	default	edge_1_300
17	1.96E-01	3.35E-02	0	0	default	edge_1_300
18	2.09E-05	2.09E-05	7.919	7.919	default	edge_1_300
19	7.36E-06	7.36E-06	73.012	73.012	default	edge_1_300
20	1.09E-09	4.12E-10	25.583	27.777	default	edge_1_300
21	6.02E-02	3.61E-02	0	0	default	edge_1_300
22	4.02E-05	1.89E-05	46.917	47.83	default	edge_1_300
23	6.74E-02	4.05E-02	0	0	default	edge_1_300
24	5.21E-02	5.17E-03	0	0	default	edge_1_300
25	3.75E-08	1.42E-08	550.976	642.9	default	edge_1_300
26	1.36E-02	8.69E-03	0	0	default	edge_1_300
27	4.55E-02	2.72E-02	0	0	default	edge_1_300
28	8.09E-04	8.09E-04	3.092	3.092	default	edge_1_300
29	7.41E-03	7.41E-03	0	0	default	edge_1_300
30	1.32E-08	6.77E-09	32.352	66.202	default	edge_1_300
31	5.60E-03	5.28E-03	0	0	default	edge_1_300
32	1.15E-02	2.24E-03	0	0	default	edge_1_300
33	1.73E-07	6.24E-08	68.229	64.686	default	edge_1_300
34	1.70E-02	1.53E-03	0	0	default	edge_1_300
35	3.09E-02	3.09E-02	0	0	default	edge_1_300
36	3.82E-03	1.91E-04	0	3.719	default	edge_1_300

37	1.12E-03	1.12E-03	0	0	default	edge_1_300
38	6.52E-04	6.52E-04	43.811	43.811	default	edge_1_300
39	5.74E-03	4.58E-04	0	3.339	default	edge_1_300
40	8.44E-03	1.94E-03	0	0	default	edge_1_300
41	7.94E-07	2.24E-07	74.774	68.683	default	edge_1_300
42	1.72E-02	8.88E-03	0	0	default	edge_1_300
43	1.54E-02	9.15E-03	0	0	default	edge_1_300
44	1.20E-02	2.59E-03	0	0	default	edge_1_300
45	1.30E-08	1.30E-08	115.197	115.197	default	edge_1_300
46	7.56E-04	5.35E-04	3.121	3.272	default	edge_1_300
47	6.24E-05	5.32E-05	52.502	53.056	default	edge_1_300
48	6.74E-02	4.05E-02	0	0	default	edge_1_300
49	5.28E-02	3.17E-02	0	0	default	edge_1_300
50	2.24E-03	2.24E-03	0	0	default	edge_1_300
51	3.00E-24	3.00E-24	5298.484	4035.556	default	edge_1_300
52	2.44E-11	9.10E-12	43.349	49.469	default	edge_1_300
53	1.10E-02	8.33E-03	0	0	default	edge_1_300
54	6.89E-08	3.36E-08	638.926	716.951	default	edge_1_300
55	5.10E-03	5.10E-03	0	0	default	edge_1_300
56	1.14E-02	8.16E-03	0	0	default	edge_1_300
57	2.33E-05	9.97E-05	229.565	139.886	default	edge_1_300
58	2.96E-07	2.96E-07	659.427	648.186	default	edge_1_300
59	8.92E-06	8.49E-06	15.887	16.096	default	edge_1_300
60	2.58E-08	2.58E-08	110.739	110.739	default	edge_1_300
61	1.53E-03	1.28E-03	0	0	default	edge_1_300
62	1.48E-02	1.29E-02	0	0	default	edge_1_300
63	2.19E-02	7.73E-03	0	0	default	edge_1_300
64	6.93E-13	6.93E-13	330.315	323.343	default	edge_1_300
65	2.85E-06	2.59E-06	135.145	130.921	default	edge_1_300
66	1.54E-03	2.98E-03	0	0	default	edge_1_300
67	1.54E-02	9.15E-03	0	0	default	edge_1_300
68	5.28E-02	3.17E-02	0	0	default	edge_1_300
69	3.29E-05	3.06E-05	99.124	100.053	default	edge_1_300
70	3.05E-02	1.82E-02	0	0	default	edge_1_300
71	1.02E-07	8.99E-08	104.523	105.465	default	edge_1_300
72	8.85E-10	8.85E-10	132.059	132.059	default	edge_1_300
73	3.27E-03	1.60E-03	0	0	default	edge_1_300
74	2.38E-04	8.96E-05	3.623	4.048	default	edge_1_300
75	4.56E-05	1.24E-05	27.219	33.769	default	edge_1_300
76	4.55E-02	2.72E-02	0	0	default	edge_1_300
77	3.05E-02	1.82E-02	0	0	default	edge_1_300
78	1.20E-03	9.88E-04	0	3.005	default	edge_1_300
79	3.80E-02	2.27E-02	0	0	default	edge_1_300
80	6.02E-02	3.61E-02	0	0	default	edge_1_300
81	1.09E-03	3.38E-03	0	0	default	edge_1_300
82	5.60E-10	4.67E-10	238.991	292.808	default	edge_1_300
83	2.46E-02	1.06E-03	0	0	default	edge_1_300
84	8.09E-04	6.82E-04	6.149	12.39	default	edge_1_300
85	6.02E-02	3.61E-02	0	0	default	edge_1_300

86	1.76E-03	1.76E-03	0	0	default	edge_1_300
87	1.77E-10	1.75E-09	261.296	254.052	default	edge_1_300
88	6.04E-03	1.04E-03	0	0	default	edge_1_300
89	3.32E-07	1.90E-07	57.227	51.116	default	edge_1_300
90	3.61E-02	2.80E-02	0	0	default	edge_1_300
91	3.96E-03	3.96E-03	0	0	default	edge_1_300
92	3.30E-05	1.22E-05	37.603	38.218	default	edge_1_300
93	6.85E-02	9.60E-03	0	0	default	edge_1_300
94	8.47E-04	6.73E-05	21.287	44.431	default	edge_1_300
95	3.98E-02	3.61E-02	0	0	default	edge_1_300
96	7.13E-04	3.88E-04	12.375	19.006	default	edge_1_300
97	3.99E-03	4.58E-04	0	3.339	default	edge_1_300
98	1.46E-02	1.19E-02	0	0	default	edge_1_300
99	8.33E-03	8.13E-03	0	0	default	edge_1_300
100	3.97E-03	2.31E-04	0	3.636	default	edge_1_300
1	7.05E-07	6.25E-07	97.048	98.088	default	gfr
2	6.17E-04	5.55E-04	6.236	6.336	default	gfr
3	2.15E-06	1.90E-06	119.041	116.668	default	gfr
4	5.22E-20	5.22E-20	3282.415	2833.535	default	gfr
5	8.89E-02	5.37E-02	0	0	default	gfr
6	4.17E-08	2.74E-07	162.007	156.179	default	gfr
7	2.84E-07	2.56E-07	37.59	30.88	default	gfr
8	6.09E-06	5.37E-06	57.695	58.515	default	gfr
9	1.64E-14	1.03E-14	13.785	13.987	default	gfr
10	3.03E-06	2.87E-06	188.761	168.453	default	gfr
11	9.29E-04	9.29E-04	3.032	3.032	default	gfr
12	4.08E-06	3.44E-06	21.113	14.981	default	gfr
13	2.61E-15	1.12E-15	1961.498	1490.432	default	gfr
14	4.73E-06	4.73E-06	140.243	137.429	default	gfr
15	2.81E-04	2.81E-04	3.551	3.551	default	gfr
16	5.45E-57	5.45E-57	9319.857	7675.902	default	gfr
17	1.82E-07	5.11E-08	66.154	62.256	default	gfr
18	3.68E-03	3.68E-03	0	0	default	gfr
19	1.02E-13	1.02E-13	1726.958	1525.92	default	gfr
20	3.88E-15	3.42E-15	163.889	162.285	default	gfr
21	3.68E-19	3.05E-19	483.63	471.862	default	gfr
22	8.77E-04	7.76E-05	3.057	4.11	default	gfr
23	4.49E-03	4.49E-03	0	0	default	gfr
24	2.79E-05	2.35E-05	27.83	43.234	default	gfr
25	4.25E-04	3.28E-04	9.808	10.038	default	gfr
26	3.08E-06	2.72E-06	94.515	101.827	default	gfr
27	7.38E-06	7.38E-06	206.551	188.532	default	gfr
28	1.59E-07	1.59E-07	555.794	493.46	default	gfr
29	1.49E-04	1.49E-04	48.146	49.903	default	gfr
30	1.40E-02	1.05E-02	0	0	default	gfr
31	1.26E-02	1.26E-02	0	0	default	gfr
32	2.50E-03	2.50E-03	0	0	default	gfr
33	6.35E-10	6.35E-10	132.481	132.481	default	gfr
34	8.21E-06	8.21E-06	483.765	380.931	default	gfr

35	5.70E-03	5.70E-03	0	0	default	gfr
36	3.45E-03	3.29E-03	0	0	default	gfr
37	1.76E-03	1.76E-03	0	0	default	gfr
38	1.60E-03	1.17E-03	0	0	default	gfr
39	6.74E-02	4.05E-02	0	0	default	gfr
40	5.12E-03	1.84E-03	0	0	default	gfr
41	1.16E-05	9.56E-06	126.415	131.168	default	gfr
42	1.27E-04	1.27E-04	3.896	3.896	default	gfr
43	2.82E-07	2.82E-07	716.272	548.748	default	gfr
44	6.15E-03	5.56E-03	0	0	default	gfr
45	7.61E-05	1.86E-04	39.827	36.015	default	gfr
46	4.56E-07	4.56E-07	280.107	242.888	default	gfr
47	3.29E-05	3.29E-05	284.774	290.59	default	gfr
48	4.01E-05	7.83E-05	110.473	93.512	default	gfr
49	1.77E-10	1.75E-09	223.693	215.835	default	gfr
50	9.70E-07	8.14E-07	328.469	273.471	default	gfr
51	3.85E-02	1.14E-02	0	0	default	gfr
52	0.00E+00	0.00E+00	102.521	102.521	default	gfr
53	6.11E-04	1.59E-03	12.582	0	default	gfr
54	1.54E-10	1.41E-10	19.278	19.316	default	gfr
55	1.20E-03	1.20E-03	0	0	default	gfr
56	2.10E-04	2.10E-04	3.678	3.678	default	gfr
57	3.58E-07	2.98E-07	10.515	10.758	default	gfr
58	2.83E-04	1.96E-04	35.156	41.076	default	gfr
59	7.31E-03	1.75E-03	0	0	default	gfr
60	9.11E-04	9.11E-04	3.04	3.04	default	gfr
61	1.53E-05	1.53E-05	36.74	22.212	default	gfr
62	3.89E-03	3.89E-03	0	0	default	gfr
63	5.28E-02	3.17E-02	0	0	default	gfr
64	9.37E-04	3.26E-04	3.028	9.523	default	gfr
65	1.10E-02	6.77E-03	0	0	default	gfr
66	3.23E-03	5.30E-04	0	3.276	default	gfr
67	3.12E-03	2.49E-03	0	0	default	gfr
68	1.21E-03	1.21E-03	0	0	default	gfr
69	2.70E-03	2.70E-03	0	0	default	gfr
70	2.04E-03	1.94E-03	0	0	default	gfr
71	1.43E-06	4.06E-07	59.737	78.153	default	gfr
72	4.55E-02	2.72E-02	0	0	default	gfr
73	6.00E-07	6.00E-07	90.286	90.286	default	gfr
74	4.34E-13	3.42E-13	267.294	261.171	default	gfr
75	5.28E-02	3.17E-02	0	0	default	gfr
76	5.69E-07	1.05E-07	222.598	275.949	default	gfr
77	2.25E-04	2.25E-04	13.865	20.123	default	gfr
78	1.78E-05	3.78E-06	7.857	9.016	default	gfr
79	9.90E-08	9.90E-08	1473.32	1195.67	default	gfr
80	1.66E-03	8.00E-04	0	15.41	default	gfr
81	2.88E-03	2.88E-03	0	0	default	gfr
82	1.37E-35	1.37E-35	440.786	440.786	default	gfr
83	6.81E-06	6.81E-06	91.6	122.245	default	gfr

84	4.55E-02	2.72E-02	0	0	default	gfr
85	1.29E-03	1.23E-03	0	0	default	gfr
86	5.13E-02	2.60E-03	0	0	default	gfr
87	2.62E-02	1.23E-02	0	0	default	gfr
88	8.18E-02	4.93E-02	0	0	default	gfr
89	1.87E-03	1.87E-03	0	0	default	gfr
90	2.48E-03	1.51E-03	0	0	default	gfr
91	1.24E-11	1.24E-11	425.044	518.683	default	gfr
92	4.34E-09	3.62E-09	35.686	29.521	default	gfr
93	9.36E-06	1.14E-06	218.18	202.602	default	gfr
94	1.54E-02	2.27E-02	0	0	default	gfr
95	6.98E-03	6.39E-03	0	0	default	gfr
96	4.08E-04	2.99E-04	28.492	66.47	default	gfr
97	1.03E-04	3.35E-04	58.924	50.417	default	gfr
98	2.43E-05	1.99E-05	47.145	47.594	default	gfr
99	5.11E-04	5.11E-04	12.556	18.807	default	gfr
100	3.98E-25	3.98E-25	357.874	357.874	default	gfr
1	5.28E-02	3.60E-03	0	0	default	pcs
2	3.31E-06	3.31E-06	354.028	410.366	default	pcs
3	2.42E-02	8.05E-03	0	0	default	pcs
4	6.83E-04	6.47E-04	9.29	12.471	default	pcs
5	2.54E-03	1.17E-03	0	0	default	pcs
6	4.44E-04	7.56E-06	22.266	73.225	default	pcs
7	5.23E-05	4.86E-05	93.917	94.804	default	pcs
8	1.76E-03	9.59E-04	0	3.018	default	pcs
9	1.34E-03	9.83E-04	0	3.008	default	pcs
10	4.08E-03	2.72E-03	0	0	default	pcs
11	4.08E-03	2.64E-03	0	0	default	pcs
12	7.80E-03	3.59E-03	0	0	default	pcs
13	5.58E-05	4.22E-05	25.88	29.541	default	pcs
14	8.43E-03	8.27E-03	0	0	default	pcs
15	7.87E-05	7.87E-05	7.881	7.881	default	pcs
16	1.17E-10	8.09E-11	9.934	10.092	default	pcs
17	3.44E-06	3.44E-06	5.464	5.464	default	pcs
18	3.05E-02	1.82E-02	0	0	default	pcs
19	5.11E-04	5.11E-04	22.575	28.697	default	pcs
20	1.10E-03	9.11E-04	0	3.04	default	pcs
21	1.92E-02	5.63E-03	0	0	default	pcs
22	3.77E-08	3.77E-08	84.813	61.987	default	pcs
23	2.77E-11	2.77E-11	154.608	154.608	default	pcs
24	3.80E-02	2.27E-02	0	0	default	pcs
25	4.55E-02	2.72E-02	0	0	default	pcs
26	6.02E-02	3.61E-02	0	0	default	pcs
27	1.47E-03	1.40E-03	0	0	default	pcs
28	1.45E-02	1.45E-02	0	0	default	pcs
29	3.80E-02	2.27E-02	0	0	default	pcs
30	5.38E-04	5.38E-04	3.269	3.269	default	pcs
31	2.54E-03	2.43E-03	0	0	default	pcs
32	7.46E-02	4.49E-02	0	0	default	pcs

33	1.15E-02	7.37E-03	0	0	default	pcs
34	2.88E-03	9.83E-04	0	3.008	default	pcs
35	3.90E-03	2.49E-03	0	0	default	pcs
36	6.37E-04	8.74E-05	3.196	24.734	default	pcs
37	1.47E-03	1.47E-03	0	0	default	pcs
38	1.29E-02	4.74E-03	0	0	default	pcs
39	3.94E-02	1.93E-02	0	0	default	pcs
40	1.10E-05	1.10E-05	643.316	503.14	default	pcs
41	8.42E-04	8.42E-04	3.075	3.075	default	pcs
42	1.95E-04	1.95E-04	22.284	22.54	default	pcs
43	1.08E-03	1.08E-03	0	0	default	pcs
44	1.53E-04	1.49E-03	7.362	0	default	pcs
45	6.20E-03	3.74E-04	0	3.427	default	pcs
46	1.59E-03	1.59E-03	0	0	default	pcs
47	3.34E-02	1.40E-02	0	0	default	pcs
48	8.36E-03	3.04E-03	0	0	default	pcs
49	1.36E-05	1.30E-05	45.62	27.597	default	pcs
50	2.14E-04	2.14E-04	51.292	74.233	default	pcs
51	1.90E-04	1.66E-04	25.01	25.069	default	pcs
52	1.18E-03	6.43E-04	0	3.192	default	pcs
53	6.24E-05	5.32E-05	52.502	53.056	default	pcs
54	5.19E-02	5.12E-03	0	0	default	pcs
55	4.26E-03	8.03E-03	0	0	default	pcs
56	4.04E-07	1.23E-07	39.239	37.391	default	pcs
57	1.54E-04	1.38E-04	3.814	3.861	default	pcs
58	4.66E-03	4.27E-03	0	0	default	pcs
59	3.66E-03	3.66E-03	0	0	default	pcs
60	2.26E-03	1.10E-03	0	0	default	pcs
61	1.15E-05	2.43E-06	8.181	12.401	default	pcs
62	5.28E-02	2.80E-02	0	0	default	pcs
63	3.80E-02	2.27E-02	0	0	default	pcs
64	1.38E-03	1.32E-03	0	0	default	pcs
65	2.43E-03	6.22E-04	0	3.206	default	pcs
66	1.05E-02	4.74E-03	0	0	default	pcs
67	1.10E-05	1.10E-05	4.959	4.959	default	pcs
68	6.00E-05	4.90E-05	17.668	17.944	default	pcs
69	6.54E-04	6.54E-04	12.439	15.544	default	pcs
70	2.74E-04	7.45E-05	3.562	10.832	default	pcs
71	7.10E-03	1.57E-03	0	0	default	pcs
72	1.51E-05	1.51E-05	76.003	32.527	default	pcs
73	6.19E-07	6.19E-07	199.55	187.798	default	pcs
74	4.78E-06	4.36E-06	173.535	159.31	default	pcs
75	3.64E-05	1.32E-05	53.767	54.586	default	pcs
76	1.35E-07	1.22E-07	174.484	168.959	default	pcs
77	1.25E-06	1.10E-06	71.466	72.433	default	pcs
78	2.36E-04	2.08E-04	22.746	13.2	default	pcs
79	4.55E-02	2.72E-02	0	0	default	pcs
80	6.02E-02	3.61E-02	0	0	default	pcs
81	4.04E-03	4.04E-03	0	0	default	pcs

82	3.18E-05	4.63E-05	59.469	46.929	default	pcs
83	2.14E-03	2.14E-03	0	0	default	pcs
84	8.35E-03	5.13E-03	0	0	default	pcs
85	1.54E-03	4.99E-03	0	0	default	pcs
86	5.69E-04	5.69E-04	3.245	3.245	default	pcs
87	2.66E-03	2.66E-03	0	0	default	pcs
88	1.37E-06	1.37E-06	118.222	119.165	default	pcs
89	3.05E-02	8.64E-03	0	0	default	pcs
90	4.27E-03	1.47E-03	0	0	default	pcs
91	6.68E-03	1.46E-04	0	3.836	default	pcs
92	5.12E-04	4.79E-04	9.603	9.696	default	pcs
93	2.36E-08	1.87E-08	277.359	240.778	default	pcs
94	5.13E-03	4.83E-03	0	0	default	pcs
95	1.26E-03	1.26E-03	0	0	default	pcs
96	4.06E-02	1.52E-02	0	0	default	pcs
97	3.43E-04	3.43E-04	6.581	6.608	default	pcs
98	4.48E-04	2.13E-04	16.308	25.009	default	pcs
99	1.77E-10	1.75E-09	223.693	215.835	default	pcs
100	3.00E-11	3.00E-11	149.623	149.623	default	pcs
1	3.69E-08	3.69E-08	30.777	7.432	loose	bbs_cluster
2	7.97E-03	7.97E-03	0	0	loose	bbs_cluster
3	4.88E-03	2.00E-02	0	0	loose	bbs_cluster
4	5.95E-07	1.51E-07	44.534	47.736	loose	bbs_cluster
5	1.53E-07	1.06E-08	56.757	60.941	loose	bbs_cluster
6	1.98E-05	1.98E-05	96.259	103.239	loose	bbs_cluster
7	6.33E-19	2.81E-19	292.582	300.121	loose	bbs_cluster
8	3.13E-07	1.89E-07	219.137	280.211	loose	bbs_cluster
9	2.89E-03	8.14E-04	0	3.089	loose	bbs_cluster
10	2.00E-03	1.68E-03	0	0	loose	bbs_cluster
11	7.46E-02	3.02E-02	0	0	loose	bbs_cluster
12	7.75E-14	7.75E-14	2250.626	1738.177	loose	bbs_cluster
13	9.85E-08	6.88E-08	160.888	163.315	loose	bbs_cluster
14	8.05E-10	8.05E-10	1017.369	907.939	loose	bbs_cluster
15	2.03E-08	1.31E-08	32.518	63.161	loose	bbs_cluster
16	9.01E-03	4.57E-04	0	3.34	loose	bbs_cluster
17	4.04E-05	4.04E-05	8.359	4.393	loose	bbs_cluster
18	4.49E-06	4.49E-06	84.093	88.463	loose	bbs_cluster
19	3.68E-05	2.88E-05	22.987	26.537	loose	bbs_cluster
20	6.67E-04	2.02E-05	3.176	11.557	loose	bbs_cluster
21	9.70E-03	9.32E-03	0	0	loose	bbs_cluster
22	2.45E-06	2.45E-06	129.16	162.736	loose	bbs_cluster
23	4.67E-05	3.28E-05	4.331	7.525	loose	bbs_cluster
24	3.11E-04	4.99E-05	6.935	24.292	loose	bbs_cluster
25	3.17E-04	2.85E-08	3.499	104.294	loose	bbs_cluster
26	1.13E-06	1.13E-06	368.558	296.404	loose	bbs_cluster
27	5.93E-03	6.82E-04	0	6.174	loose	bbs_cluster
28	1.03E-03	9.00E-04	0	6.048	loose	bbs_cluster
29	1.12E-03	2.14E-03	0	0	loose	bbs_cluster
30	2.41E-05	7.65E-06	170.478	176.408	loose	bbs_cluster

31	9.46E-03	1.12E-02	0	0	loose	bbs_cluster
32	4.39E-03	1.16E-03	0	0	loose	bbs_cluster
33	1.24E-03	1.15E-03	0	0	loose	bbs_cluster
34	4.55E-04	4.55E-04	6.648	6.648	loose	bbs_cluster
35	2.16E-08	2.16E-08	72.638	79.305	loose	bbs_cluster
36	1.25E-04	3.39E-05	24.05	29.144	loose	bbs_cluster
37	1.21E-04	1.21E-04	6.951	7.028	loose	bbs_cluster
38	1.85E-05	8.47E-05	58.295	17.392	loose	bbs_cluster
39	1.20E-02	1.05E-02	0	0	loose	bbs_cluster
40	9.57E-06	9.57E-06	68.482	68.482	loose	bbs_cluster
41	5.36E-05	2.21E-05	17.925	21.702	loose	bbs_cluster
42	1.20E-03	3.02E-05	0	64.9	loose	bbs_cluster
43	3.15E-05	2.86E-05	8.727	12.531	loose	bbs_cluster
44	1.12E-04	3.11E-04	17.767	20.403	loose	bbs_cluster
45	1.10E-17	6.11E-18	378.803	410.206	loose	bbs_cluster
46	2.29E-04	1.85E-04	6.786	6.955	loose	bbs_cluster
47	1.13E-02	2.02E-04	0	3.695	loose	bbs_cluster
48	1.36E-02	1.33E-02	0	0	loose	bbs_cluster
49	2.37E-04	2.37E-04	6.734	44.438	loose	bbs_cluster
50	1.99E-03	1.51E-03	0	0	loose	bbs_cluster
51	3.74E-04	2.26E-04	35.221	82.233	loose	bbs_cluster
52	6.70E-04	5.69E-04	3.174	3.245	loose	bbs_cluster
53	2.24E-05	7.71E-05	22.482	21.354	loose	bbs_cluster
54	4.58E-05	4.24E-06	131.29	300.24	loose	bbs_cluster
55	4.13E-06	2.82E-06	15.881	63.751	loose	bbs_cluster
56	4.87E-13	4.87E-13	241.46	255.811	loose	bbs_cluster
57	3.31E-03	1.74E-03	0	0	loose	bbs_cluster
58	2.89E-06	2.89E-06	71.411	79.035	loose	bbs_cluster
59	2.14E-03	1.80E-03	0	0	loose	bbs_cluster
60	4.54E-05	2.77E-05	101.616	120.839	loose	bbs_cluster
61	3.98E-04	1.84E-05	9.536	58.6	loose	bbs_cluster
62	9.16E-03	1.55E-03	0	0	loose	bbs_cluster
63	5.63E-11	5.63E-11	1029.576	871.824	loose	bbs_cluster
64	1.21E-07	1.21E-07	94.33	94.95	loose	bbs_cluster
65	5.39E-05	7.34E-06	18.096	33.044	loose	bbs_cluster
66	6.11E-04	4.90E-05	3.214	24.115	loose	bbs_cluster
67	8.26E-03	1.89E-03	0	0	loose	bbs_cluster
68	4.20E-08	4.20E-08	220.999	223.257	loose	bbs_cluster
69	2.64E-07	2.29E-07	128.783	131.923	loose	bbs_cluster
70	3.86E-03	3.86E-03	0	0	loose	bbs_cluster
71	1.28E-05	4.57E-06	63.749	113.747	loose	bbs_cluster
72	5.09E-07	1.41E-06	55.929	126.365	loose	bbs_cluster
73	4.28E-06	2.88E-06	44.224	70.702	loose	bbs_cluster
74	1.16E-07	3.04E-08	246.11	383.298	loose	bbs_cluster
75	7.01E-04	1.84E-04	12.279	6.891	loose	bbs_cluster
76	3.05E-03	3.05E-03	0	0	loose	bbs_cluster
77	9.11E-03	5.73E-03	0	0	loose	bbs_cluster
78	8.01E-05	8.01E-05	7.106	7.106	loose	bbs_cluster
79	1.85E-02	1.20E-02	0	0	loose	bbs_cluster

80	2.49E-02	2.43E-02	0	0	loose	bbs_cluster
81	2.26E-03	8.22E-05	0	10.62	loose	bbs_cluster
82	5.16E-06	6.76E-05	62.286	18.323	loose	bbs_cluster
83	3.34E-03	3.34E-03	0	0	loose	bbs_cluster
84	3.18E-13	3.18E-13	1813.817	1509.925	loose	bbs_cluster
85	4.74E-04	7.01E-06	6.347	83.372	loose	bbs_cluster
86	4.51E-06	2.05E-04	72.715	19.857	loose	bbs_cluster
87	5.40E-08	1.79E-08	169.92	173.738	loose	bbs_cluster
88	2.30E-07	3.09E-07	156.261	210.225	loose	bbs_cluster
89	2.32E-04	8.37E-05	3.634	4.078	loose	bbs_cluster
90	1.14E-06	5.69E-06	81.695	72.526	loose	bbs_cluster
91	1.23E-04	4.57E-05	31.119	59.494	loose	bbs_cluster
92	5.04E-05	7.71E-04	4.298	3.113	loose	bbs_cluster
93	1.95E-06	1.95E-06	23.809	23.31	loose	bbs_cluster
94	1.52E-04	1.22E-04	6.888	6.986	loose	bbs_cluster
95	1.02E-06	1.02E-06	5.991	5.991	loose	bbs_cluster
96	1.94E-03	6.68E-03	0	0	loose	bbs_cluster
97	6.25E-05	6.25E-05	56.701	60.294	loose	bbs_cluster
98	6.52E-13	6.52E-13	1446.237	1308.672	loose	bbs_cluster
99	3.75E-04	3.51E-04	6.477	6.72	loose	bbs_cluster
100	6.24E-03	4.86E-03	0	0	loose	bbs_cluster
1	1.51E-03	9.79E-04	0	3.009	loose	edge_1_300
2	5.43E-03	5.28E-03	0	0	loose	edge_1_300
3	3.80E-02	1.80E-02	0	0	loose	edge_1_300
4	1.85E-03	1.85E-03	0	0	loose	edge_1_300
5	4.62E-07	1.83E-07	48.41	49.275	loose	edge_1_300
6	1.02E-13	1.02E-13	153.847	156.878	loose	edge_1_300
7	5.37E-07	4.53E-07	119.424	143.539	loose	edge_1_300
8	2.00E-03	6.82E-04	0	6.239	loose	edge_1_300
9	1.82E-07	3.16E-08	30.192	42.558	loose	edge_1_300
10	1.04E-02	8.80E-03	0	0	loose	edge_1_300
11	6.02E-02	1.95E-02	0	0	loose	edge_1_300
12	3.00E-03	3.00E-03	0	0	loose	edge_1_300
13	1.06E-05	8.59E-06	116.901	118.274	loose	edge_1_300
14	2.02E-02	2.02E-02	0	0	loose	edge_1_300
15	6.95E-03	5.86E-03	0	0	loose	edge_1_300
16	7.50E-07	7.50E-07	157.137	158.844	loose	edge_1_300
17	1.67E-01	2.66E-02	0	0	loose	edge_1_300
18	2.09E-05	2.09E-05	7.896	7.896	loose	edge_1_300
19	3.63E-06	3.63E-06	74.655	74.655	loose	edge_1_300
20	8.26E-10	5.60E-10	36.914	39.59	loose	edge_1_300
21	6.02E-02	2.86E-02	0	0	loose	edge_1_300
22	3.46E-05	3.29E-05	29.838	34.574	loose	edge_1_300
23	6.74E-02	3.21E-02	0	0	loose	edge_1_300
24	9.45E-02	1.03E-02	0	0	loose	edge_1_300
25	6.27E-09	3.79E-10	514.162	614.591	loose	edge_1_300
26	1.36E-02	5.99E-03	0	0	loose	edge_1_300
27	4.55E-02	2.15E-02	0	0	loose	edge_1_300
28	8.09E-04	8.09E-04	3.092	3.092	loose	edge_1_300

29	7.41E-03	7.41E-03	0	0	loose	edge_1_300
30	1.50E-06	6.56E-07	55.904	76.464	loose	edge_1_300
31	5.39E-03	4.97E-03	0	0	loose	edge_1_300
32	1.15E-02	1.25E-03	0	0	loose	edge_1_300
33	9.36E-08	3.70E-08	62.291	54.814	loose	edge_1_300
34	5.76E-02	2.56E-02	0	0	loose	edge_1_300
35	1.46E-02	1.46E-02	0	0	loose	edge_1_300
36	5.69E-05	7.43E-06	4.245	5.129	loose	edge_1_300
37	5.44E-06	4.68E-06	5.265	5.329	loose	edge_1_300
38	4.60E-04	4.60E-04	43.86	43.86	loose	edge_1_300
39	3.99E-03	3.48E-03	0	0	loose	edge_1_300
40	1.83E-06	1.57E-06	5.738	5.804	loose	edge_1_300
41	3.72E-07	1.17E-07	59.72	64.011	loose	edge_1_300
42	1.29E-02	1.26E-02	0	0	loose	edge_1_300
43	1.54E-02	7.22E-03	0	0	loose	edge_1_300
44	1.07E-03	7.32E-04	0	3.136	loose	edge_1_300
45	5.26E-09	5.26E-09	123.565	123.565	loose	edge_1_300
46	4.67E-03	3.29E-03	0	0	loose	edge_1_300
47	9.91E-05	8.96E-05	54.244	55.03	loose	edge_1_300
48	6.74E-02	3.21E-02	0	0	loose	edge_1_300
49	5.28E-02	2.45E-02	0	0	loose	edge_1_300
50	1.58E-03	1.58E-03	0	0	loose	edge_1_300
51	3.00E-24	3.00E-24	5404.418	3825.18	loose	edge_1_300
52	1.84E-11	1.24E-11	44.101	85.302	loose	edge_1_300
53	9.82E-03	6.95E-03	0	0	loose	edge_1_300
54	7.10E-08	1.85E-08	594.536	678.437	loose	edge_1_300
55	5.10E-03	5.10E-03	0	0	loose	edge_1_300
56	1.99E-02	1.16E-02	0	0	loose	edge_1_300
57	3.61E-05	9.97E-05	217.928	113.261	loose	edge_1_300
58	2.96E-07	2.96E-07	655.413	665.313	loose	edge_1_300
59	7.67E-06	7.28E-06	5.115	5.138	loose	edge_1_300
60	1.04E-08	1.04E-08	118.809	118.809	loose	edge_1_300
61	2.48E-03	1.97E-03	0	0	loose	edge_1_300
62	1.56E-02	1.01E-02	0	0	loose	edge_1_300
63	1.62E-02	4.04E-03	0	0	loose	edge_1_300
64	8.07E-13	8.07E-13	308.581	311.11	loose	edge_1_300
65	6.81E-06	6.81E-06	139.759	145.615	loose	edge_1_300
66	2.98E-03	2.98E-03	0	0	loose	edge_1_300
67	1.54E-02	7.22E-03	0	0	loose	edge_1_300
68	5.28E-02	2.51E-02	0	0	loose	edge_1_300
69	6.29E-05	6.29E-05	103.52	104.853	loose	edge_1_300
70	3.05E-02	1.44E-02	0	0	loose	edge_1_300
71	4.37E-07	3.58E-07	107.831	118.062	loose	edge_1_300
72	2.94E-10	2.94E-10	139.658	139.658	loose	edge_1_300
73	7.24E-03	2.48E-03	0	0	loose	edge_1_300
74	2.64E-03	1.94E-03	0	0	loose	edge_1_300
75	2.87E-04	1.46E-04	3.543	27.312	loose	edge_1_300
76	4.55E-02	2.15E-02	0	0	loose	edge_1_300
77	3.05E-02	1.44E-02	0	0	loose	edge_1_300

78	3.27E-03	2.48E-03	0	0	loose	edge_1_300
79	3.80E-02	1.80E-02	0	0	loose	edge_1_300
80	6.02E-02	2.86E-02	0	0	loose	edge_1_300
81	5.62E-04	3.08E-03	3.25	0	loose	edge_1_300
82	4.48E-09	4.01E-09	209.315	315.175	loose	edge_1_300
83	3.05E-02	1.44E-02	0	0	loose	edge_1_300
84	4.60E-04	4.60E-04	9.435	9.435	loose	edge_1_300
85	6.02E-02	2.86E-02	0	0	loose	edge_1_300
86	6.29E-04	2.74E-04	3.202	3.562	loose	edge_1_300
87	1.04E-09	9.31E-10	224.45	231.742	loose	edge_1_300
88	4.50E-03	1.04E-03	0	0	loose	edge_1_300
89	6.91E-07	1.73E-07	37.717	38.573	loose	edge_1_300
90	6.04E-02	3.43E-02	0	0	loose	edge_1_300
91	3.96E-03	3.96E-03	0	0	loose	edge_1_300
92	1.00E-04	2.24E-05	26.678	31.417	loose	edge_1_300
93	7.05E-02	9.60E-03	0	0	loose	edge_1_300
94	8.91E-04	5.13E-04	15.208	33.926	loose	edge_1_300
95	2.21E-02	2.14E-02	0	0	loose	edge_1_300
96	6.52E-04	2.11E-04	21.727	41.893	loose	edge_1_300
97	3.99E-03	3.48E-03	0	0	loose	edge_1_300
98	4.69E-03	4.69E-03	0	0	loose	edge_1_300
99	8.75E-03	8.33E-03	0	0	loose	edge_1_300
100	4.15E-03	2.31E-04	0	3.636	loose	edge_1_300
1	4.96E-08	3.71E-08	106.171	107.563	loose	gfr
2	3.92E-04	3.92E-04	3.407	6.608	loose	gfr
3	3.20E-06	2.71E-06	120.306	122.191	loose	gfr
4	5.22E-20	5.22E-20	3285.237	2766.554	loose	gfr
5	8.89E-02	4.26E-02	0	0	loose	gfr
6	3.77E-07	2.74E-07	159.019	161.319	loose	gfr
7	2.70E-08	1.25E-08	34.339	25.246	loose	gfr
8	1.28E-05	1.15E-05	92.538	103.047	loose	gfr
9	1.61E-11	1.37E-11	10.794	10.863	loose	gfr
10	5.14E-06	5.14E-06	232.3	242.923	loose	gfr
11	9.29E-04	9.29E-04	6.064	6.064	loose	gfr
12	5.85E-06	4.79E-05	22.747	10.365	loose	gfr
13	1.82E-14	4.78E-11	2121.314	1465.402	loose	gfr
14	4.73E-06	4.73E-06	139.443	141.191	loose	gfr
15	3.03E-04	3.03E-04	6.652	9.88	loose	gfr
16	5.45E-57	5.45E-57	10080.235	7958.513	loose	gfr
17	7.55E-07	1.11E-07	57.771	52.835	loose	gfr
18	3.68E-03	3.53E-03	0	0	loose	gfr
19	1.02E-13	1.02E-13	1917.536	1649.332	loose	gfr
20	2.71E-14	1.77E-14	212.515	215.341	loose	gfr
21	2.82E-18	2.41E-18	489.969	494.935	loose	gfr
22	3.53E-03	1.68E-04	0	7.112	loose	gfr
23	4.49E-03	4.49E-03	0	0	loose	gfr
24	7.79E-05	1.59E-04	36.545	33.471	loose	gfr
25	4.47E-04	4.25E-04	3.35	3.371	loose	gfr
26	6.23E-06	5.81E-06	121.166	126.405	loose	gfr

27	4.46E-06	3.76E-06	285.651	267.307	loose	gfr
28	1.59E-07	1.59E-07	578.567	505.342	loose	gfr
29	1.31E-04	1.07E-04	40.44	60.126	loose	gfr
30	1.22E-02	1.07E-02	0	0	loose	gfr
31	2.04E-02	2.04E-02	0	0	loose	gfr
32	1.65E-04	1.65E-04	3.782	3.782	loose	gfr
33	2.55E-10	2.55E-10	139.839	139.839	loose	gfr
34	8.86E-06	8.86E-06	521.435	438.954	loose	gfr
35	3.05E-02	3.63E-03	0	0	loose	gfr
36	5.25E-03	5.25E-03	0	0	loose	gfr
37	1.76E-03	2.64E-04	0	6.865	loose	gfr
38	2.49E-03	1.74E-03	0	0	loose	gfr
39	6.74E-02	3.21E-02	0	0	loose	gfr
40	1.99E-03	7.63E-03	0	0	loose	gfr
41	4.25E-05	3.17E-05	152.372	173.635	loose	gfr
42	1.27E-04	1.27E-04	3.896	3.896	loose	gfr
43	2.82E-07	2.82E-07	770.869	576.152	loose	gfr
44	2.64E-02	8.55E-03	0	0	loose	gfr
45	1.86E-04	1.31E-04	42.405	42.159	loose	gfr
46	7.49E-07	7.49E-07	430.299	351.053	loose	gfr
47	5.59E-05	5.59E-05	203.548	220.655	loose	gfr
48	5.86E-05	7.83E-05	184.997	160.697	loose	gfr
49	1.04E-09	9.31E-10	197.771	200.325	loose	gfr
50	2.81E-07	1.60E-07	463.153	398.391	loose	gfr
51	3.85E-02	3.85E-02	0	0	loose	gfr
52	0.00E+00	0.00E+00	112.878	112.878	loose	gfr
53	4.86E-04	8.11E-04	15.902	3.091	loose	gfr
54	2.01E-11	2.01E-11	41.994	42.192	loose	gfr
55	5.67E-04	1.45E-03	3.247	0	loose	gfr
56	2.10E-04	2.10E-04	3.678	3.678	loose	gfr
57	4.45E-05	3.64E-05	7.36	13.709	loose	gfr
58	5.81E-04	4.83E-04	15.582	18.756	loose	gfr
59	7.64E-03	2.35E-03	0	0	loose	gfr
60	3.84E-04	3.33E-04	6.456	6.518	loose	gfr
61	1.53E-05	1.53E-05	30.053	8.586	loose	gfr
62	4.08E-03	4.08E-03	0	0	loose	gfr
63	5.28E-02	2.51E-02	0	0	loose	gfr
64	8.92E-04	8.92E-04	9.086	12.114	loose	gfr
65	5.28E-03	5.10E-03	0	0	loose	gfr
66	6.07E-03	3.98E-03	0	0	loose	gfr
67	1.81E-03	1.45E-03	0	0	loose	gfr
68	9.11E-04	9.11E-04	3.04	3.04	loose	gfr
69	3.37E-03	3.37E-03	0	0	loose	gfr
70	3.11E-03	3.11E-03	0	0	loose	gfr
71	5.83E-06	8.76E-07	41.877	50.581	loose	gfr
72	4.55E-02	2.15E-02	0	0	loose	gfr
73	2.45E-07	2.45E-07	96.985	96.985	loose	gfr
74	1.41E-13	1.41E-13	253.118	259.475	loose	gfr
75	2.10E-08	1.80E-08	7.679	7.745	loose	gfr

76	5.69E-07	1.42E-07	218.54	275.402	loose	gfr
77	2.25E-04	2.25E-04	20.369	20.468	loose	gfr
78	1.40E-02	3.63E-03	0	0	loose	gfr
79	9.90E-08	9.90E-08	1536.907	1153.897	loose	gfr
80	3.72E-03	1.25E-03	0	0	loose	gfr
81	2.88E-03	3.69E-04	0	3.433	loose	gfr
82	1.37E-35	1.37E-35	459.197	459.197	loose	gfr
83	7.80E-06	7.80E-06	52.712	94.182	loose	gfr
84	4.55E-02	1.43E-02	0	0	loose	gfr
85	1.41E-03	1.32E-03	0	0	loose	gfr
86	9.14E-02	4.49E-03	0	0	loose	gfr
87	6.13E-03	6.30E-03	0	0	loose	gfr
88	3.23E-02	1.68E-02	0	0	loose	gfr
89	1.87E-03	1.87E-03	0	0	loose	gfr
90	1.84E-03	1.17E-03	0	0	loose	gfr
91	1.12E-11	1.12E-11	344.065	536.911	loose	gfr
92	4.61E-07	4.05E-07	50.586	51.429	loose	gfr
93	9.36E-06	9.36E-06	183.12	170.823	loose	gfr
94	8.36E-03	8.10E-03	0	0	loose	gfr
95	1.56E-02	1.34E-02	0	0	loose	gfr
96	4.59E-04	3.28E-04	6.566	38.098	loose	gfr
97	3.48E-05	2.21E-05	67.274	50.795	loose	gfr
98	6.94E-05	5.19E-05	32.303	32.461	loose	gfr
99	5.11E-04	5.11E-04	12.447	12.447	loose	gfr
100	3.98E-25	3.98E-25	363.653	363.653	loose	gfr
1	5.28E-02	2.51E-02	0	0	loose	pcs
2	3.31E-06	3.31E-06	431.769	483.701	loose	pcs
3	2.03E-02	7.52E-03	0	0	loose	pcs
4	1.97E-04	1.57E-04	10.562	3.804	loose	pcs
5	9.94E-03	3.42E-03	0	0	loose	pcs
6	4.71E-04	1.41E-04	59.521	127.198	loose	pcs
7	9.97E-05	9.97E-05	105.069	106.388	loose	pcs
8	1.61E-03	1.26E-03	0	0	loose	pcs
9	1.08E-03	3.45E-04	0	3.462	loose	pcs
10	3.04E-03	2.57E-03	0	0	loose	pcs
11	3.04E-03	1.94E-03	0	0	loose	pcs
12	4.38E-03	3.98E-04	0	3.4	loose	pcs
13	1.12E-04	8.88E-05	25.503	28.817	loose	pcs
14	4.74E-03	5.16E-04	0	3.287	loose	pcs
15	7.87E-05	7.87E-05	11.123	11.246	loose	pcs
16	3.69E-08	3.27E-08	7.433	7.485	loose	pcs
17	3.44E-06	3.44E-06	5.464	5.464	loose	pcs
18	3.05E-02	1.44E-02	0	0	loose	pcs
19	5.38E-04	5.11E-04	15.977	22.334	loose	pcs
20	8.09E-04	5.65E-04	3.092	3.248	loose	pcs
21	1.55E-02	6.96E-03	0	0	loose	pcs
22	1.81E-07	8.91E-08	112.021	86.303	loose	pcs
23	9.14E-12	9.14E-12	163.346	163.346	loose	pcs
24	3.80E-02	1.80E-02	0	0	loose	pcs

25	4.55E-02	2.15E-02	0	0	loose	pcs
26	6.02E-02	2.86E-02	0	0	loose	pcs
27	2.24E-03	2.24E-03	0	0	loose	pcs
28	1.45E-02	1.45E-02	0	0	loose	pcs
29	3.80E-02	1.80E-02	0	0	loose	pcs
30	5.65E-04	5.65E-04	3.248	3.248	loose	pcs
31	2.78E-03	2.60E-03	0	0	loose	pcs
32	7.46E-02	3.56E-02	0	0	loose	pcs
33	1.61E-02	1.33E-02	0	0	loose	pcs
34	3.04E-03	2.57E-03	0	0	loose	pcs
35	3.04E-03	1.94E-03	0	0	loose	pcs
36	8.57E-04	1.41E-04	3.067	20.301	loose	pcs
37	2.78E-03	2.78E-03	0	0	loose	pcs
38	4.19E-02	1.82E-02	0	0	loose	pcs
39	8.69E-02	4.17E-02	0	0	loose	pcs
40	1.10E-05	1.10E-05	562.299	452.813	loose	pcs
41	8.42E-04	8.42E-04	3.075	3.075	loose	pcs
42	1.95E-04	1.95E-04	13.942	7.172	loose	pcs
43	1.08E-03	1.08E-03	0	0	loose	pcs
44	2.22E-05	1.32E-03	14.47	0	loose	pcs
45	6.47E-03	3.74E-04	0	3.427	loose	pcs
46	1.59E-03	1.59E-03	0	0	loose	pcs
47	3.74E-02	1.56E-02	0	0	loose	pcs
48	2.42E-03	1.77E-04	0	3.753	loose	pcs
49	4.68E-06	4.41E-06	25.061	12.551	loose	pcs
50	5.78E-04	5.78E-04	3.238	12.352	loose	pcs
51	6.49E-04	5.13E-04	18.375	15.448	loose	pcs
52	1.23E-03	9.37E-04	0	3.029	loose	pcs
53	9.91E-05	8.96E-05	54.244	55.03	loose	pcs
54	3.78E-02	3.52E-03	0	0	loose	pcs
55	2.32E-03	1.44E-02	0	0	loose	pcs
56	1.66E-06	2.46E-07	43.034	43.123	loose	pcs
57	2.69E-03	2.38E-03	0	0	loose	pcs
58	6.27E-03	6.04E-03	0	0	loose	pcs
59	3.66E-03	3.66E-03	0	0	loose	pcs
60	9.94E-03	3.42E-03	0	0	loose	pcs
61	7.46E-02	2.99E-03	0	0	loose	pcs
62	5.28E-02	2.51E-02	0	0	loose	pcs
63	3.80E-02	1.80E-02	0	0	loose	pcs
64	2.14E-03	1.01E-02	0	0	loose	pcs
65	1.21E-03	1.02E-03	0	0	loose	pcs
66	5.38E-03	3.49E-03	0	0	loose	pcs
67	1.10E-05	1.10E-05	4.959	8.031	loose	pcs
68	1.70E-04	1.28E-04	7.027	7.184	loose	pcs
69	2.71E-04	2.71E-04	3.568	3.568	loose	pcs
70	1.01E-04	7.62E-07	7.89	14.008	loose	pcs
71	5.82E-03	2.57E-03	0	0	loose	pcs
72	2.28E-06	2.50E-05	75.479	31.12	loose	pcs
73	6.19E-07	6.19E-07	156.748	122.691	loose	pcs

74	3.18E-06	2.69E-06	239.208	194.636	loose	pcs
75	3.66E-05	3.93E-05	58.552	31.341	loose	pcs
76	3.24E-07	3.24E-07	181.631	183.465	loose	pcs
77	5.19E-06	4.27E-06	76.194	77.466	loose	pcs
78	7.39E-05	9.04E-04	20.982	6.045	loose	pcs
79	4.55E-02	2.15E-02	0	0	loose	pcs
80	6.02E-02	2.86E-02	0	0	loose	pcs
81	4.04E-03	4.04E-03	0	0	loose	pcs
82	1.98E-05	1.47E-04	68.61	24.295	loose	pcs
83	2.00E-03	1.94E-03	0	0	loose	pcs
84	6.23E-03	3.99E-03	0	0	loose	pcs
85	4.55E-02	2.15E-02	0	0	loose	pcs
86	5.69E-04	5.69E-04	3.245	3.245	loose	pcs
87	2.66E-03	2.66E-03	0	0	loose	pcs
88	1.49E-06	1.49E-06	143.376	145.039	loose	pcs
89	5.62E-03	3.01E-04	0	3.521	loose	pcs
90	8.59E-03	3.81E-03	0	0	loose	pcs
91	9.07E-03	6.52E-04	0	3.186	loose	pcs
92	1.46E-04	1.46E-04	19.898	20.05	loose	pcs
93	1.90E-06	1.52E-06	423.979	363.544	loose	pcs
94	4.27E-03	4.13E-03	0	0	loose	pcs
95	1.33E-03	1.26E-03	0	0	loose	pcs
96	4.56E-02	1.79E-02	0	0	loose	pcs
97	4.42E-04	4.42E-04	3.355	3.355	loose	pcs
98	1.03E-03	3.35E-04	0	23.475	loose	pcs
99	1.04E-09	9.31E-10	197.771	200.325	loose	pcs
100	3.00E-11	3.00E-11	152.986	152.986	loose	pcs
1	1.63E-07	1.63E-07	40.158	26.085	strict	bbs_cluster
2	5.68E-03	5.39E-03	0	0	strict	bbs_cluster
3	2.39E-02	5.44E-03	0	0	strict	bbs_cluster
4	2.25E-07	8.76E-08	42.77	47.147	strict	bbs_cluster
5	2.30E-09	7.50E-10	57.386	62.681	strict	bbs_cluster
6	1.06E-05	1.06E-05	28.019	28.392	strict	bbs_cluster
7	2.42E-12	1.88E-12	102.914	103.682	strict	bbs_cluster
8	1.02E-08	6.77E-09	157.159	185.332	strict	bbs_cluster
9	1.00E-02	8.70E-04	0	3.06	strict	bbs_cluster
10	3.80E-02	2.03E-02	0	0	strict	bbs_cluster
11	7.46E-02	4.03E-02	0	0	strict	bbs_cluster
12	9.10E-13	9.10E-13	2472.814	2244.547	strict	bbs_cluster
13	1.16E-07	8.44E-08	78.267	78.822	strict	bbs_cluster
14	3.51E-10	2.61E-10	1227.724	1138.746	strict	bbs_cluster
15	5.43E-07	3.65E-07	17.351	18.721	strict	bbs_cluster
16	3.94E-03	1.46E-04	0	9.988	strict	bbs_cluster
17	1.81E-04	1.81E-04	3.743	3.743	strict	bbs_cluster
18	4.49E-06	4.49E-06	61.461	61.461	strict	bbs_cluster
19	6.41E-05	6.41E-05	14.411	17.463	strict	bbs_cluster
20	3.01E-04	1.67E-05	3.521	14.863	strict	bbs_cluster
21	1.91E-02	1.91E-02	0	0	strict	bbs_cluster
22	9.22E-06	7.03E-06	55.06	73.185	strict	bbs_cluster

23	3.48E-04	2.29E-04	9.787	26.483	strict	bbs_cluster
24	1.16E-03	1.52E-04	0	6.854	strict	bbs_cluster
25	6.28E-04	2.20E-06	6.296	58.859	strict	bbs_cluster
26	1.55E-06	1.10E-06	522.417	474.241	strict	bbs_cluster
27	3.20E-03	1.57E-04	0	11.168	strict	bbs_cluster
28	1.35E-06	1.21E-06	9.858	10.005	strict	bbs_cluster
29	1.38E-03	5.44E-03	0	0	strict	bbs_cluster
30	1.87E-05	1.87E-05	274.39	279.657	strict	bbs_cluster
31	1.69E-02	1.29E-02	0	0	strict	bbs_cluster
32	4.58E-02	1.61E-02	0	0	strict	bbs_cluster
33	2.42E-03	1.61E-03	0	0	strict	bbs_cluster
34	3.83E-04	3.83E-04	6.723	6.723	strict	bbs_cluster
35	2.54E-08	2.54E-08	48.978	48.978	strict	bbs_cluster
36	1.01E-03	1.51E-03	0	0	strict	bbs_cluster
37	1.10E-04	9.96E-05	15.08	15.31	strict	bbs_cluster
38	3.52E-05	1.81E-04	14.817	10.323	strict	bbs_cluster
39	4.84E-03	4.84E-03	0	0	strict	bbs_cluster
40	9.57E-06	9.57E-06	51.668	51.668	strict	bbs_cluster
41	1.74E-02	2.74E-03	0	0	strict	bbs_cluster
42	8.11E-04	2.92E-05	3.091	39.406	strict	bbs_cluster
43	4.13E-04	3.04E-04	6.685	6.902	strict	bbs_cluster
44	2.22E-04	2.22E-04	3.654	3.654	strict	bbs_cluster
45	4.74E-16	2.35E-16	249.147	254.963	strict	bbs_cluster
46	3.87E-03	3.36E-03	0	0	strict	bbs_cluster
47	1.60E-02	3.34E-03	0	0	strict	bbs_cluster
48	8.18E-02	4.42E-02	0	0	strict	bbs_cluster
49	1.43E-04	6.38E-06	17.377	38.069	strict	bbs_cluster
50	8.72E-05	8.72E-05	7.351	7.351	strict	bbs_cluster
51	9.08E-05	1.01E-04	7.208	10.357	strict	bbs_cluster
52	1.77E-03	1.58E-04	0	3.803	strict	bbs_cluster
53	1.17E-06	1.17E-06	58.036	63.467	strict	bbs_cluster
54	1.00E-05	4.13E-06	229.232	302.318	strict	bbs_cluster
55	4.99E-04	1.01E-04	6.477	16.886	strict	bbs_cluster
56	2.93E-13	2.93E-13	191.63	193.774	strict	bbs_cluster
57	3.51E-03	1.87E-03	0	0	strict	bbs_cluster
58	2.89E-06	2.89E-06	47.619	60.964	strict	bbs_cluster
59	1.13E-03	1.05E-03	0	0	strict	bbs_cluster
60	1.16E-05	8.39E-06	74.84	82.557	strict	bbs_cluster
61	1.45E-03	1.03E-04	0	10.875	strict	bbs_cluster
62	1.15E-03	3.96E-04	0	3.402	strict	bbs_cluster
63	1.23E-10	1.23E-10	1193.36	1071.108	strict	bbs_cluster
64	3.10E-08	3.10E-08	62.18	62.18	strict	bbs_cluster
65	2.01E-03	2.01E-03	0	0	strict	bbs_cluster
66	7.85E-05	7.85E-05	14.572	14.572	strict	bbs_cluster
67	1.05E-02	1.89E-03	0	0	strict	bbs_cluster
68	7.62E-09	7.62E-09	173.979	178.306	strict	bbs_cluster
69	3.19E-08	3.19E-08	111.227	112.014	strict	bbs_cluster
70	4.13E-03	3.99E-03	0	0	strict	bbs_cluster
71	1.41E-04	7.13E-05	16.958	16.726	strict	bbs_cluster

72	1.81E-06	1.56E-06	10.112	36.621	strict	bbs_cluster
73	3.71E-08	3.71E-08	77.41	79.585	strict	bbs_cluster
74	7.34E-09	3.48E-09	348.572	427.54	strict	bbs_cluster
75	1.42E-04	1.35E-05	23.417	43.136	strict	bbs_cluster
76	3.74E-04	1.03E-03	6.839	0	strict	bbs_cluster
77	2.02E-02	1.82E-02	0	0	strict	bbs_cluster
78	1.21E-04	1.21E-04	13.629	13.645	strict	bbs_cluster
79	1.01E-02	6.79E-03	0	0	strict	bbs_cluster
80	1.10E-01	5.99E-02	0	0	strict	bbs_cluster
81	1.22E-03	1.22E-03	0	0	strict	bbs_cluster
82	5.32E-07	5.32E-07	74.879	69.626	strict	bbs_cluster
83	6.73E-04	4.75E-04	3.172	15.885	strict	bbs_cluster
84	2.84E-15	1.91E-15	1860.402	1691.467	strict	bbs_cluster
85	9.24E-04	1.50E-05	3.034	56.492	strict	bbs_cluster
86	4.98E-08	1.27E-04	67.343	30.196	strict	bbs_cluster
87	1.68E-11	1.68E-11	102.208	103.919	strict	bbs_cluster
88	1.49E-06	1.49E-06	102.1	99.973	strict	bbs_cluster
89	6.00E-05	3.12E-05	7.229	7.514	strict	bbs_cluster
90	1.81E-06	1.81E-06	51.171	47.485	strict	bbs_cluster
91	2.34E-05	2.34E-05	22.332	29.325	strict	bbs_cluster
92	2.07E-04	4.30E-04	3.685	6.443	strict	bbs_cluster
93	7.90E-06	7.90E-06	12.357	32.897	strict	bbs_cluster
94	1.61E-02	1.61E-02	0	0	strict	bbs_cluster
95	4.20E-06	4.20E-06	12.315	12.406	strict	bbs_cluster
96	4.40E-03	2.62E-03	0	0	strict	bbs_cluster
97	6.25E-05	6.25E-05	42.864	42.864	strict	bbs_cluster
98	3.72E-12	3.72E-12	1862.58	1781.229	strict	bbs_cluster
99	3.75E-04	3.28E-04	6.825	6.94	strict	bbs_cluster
100	5.16E-04	4.06E-04	3.287	3.392	strict	bbs_cluster
1	8.65E-03	3.61E-02	0	0	strict	edge_1_300
2	5.28E-02	2.84E-02	0	0	strict	edge_1_300
3	3.80E-02	2.03E-02	0	0	strict	edge_1_300
4	3.35E-03	2.24E-03	0	0	strict	edge_1_300
5	2.40E-07	1.27E-07	60.669	63.457	strict	edge_1_300
6	1.02E-13	1.02E-13	100.767	100.767	strict	edge_1_300
7	1.64E-07	1.32E-07	87.29	88.156	strict	edge_1_300
8	2.64E-03	4.04E-04	0	12.946	strict	edge_1_300
9	5.24E-07	6.56E-09	10.472	22.187	strict	edge_1_300
10	1.36E-02	9.76E-03	0	0	strict	edge_1_300
11	6.02E-02	3.24E-02	0	0	strict	edge_1_300
12	2.04E-03	2.04E-03	0	0	strict	edge_1_300
13	6.29E-05	6.29E-05	27.173	27.362	strict	edge_1_300
14	1.62E-02	1.70E-02	0	0	strict	edge_1_300
15	6.74E-02	3.63E-02	0	0	strict	edge_1_300
16	1.01E-07	1.01E-07	82.398	82.642	strict	edge_1_300
17	4.41E-02	7.75E-03	0	0	strict	edge_1_300
18	2.09E-05	2.09E-05	11.306	11.306	strict	edge_1_300
19	7.36E-06	7.36E-06	54.777	54.777	strict	edge_1_300
20	2.02E-07	1.45E-07	14.775	15.347	strict	edge_1_300

21	6.02E-02	3.24E-02	0	0	strict	edge_1_300
22	4.52E-06	3.45E-06	41.434	43.378	strict	edge_1_300
23	6.74E-02	3.63E-02	0	0	strict	edge_1_300
24	5.96E-02	4.14E-03	0	0	strict	edge_1_300
25	8.70E-10	2.05E-10	474.146	525.612	strict	edge_1_300
26	1.36E-02	3.05E-03	0	0	strict	edge_1_300
27	4.55E-02	2.44E-02	0	0	strict	edge_1_300
28	5.11E-04	4.85E-04	19.198	19.305	strict	edge_1_300
29	5.92E-03	6.20E-03	0	0	strict	edge_1_300
30	4.99E-03	7.30E-04	0	3.137	strict	edge_1_300
31	3.80E-02	2.03E-02	0	0	strict	edge_1_300
32	1.06E-02	1.81E-03	0	0	strict	edge_1_300
33	4.85E-08	2.57E-08	42.923	45.653	strict	edge_1_300
34	1.10E-01	5.99E-02	0	0	strict	edge_1_300
35	2.59E-02	2.59E-02	0	0	strict	edge_1_300
36	2.22E-03	7.10E-04	0	6.298	strict	edge_1_300
37	6.84E-04	6.39E-04	6.219	6.248	strict	edge_1_300
38	4.60E-04	4.60E-04	37.735	37.735	strict	edge_1_300
39	2.34E-03	2.34E-03	0	0	strict	edge_1_300
40	8.06E-03	8.06E-03	0	0	strict	edge_1_300
41	7.45E-08	2.58E-08	52.806	57.105	strict	edge_1_300
42	7.01E-03	4.03E-02	0	0	strict	edge_1_300
43	1.54E-02	8.19E-03	0	0	strict	edge_1_300
44	1.36E-02	1.01E-02	0	0	strict	edge_1_300
45	1.30E-08	1.30E-08	84.581	84.581	strict	edge_1_300
46	9.11E-03	2.14E-03	0	0	strict	edge_1_300
47	1.62E-05	1.62E-05	17.709	17.798	strict	edge_1_300
48	6.74E-02	3.63E-02	0	0	strict	edge_1_300
49	5.28E-02	2.84E-02	0	0	strict	edge_1_300
50	2.24E-03	2.24E-03	0	0	strict	edge_1_300
51	1.77E-22	1.77E-22	5317.62	4583.774	strict	edge_1_300
52	2.05E-08	1.46E-08	17.022	17.601	strict	edge_1_300
53	1.89E-02	4.54E-03	0	0	strict	edge_1_300
54	1.01E-07	2.06E-08	583.982	628.05	strict	edge_1_300
55	1.61E-04	1.61E-04	3.793	3.793	strict	edge_1_300
56	5.28E-02	2.84E-02	0	0	strict	edge_1_300
57	6.02E-05	5.62E-05	171.688	152.206	strict	edge_1_300
58	3.72E-07	2.96E-07	920.55	862.331	strict	edge_1_300
59	9.82E-07	9.82E-07	14.039	14.039	strict	edge_1_300
60	2.58E-08	2.58E-08	81.312	81.312	strict	edge_1_300
61	4.34E-04	4.34E-04	6.433	6.557	strict	edge_1_300
62	8.19E-03	5.70E-03	0	0	strict	edge_1_300
63	3.05E-02	1.63E-02	0	0	strict	edge_1_300
64	3.07E-13	3.07E-13	255.181	255.879	strict	edge_1_300
65	9.29E-07	9.29E-07	89.78	90.263	strict	edge_1_300
66	1.14E-03	1.08E-03	0	0	strict	edge_1_300
67	1.54E-02	8.19E-03	0	0	strict	edge_1_300
68	5.28E-02	2.84E-02	0	0	strict	edge_1_300
69	3.06E-05	3.06E-05	44.888	45.076	strict	edge_1_300

70	3.05E-02	1.63E-02	0	0	strict	edge_1_300
71	1.71E-07	1.55E-07	171.376	175.382	strict	edge_1_300
72	8.85E-10	8.85E-10	96.969	96.969	strict	edge_1_300
73	2.10E-02	1.57E-02	0	0	strict	edge_1_300
74	1.62E-03	1.08E-03	0	0	strict	edge_1_300
75	1.12E-04	6.02E-05	29.142	31.661	strict	edge_1_300
76	4.55E-02	2.44E-02	0	0	strict	edge_1_300
77	3.05E-02	1.63E-02	0	0	strict	edge_1_300
78	4.84E-03	1.28E-03	0	0	strict	edge_1_300
79	3.80E-02	2.03E-02	0	0	strict	edge_1_300
80	6.02E-02	3.24E-02	0	0	strict	edge_1_300
81	3.42E-05	3.03E-05	4.466	4.519	strict	edge_1_300
82	4.84E-10	4.12E-10	191.049	220.315	strict	edge_1_300
83	1.94E-03	9.45E-05	0	4.025	strict	edge_1_300
84	3.43E-04	3.43E-04	22.592	22.614	strict	edge_1_300
85	6.02E-02	3.24E-02	0	0	strict	edge_1_300
86	1.40E-03	1.47E-03	0	0	strict	edge_1_300
87	1.12E-10	9.48E-11	209.236	211.887	strict	edge_1_300
88	2.42E-03	2.95E-04	0	3.53	strict	edge_1_300
89	1.10E-07	4.54E-08	41.105	49.327	strict	edge_1_300
90	2.00E-02	1.14E-02	0	0	strict	edge_1_300
91	6.02E-02	3.24E-02	0	0	strict	edge_1_300
92	5.10E-06	2.21E-06	30.122	32.071	strict	edge_1_300
93	3.01E-02	1.28E-03	0	0	strict	edge_1_300
94	9.37E-04	2.42E-04	33.104	85.771	strict	edge_1_300
95	1.25E-02	3.24E-02	0	0	strict	edge_1_300
96	5.65E-04	1.46E-04	22.108	39.357	strict	edge_1_300
97	2.04E-03	2.04E-03	0	0	strict	edge_1_300
98	1.13E-02	1.13E-02	0	0	strict	edge_1_300
99	3.05E-02	1.63E-02	0	0	strict	edge_1_300
100	9.90E-03	2.98E-03	0	0	strict	edge_1_300
1	2.58E-08	2.58E-08	40.995	41.282	strict	gfr
2	5.15E-04	5.15E-04	3.288	3.288	strict	gfr
3	3.48E-06	3.14E-06	105.992	106.49	strict	gfr
4	1.10E-19	8.61E-20	3624.495	3374.887	strict	gfr
5	8.89E-02	4.82E-02	0	0	strict	gfr
6	2.19E-08	2.19E-08	141.971	142.541	strict	gfr
7	2.84E-09	2.12E-09	46.119	46.455	strict	gfr
8	6.97E-07	6.97E-07	52.038	55.141	strict	gfr
9	2.30E-02	2.24E-02	0	0	strict	gfr
10	4.68E-06	4.68E-06	153.027	143.383	strict	gfr
11	5.67E-04	5.30E-04	15.402	15.445	strict	gfr
12	3.51E-07	2.99E-07	10.752	10.821	strict	gfr
13	6.91E-11	1.65E-10	1849.404	1562.786	strict	gfr
14	6.56E-07	5.85E-07	126.506	127.034	strict	gfr
15	3.74E-04	3.74E-04	9.931	9.931	strict	gfr
16	3.89E-61	3.89E-61	8528.074	7688.955	strict	gfr
17	3.73E-08	1.69E-08	46.613	50.168	strict	gfr
18	1.32E-03	1.24E-03	0	0	strict	gfr

19	6.49E-13	6.49E-13	1578.039	1445.503	strict	gfr
20	1.50E-13	1.50E-13	83.847	84.142	strict	gfr
21	1.70E-19	9.17E-20	329.343	330.673	strict	gfr
22	4.00E-02	3.85E-02	0	0	strict	gfr
23	3.29E-03	3.29E-03	0	0	strict	gfr
24	3.88E-05	3.28E-05	55.052	56.919	strict	gfr
25	6.15E-05	6.15E-05	8.183	8.183	strict	gfr
26	2.79E-05	2.79E-05	32.562	32.788	strict	gfr
27	7.82E-06	7.82E-06	118.286	91.946	strict	gfr
28	4.52E-08	4.52E-08	220.691	184.803	strict	gfr
29	3.16E-05	3.16E-05	34.06	46.377	strict	gfr
30	2.18E-03	1.99E-03	0	0	strict	gfr
31	1.10E-02	1.10E-02	0	0	strict	gfr
32	3.06E-04	3.06E-04	3.514	3.514	strict	gfr
33	6.35E-10	6.35E-10	99.001	99.001	strict	gfr
34	1.03E-05	9.54E-06	317.887	271.003	strict	gfr
35	5.95E-03	5.70E-03	0	0	strict	gfr
36	6.95E-03	6.28E-03	0	0	strict	gfr
37	2.07E-03	1.83E-03	0	0	strict	gfr
38	2.30E-02	1.23E-02	0	0	strict	gfr
39	6.74E-02	3.63E-02	0	0	strict	gfr
40	6.59E-03	1.63E-02	0	0	strict	gfr
41	3.14E-04	3.03E-04	26.953	23.585	strict	gfr
42	1.27E-04	1.27E-04	3.896	3.896	strict	gfr
43	5.40E-07	3.44E-07	372.141	267.817	strict	gfr
44	7.92E-03	2.15E-03	0	0	strict	gfr
45	8.77E-05	8.77E-05	63.669	63.858	strict	gfr
46	6.82E-07	6.82E-07	267.575	238.569	strict	gfr
47	3.06E-05	2.84E-05	420.693	402.069	strict	gfr
48	7.55E-07	6.19E-07	72.539	60.36	strict	gfr
49	1.12E-10	9.48E-11	179.115	179.816	strict	gfr
50	2.82E-06	2.37E-06	81.272	66.173	strict	gfr
51	5.91E-03	5.38E-03	0	0	strict	gfr
52	0.00E+00	0.00E+00	72.96	72.96	strict	gfr
53	4.46E-03	9.26E-04	0	3.033	strict	gfr
54	1.18E-03	1.18E-03	0	0	strict	gfr
55	1.07E-04	1.07E-04	3.972	3.972	strict	gfr
56	2.76E-04	2.76E-04	3.559	3.559	strict	gfr
57	5.06E-03	4.21E-03	0	0	strict	gfr
58	1.21E-04	1.12E-04	66.387	66.54	strict	gfr
59	1.66E-03	1.57E-03	0	0	strict	gfr
60	8.76E-04	8.76E-04	6.065	6.065	strict	gfr
61	4.16E-05	6.03E-05	23.052	13.309	strict	gfr
62	4.69E-03	4.69E-03	0	0	strict	gfr
63	5.28E-02	2.84E-02	0	0	strict	gfr
64	2.41E-04	2.41E-04	20.322	34.013	strict	gfr
65	4.12E-03	2.96E-03	0	0	strict	gfr
66	6.47E-03	6.47E-03	0	0	strict	gfr
67	1.08E-03	8.47E-04	0	6.08	strict	gfr

68	4.85E-04	4.85E-04	3.314	3.314	strict	gfr
69	3.66E-03	3.66E-03	0	0	strict	gfr
70	1.18E-03	1.18E-03	0	0	strict	gfr
71	3.22E-07	1.49E-07	37.508	49.789	strict	gfr
72	4.55E-02	2.44E-02	0	0	strict	gfr
73	6.00E-07	6.00E-07	66.312	66.312	strict	gfr
74	7.04E-14	7.04E-14	199.112	199.921	strict	gfr
75	5.28E-02	2.84E-02	0	0	strict	gfr
76	1.14E-08	8.06E-09	167.718	196.192	strict	gfr
77	2.70E-04	2.55E-04	17.178	17.271	strict	gfr
78	5.20E-03	8.52E-04	0	3.07	strict	gfr
79	1.48E-07	1.48E-07	1543.61	1384.663	strict	gfr
80	1.36E-02	1.01E-02	0	0	strict	gfr
81	2.44E-03	2.44E-03	0	0	strict	gfr
82	1.37E-35	1.37E-35	314.635	314.635	strict	gfr
83	1.03E-04	1.11E-04	15.431	11.749	strict	gfr
84	5.38E-03	1.20E-03	0	0	strict	gfr
85	8.82E-04	8.10E-04	3.054	3.091	strict	gfr
86	5.55E-02	2.60E-03	0	0	strict	gfr
87	1.27E-02	1.67E-03	0	0	strict	gfr
88	8.18E-02	4.42E-02	0	0	strict	gfr
89	2.00E-03	1.94E-03	0	0	strict	gfr
90	9.11E-04	8.42E-04	3.04	3.075	strict	gfr
91	6.58E-12	6.58E-12	291.931	297.281	strict	gfr
92	5.78E-08	5.78E-08	74.241	77.509	strict	gfr
93	1.27E-05	1.18E-05	368.324	347.782	strict	gfr
94	4.66E-03	1.13E-03	0	0	strict	gfr
95	1.03E-03	9.83E-04	0	3.008	strict	gfr
96	2.99E-04	2.85E-04	47.26	48.617	strict	gfr
97	1.06E-04	9.41E-05	72.693	65.685	strict	gfr
98	4.08E-04	3.44E-04	46.776	46.885	strict	gfr
99	6.82E-04	4.85E-04	27.583	40.565	strict	gfr
100	3.98E-25	3.98E-25	262.719	262.719	strict	gfr
1	5.28E-02	2.84E-02	0	0	strict	pcs
2	3.84E-06	1.51E-06	368.925	425.024	strict	pcs
3	1.79E-02	3.16E-03	0	0	strict	pcs
4	4.08E-04	3.84E-04	9.851	9.878	strict	pcs
5	8.76E-04	8.76E-04	3.057	3.057	strict	pcs
6	2.74E-04	4.06E-05	6.78	47.314	strict	pcs
7	5.62E-05	5.62E-05	14.729	14.85	strict	pcs
8	2.60E-03	9.04E-04	0	6.088	strict	pcs
9	1.08E-03	8.04E-04	0	9.11	strict	pcs
10	2.64E-03	9.83E-04	0	3.008	strict	pcs
11	3.28E-03	2.14E-03	0	0	strict	pcs
12	2.78E-03	1.61E-03	0	0	strict	pcs
13	1.21E-05	9.87E-06	22.946	23.036	strict	pcs
14	4.55E-02	2.44E-02	0	0	strict	pcs
15	8.74E-05	8.30E-05	7.518	7.569	strict	pcs
16	2.18E-02	6.95E-03	0	0	strict	pcs

17	5.49E-06	5.10E-06	5.26	5.293	strict	pcs
18	3.05E-02	1.63E-02	0	0	strict	pcs
19	4.85E-04	4.85E-04	90.976	162.554	strict	pcs
20	3.05E-02	1.63E-02	0	0	strict	pcs
21	2.45E-03	2.33E-03	0	0	strict	pcs
22	8.07E-06	8.07E-06	74.434	63.309	strict	pcs
23	2.77E-11	2.77E-11	113.505	113.505	strict	pcs
24	3.80E-02	2.03E-02	0	0	strict	pcs
25	4.55E-02	2.44E-02	0	0	strict	pcs
26	6.02E-02	3.24E-02	0	0	strict	pcs
27	8.49E-04	8.49E-04	6.115	6.115	strict	pcs
28	1.45E-02	1.45E-02	0	0	strict	pcs
29	3.80E-02	2.03E-02	0	0	strict	pcs
30	6.52E-04	6.52E-04	3.186	3.186	strict	pcs
31	1.75E-03	1.60E-03	0	0	strict	pcs
32	7.46E-02	4.03E-02	0	0	strict	pcs
33	6.02E-02	3.24E-02	0	0	strict	pcs
34	1.62E-03	8.04E-04	0	9.11	strict	pcs
35	1.62E-03	1.08E-03	0	0	strict	pcs
36	3.60E-03	3.69E-04	0	39.211	strict	pcs
37	2.98E-03	2.78E-03	0	0	strict	pcs
38	2.16E-03	2.16E-03	0	0	strict	pcs
39	1.44E-01	7.91E-02	0	0	strict	pcs
40	1.22E-06	1.18E-05	513.614	460.986	strict	pcs
41	9.11E-04	8.76E-04	3.04	3.057	strict	pcs
42	7.79E-05	6.72E-05	29.353	26.606	strict	pcs
43	1.18E-03	1.08E-03	0	0	strict	pcs
44	1.55E-04	1.55E-04	7.438	7.438	strict	pcs
45	5.34E-03	2.99E-03	0	0	strict	pcs
46	3.22E-03	3.03E-03	0	0	strict	pcs
47	8.18E-02	4.42E-02	0	0	strict	pcs
48	3.80E-02	2.03E-02	0	0	strict	pcs
49	8.86E-05	8.86E-05	11.375	7.98	strict	pcs
50	2.00E-04	2.00E-04	10.673	10.979	strict	pcs
51	9.83E-04	9.83E-04	3.008	6.015	strict	pcs
52	1.23E-03	1.23E-03	0	0	strict	pcs
53	1.62E-05	1.62E-05	17.709	17.798	strict	pcs
54	1.13E-02	1.78E-03	0	0	strict	pcs
55	9.83E-04	9.83E-04	3.008	3.008	strict	pcs
56	1.29E-05	5.41E-06	15.768	16.774	strict	pcs
57	2.02E-04	2.16E-03	3.696	0	strict	pcs
58	1.23E-03	1.23E-03	0	0	strict	pcs
59	8.00E-03	8.00E-03	0	0	strict	pcs
60	3.05E-02	1.63E-02	0	0	strict	pcs
61	1.13E-02	6.99E-04	0	9.271	strict	pcs
62	1.79E-02	6.90E-03	0	0	strict	pcs
63	3.80E-02	2.03E-02	0	0	strict	pcs
64	3.48E-03	3.35E-03	0	0	strict	pcs
65	3.05E-02	1.63E-02	0	0	strict	pcs

66	4.55E-02	2.44E-02	0	0	strict	pcs
67	1.10E-05	1.10E-05	4.959	4.959	strict	pcs
68	9.83E-04	8.31E-04	6.014	6.088	strict	pcs
69	4.08E-04	3.59E-04	3.39	3.445	strict	pcs
70	2.84E-05	1.76E-05	4.546	4.755	strict	pcs
71	3.20E-03	8.04E-04	0	3.095	strict	pcs
72	2.04E-05	2.04E-05	68.53	61.406	strict	pcs
73	8.22E-07	8.22E-07	187.182	155.771	strict	pcs
74	5.52E-08	5.52E-08	61.811	48.499	strict	pcs
75	4.76E-05	1.32E-05	70.666	84.938	strict	pcs
76	4.34E-08	4.34E-08	112.265	112.754	strict	pcs
77	1.28E-06	1.15E-06	61.161	64.867	strict	pcs
78	3.84E-05	3.42E-05	8.713	8.763	strict	pcs
79	4.55E-02	2.44E-02	0	0	strict	pcs
80	6.02E-02	3.24E-02	0	0	strict	pcs
81	2.60E-03	2.60E-03	0	0	strict	pcs
82	5.93E-05	2.06E-05	37.536	30.465	strict	pcs
83	9.83E-04	9.83E-04	6.015	6.015	strict	pcs
84	4.69E-03	3.48E-03	0	0	strict	pcs
85	4.55E-02	2.44E-02	0	0	strict	pcs
86	1.51E-03	1.51E-03	0	0	strict	pcs
87	2.04E-03	1.85E-03	0	0	strict	pcs
88	2.00E-07	2.00E-07	69.474	69.685	strict	pcs
89	3.09E-03	1.19E-04	0	3.925	strict	pcs
90	3.92E-03	1.20E-03	0	0	strict	pcs
91	3.83E-03	7.44E-04	0	3.128	strict	pcs
92	6.02E-05	6.02E-05	4.22	4.22	strict	pcs
93	7.21E-05	1.06E-04	48.592	43.062	strict	pcs
94	1.58E-03	1.58E-03	0	0	strict	pcs
95	1.54E-03	9.59E-04	0	3.018	strict	pcs
96	6.07E-03	2.98E-03	0	0	strict	pcs
97	2.31E-04	2.31E-04	7.008	7.008	strict	pcs
98	6.00E-03	4.43E-03	0	0	strict	pcs
99	1.12E-10	9.48E-11	179.115	179.816	strict	pcs
100	3.00E-11	3.00E-11	108.853	108.853	strict	pcs

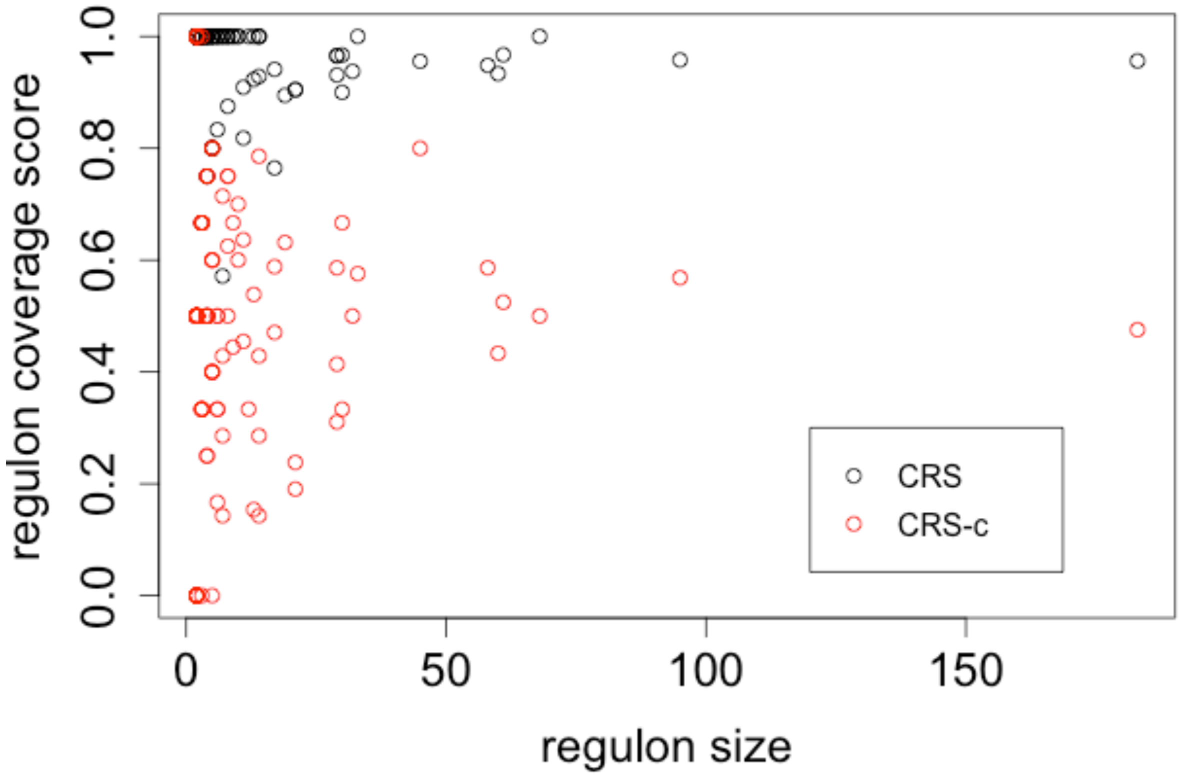

Figure S1. The coverage scores of all the known regulons in terms of the top 100 CRS clusters. The red and black dots are coverage scores calculated based on clique clusters (CRS-c) and optimized clusters (CRS), respectively.

Appendix 1: The implementation of the new regulon prediction method on DIMINDA web server.

To facilitate the users applying our new method to predict regulons on bacterial genomes, we have implemented it in integrated motif identification and analyses web server, DMINDA. We listed all 2,072 sequenced prokaryotic genomes, and the users can get regulon prediction results on them by several clicks as following.

Step 1: Go to the front page of DMINDA (http://csbl.bmb.uga.edu/DMINDA/), and click on the button "New function for REGULON prediction!"

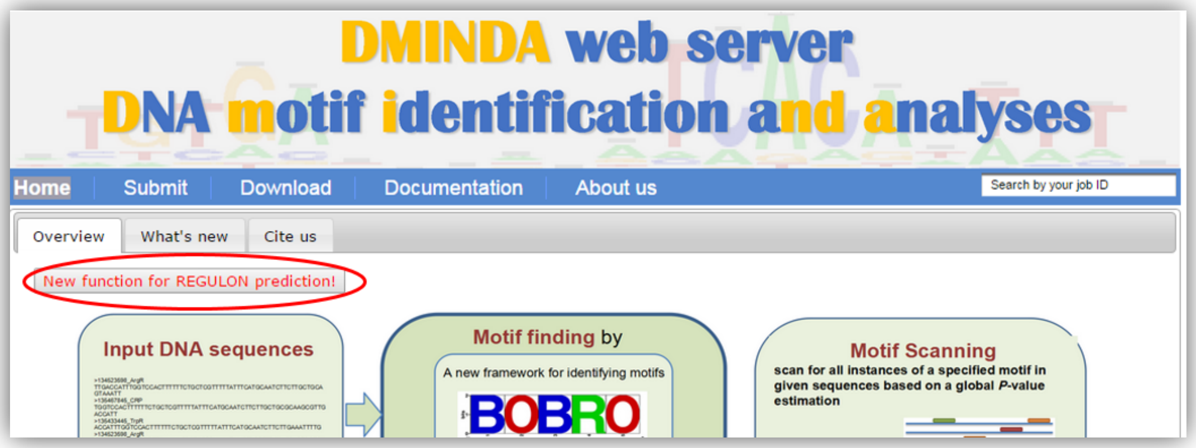

Step 2: A start page will provide a list including all the currently available 2,072 organisms with statistics about their genes, operons and non-coding regions. The users can select any interested genomes to predict regulons, e.g 'NC_000913' or '*Escherichia coli* K-12 MG1655' as following:

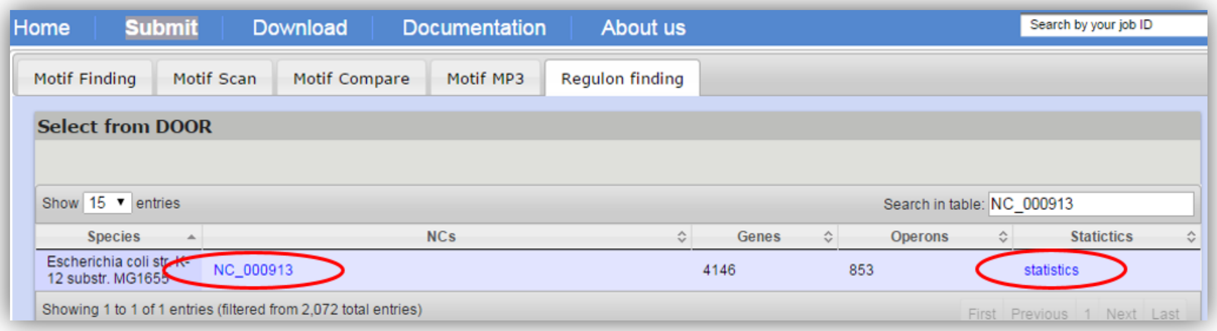

Step 3: In the next page, the web server will automatically upload all operons of selected genome. Also, the users have alternative option to upload the operons they are interesting in. Now click on "Submit" to run the regulon prediction job. Here the user has the option to enter an email address for results retrieval if he prefers.

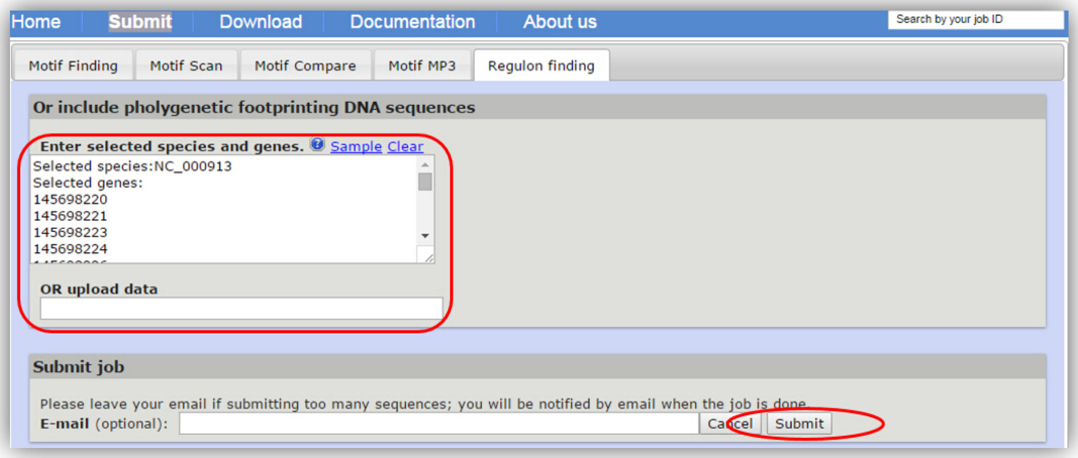

We take a set of *E. coli* K12 operons as an example to illustrate the regulon prediction results which can be retrieved by entering the job ID 2015112205052g into the searching box on our server. A result page lists the predicted regulons with their included operons. Besides, we also expand the predicted regulons by searching new member based on the corresponding motif profiles, which can be seen by click another tab 'expanded cluster'.

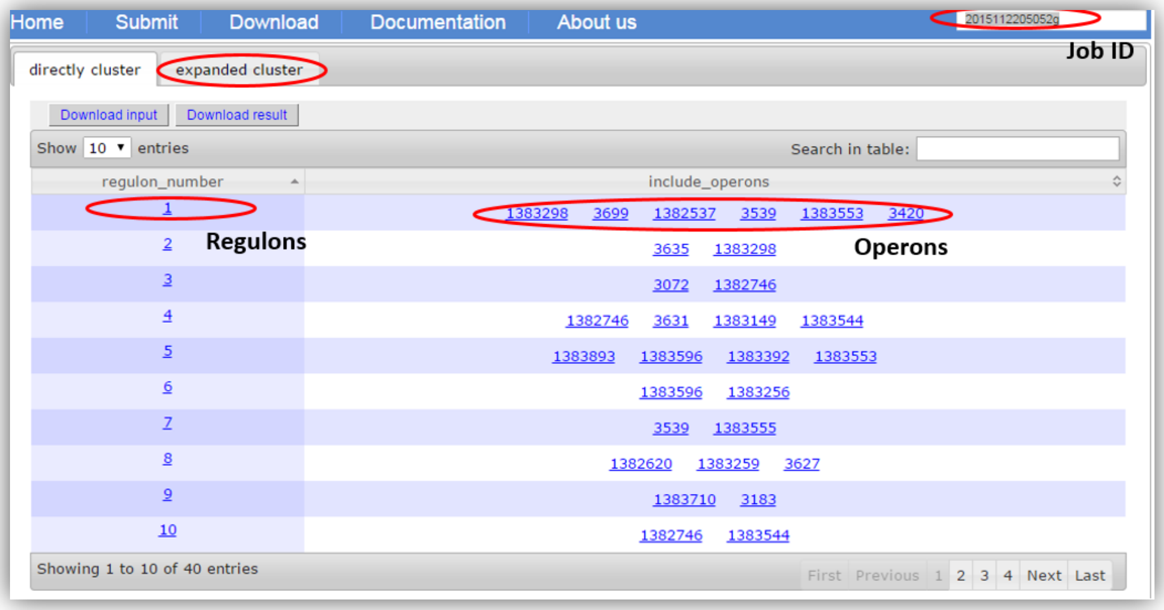

Furthermore, the users can click on any list regulons or operon for more detail information. For regulons, we provide the details about its members and their regulatory region sequences:

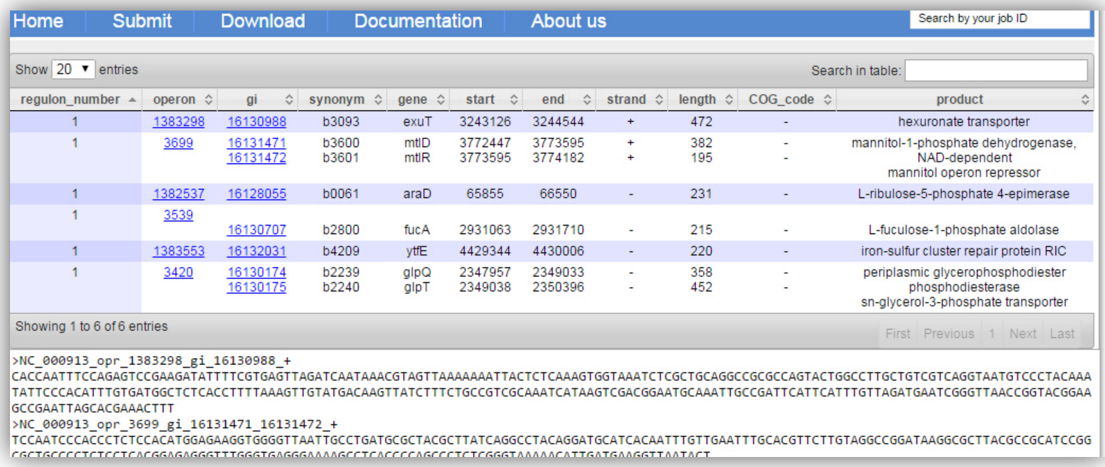

For the operons in regulons, we provide extra information about their predicted motifs, including motif logo, width, p-value, motif instances and its locations as following:

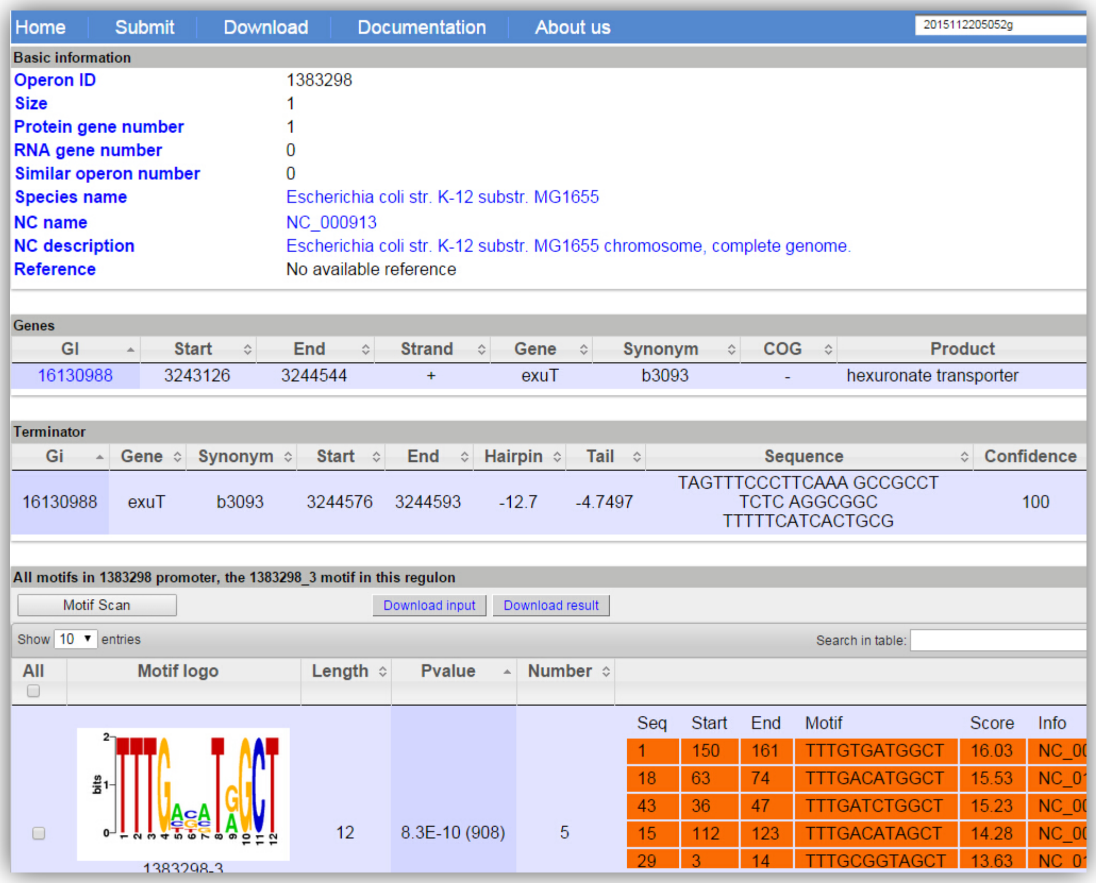

Supplement: Supplementary Information [file srep23030-s1.pdf]
